# Time-of-day effects of acute and regular exercise on sleep quality and mood: a three-level meta-analysis

**DOI:** 10.3389/fpsyg.2026.1933599

**Published:** 2026-09-25

**Authors:** Xuanming Hu, Peiyou Chen

**Affiliations:** School of Sports Science and Physical Education, Nanjing Normal University, Nanjing, Jiangsu, China

**Keywords:** chronobiology, circadian rhythm, exercise timing, mood, physical activity, precision exercise prescription, sleep quality, three-level meta-analysis

## Abstract

**Background:**

Evidence regarding whether exercise timing differentially affects sleep quality and mood remains inconsistent. This study examined the effects of acute and regular exercise performed at different times of day on sleep and mood.

**Methods:**

Eight English and Chinese databases were searched from inception to June 2026. Parallel and crossover randomized trials were synthesized using three-level meta-analysis with non-diagonal sampling variance-covariance matrices. Exercise vs. Control and direct Time vs. Time comparisons were analyzed separately, with CR2 cluster-robust inference used as a robustness check.

**Results:**

Thirteen studies contributed 81 effect sizes (40 sleep and 41 mood). For sleep, Exercise vs. Control showed a large effect in the conventional model (g = 0.86, 95% CI [0.26, 1.46], *p* = 0.005), but CR2 inference was non-significant (*p* = 0.109). Direct Time vs. Time comparisons were non-significant (g = 0.15, 95% CI [−0.08, 0.39], *p* = 0.209; CR2 *p* = 0.127), with no stable advantage for morning, afternoon, or evening exercise. For mood, Exercise vs. Control was large but non-significant (g = 0.95, *p* = 0.056). Time vs. Time was significant conventionally (g = 0.30, *p* = 0.027) but not under CR2 inference (*p* = 0.097). Afternoon vs. Evening favored afternoon exercise (g = 0.50, *p* = 0.001), although based on only two studies.

**Conclusion:**

Exercise itself may be more important than its specific timing for sleep, whereas mood may show timing-related signals that remain insufficiently robust for definitive recommendations.

**Systematic review registration:**

https://www.crd.york.ac.uk/prospero/, identifier [CRD420261431622].

## 1 Introduction

Whether exercise performed at different times of the day exerts differential effects on sleep quality and mood states remains a long-standing, unresolved critical issue in the field of precision exercise prescription (Lewis et al., 2018). Although the overall efficacy of exercise as a non-pharmacological intervention for improving sleep and mood is widely supported (Kredlow et al., 2015; Schuch et al., 2016; Gordon et al., 2017), evidence concerning the optimal timing of exercise remains highly divergent. Some studies have found that morning exercise can shorten sleep onset latency and reduce subjective insomnia severity (Seol et al., 2021), whereas others indicate that afternoon or early evening exercise yields more pronounced improvements in sleep efficiency (Vlahoyiannis et al., 2021). Furthermore, certain research suggests that for patients with chronic insomnia, moderate-intensity morning exercise may be less effective in improving sleep than evening exercise (Passos et al., 2011). In the psychological domain, although certain trials suggest that a single bout of morning exercise more substantially enhances positive affect (Maraki et al., 2005), an individual’s chronotype is intrinsically linked to mood states (Hsu et al., 2012). This implies that aligning exercise timing with the biological clock may influence emotional outcomes, despite a current lack of direct interventional evidence. The roots of these discrepancies can largely be attributed to the heterogeneity of intervention characteristics. The acute physiological perturbations triggered by a single bout of exercise (e.g., post-elevation overshoot drop in core body temperature, adenosine accumulation, and autonomic rebound) and the adaptive remodeling induced by long-term regular training (e.g., altered sensitivity of the hypothalamic-pituitary-adrenal axis, gradual phase-shifting of circadian rhythms) operate through fundamentally distinct mechanisms (Myllymäki et al., 2011; Beserra et al., 2018; Frimpong et al., 2021). However, previous quantitative syntheses have rarely incorporated both within a unified comparative framework. This fragmented evidentiary landscape leaves clinical practice without clear guidelines regarding “whether acute exercise can serve as a same-day sleep aid” and “at which specific time of day regular exercise should be scheduled to maximize cumulative benefits” (Bull et al., 2020).

While several existing quantitative syntheses have provided an important foundation for clarifying these issues, salient gaps remain. In the sleep domain, a meta-analysis by Stutz published in Sports Medicine systematically evaluated the effects of evening exercise on sleep architecture in healthy populations, revealing that low-to-moderate intensity evening exercise does not impair sleep quality and may even increase the proportion of slow-wave sleep—a finding that challenges the traditional paradigm that “evening exercise inevitably disrupts sleep” (Stutz et al., 2019). However, this meta-analysis solely focused on the single timeframe of the evening, failing to incorporate morning and afternoon sessions for parallel comparison, nor did it encompass mood-related outcomes. Early quantitative reviews by Kubitz et al. (1996) and Youngstedt et al. (1997) investigated the moderating effect of exercise timing on post-acute exercise sleep; however, limited by their publication dates, neither addressed the evidence regarding long-term, regular exercise interventions (Kubitz et al., 1996; Youngstedt et al., 1997). In the psychological domain, although systematic reviews have explored the ameliorative effects of exercise interventions on psychological distress and mood disorders, to date, no quantitative synthesis has included exercise timing as a moderating variable in the analysis (Schuch et al., 2016; White et al., 2017). Whether exercise timing exerts an independent moderating effect on emotional outcomes remains an unresolved question.

More critically, the aforementioned reviews universally employed traditional two-level random-effects models, which fail to account for the statistical dependence introduced when a single study contributes multiple effect sizes—such as from different timing groups, concurrent reporting of multiple outcomes, or sharing a common control group. This ignored dependency structure leads to an underestimation of standard errors, thereby artificially inflating the statistical significance of the effects and increasing the risk of Type I errors (Van den Noortgate et al., 2013; Cheung, 2019).

A three-level meta-analytic model addresses this by partitioning the sources of variance into sampling variance (Level 1), within-study variance among effect sizes.

## 2 Methods

### 2.1 Protocol and registration

The protocol for this study has been registered on the PROSPERO International Prospective Register of Systematic Reviews (registration number: CRD420261431622) and was conducted and reported in strict accordance with the Preferred Reporting Items for Systematic Reviews and Meta-Analyses (PRISMA 2020) guidelines (Page et al., 2021).

### 2.2 Search strategy

To ensure the comprehensiveness of the literature retrieval and minimize language bias, we systematically searched the following eight core English and Chinese electronic databases: the English databases included PubMed, Embase, Cochrane Library (CENTRAL), Web of Science (Core Collection), APA PsycInfo (EBSCOhost), and SportDiscus (EBSCOhost); the Chinese databases included the China National Knowledge Infrastructure (CNKI) and Wanfang Data. The search timeframe was restricted from database inception to June 2026, with no language restrictions.

The search utilized a combination of subject headings (e.g., MeSH) and free-text words using Boolean logic. The core English search strategy was constructed around four thematic dimensions: (1) Intervention-related terms: exercise OR “physical activity” OR “resistance training” OR aerobic OR yoga OR “high-intensity interval” OR HIIT OR “structured training”; (2) Timing-related terms: “time of day” OR diurnal OR morning OR afternoon OR evening OR “circadian rhythm” OR chronobiology OR timing; (3) Outcome-related terms: sleep OR “sleep quality” OR polysomnography OR actigraphy OR “sleep efficiency” OR “sleep onset latency” OR mood OR affect OR depression OR anxiety OR POMS OR DASS-21 OR PANAS; (4) Study design-related terms: randomized OR randomized OR randomly OR RCT OR crossover OR cross-over. The search strategy for the Chinese databases was constructed using corresponding Chinese terms for the aforementioned core concepts. The complete search strategies for each database are detailed in the Supplementary File 1. Additionally, we manually performed backward citation tracking on the reference lists of all included studies, as well as previously published systematic reviews and meta-analyses in this field, to identify any potentially eligible studies that might have been missed.

### 2.3 Eligibility criteria

The inclusion and exclusion criteria were strictly formulated according to the PICOS (Population, Intervention, Comparison, Outcome, Study design) framework, detailed as follows:

#### 2.3.1 Population

Individuals of any age group (including adolescents, adults, and older adults) were included. This study imposed no strict restrictions on the participants’ health status or baseline sleep/mood levels, thereby encompassing both healthy populations and individuals with mild sleep disorders or mood issues. For studies exclusively enrolling patients with severe psychiatric or sleep disorders requiring pharmacological intervention (e.g., severe depression or severe insomnia requiring clinical medication), we did not exclude them; however, these studies were specifically flagged during data extraction to facilitate subsequent subgroup or sensitivity analyses.

#### 2.3.2 Intervention

The included interventions had to be “structured exercise” (e.g., aerobic exercise, resistance training, yoga, high-intensity interval training, etc.), explicitly excluding mere activities of daily living (e.g., household chores, active commuting). The exercise had to be performed within the following predefined specific time windows:

Morning: Start time between 06:00 and 10:00, or from waking up until before 12:00;

Afternoon: Start time between 12:00 and 16:00;

Evening: Start time between 17:00 and 21:00, and at least 2 h prior to bedtime.

Intervention duration was categorized into acute (a single bout of exercise) and regular exercise (intervention period ≥ 2 weeks). Both types of data were included in the analysis, and the potential impact of intervention duration on effect estimation was further explored where data permitted.

For original studies that only labeled the exercise timing as “AM” or “PM” without providing specific clock times, we established the following classification rules: If a study reported “AM” and provided no end time or bedtime, it was by default categorized as morning; if it reported “PM” and the intervention start time was not explicitly between 12:00 and 16:00, the categorization was determined based on other contextual clues in the literature (such as typical afternoon or evening routines). The rationale for classification is detailed on a study-by-study basis in the Supplementary materials. All classification decisions were comprehensively documented in the data extraction forms to serve as a basis for inter-rater consistency checks.

#### 2.3.3 Comparison

Studies meeting any one of the following three types of comparisons were eligible for inclusion:

Comparisons between different exercise timing groups: e.g., direct comparisons between morning and evening exercise;

Comparisons between exercise at a specific time and non-exercise controls: e.g., exercise at a specific time compared with no exercise or activities of daily living;

Comparisons between exercise at a specific time and active interventions: e.g., exercise compared with other non-exercise adjunctive interventions.

For crossover trials, comparisons between different time windows were treated as paired designs, and within-participant correlations were accounted for in the calculation of effect sizes and their sampling variances. For scenarios involving shared control groups or multiple correlated effect sizes contributed by a single study, the statistical dependence was addressed in the subsequent three-level meta-analysis by constructing an off-diagonal sampling variance-covariance matrix.

#### 2.3.4 Outcome

Studies had to report at least one of the following primary outcomes:

Sleep quality: Including subjective sleep quality (e.g., using the PSQI scale) or objective sleep metrics [e.g., sleep efficiency (SE), sleep onset latency (SOL), and total sleep time (TST) measured via polysomnography (PSG) or actigraphy].

Mood state: Including indices measured using standardized mood scales (e.g., POMS, DASS-21, PANAS).

Secondary outcomes [e.g., Epworth Sleepiness Scale (ESS), exercise adherence] were also included in the analysis if reported in the literature.

#### 2.3.5 Study design

Only randomized controlled trials (RCTs), including parallel and randomized crossover designs, were included. Quasi-experimental studies, single-arm intervention studies, and observational studies (e.g., cross-sectional or cohort studies) were excluded.

### 2.4 Study selection

Study selection followed the standard procedures outlined in the PRISMA 2020 guidelines. All records retrieved from the database searches were imported into the EndNote X9 reference management software. Duplicate records were first removed using the automatic deduplication function, followed by an initial screening based on titles and abstracts. Two trained reviewers independently conducted the title/abstract screening, excluding literature that was obviously irrelevant, did not fit the PICOS framework, or fell under the exclusion criteria. For articles passing the initial screening, full texts were obtained and independently reviewed by the same two researchers to determine the final eligible studies for inclusion in this meta-analysis. Any discrepancies arising during the screening process were resolved through discussion and consensus; if an agreement could not be reached, a third researcher made the final adjudication.

### 2.5 Data extraction

Two reviewers independently extracted data from each eligible article using a pre-designed, standardized electronic data extraction form that was pilot-tested on a random sample of five included studies. The extraction form was revised based on feedback from the pilot test to improve clarity and completeness. Data extracted by the two reviewers were cross-checked prior to formal discussion, and any discrepancies were resolved through discussion or consultation with a third reviewer. When the same study reported duplicate data across different publications, only the version with the most complete data or the largest sample size was included to avoid double counting.

The extraction form covered basic study information, participant characteristics, exercise intervention characteristics, control group characteristics, and outcome indicator characteristics. Detailed characteristics of the included studies are presented in Table 1.

**TABLE 1:** Characteristics of included studies.

Study (country)	Design	Participants (*N*, age, % F, status)	Protocol (type, intensity, duration)	Period	Exercise timing (comparisons)	Outcomes
Iyer et al., 2026 (India)	RCT	*N* = 82; 22.5 years; 49% F; students (PSQI > 5)	Yoga, light-mod, 60 min	Reg (4 weeks)	AM vs. PM vs. control	Sleep, mood
Saidi et al., 2021 (France)	RCT	*N* = 28; 54.1 years; 72% F; sedentary, overweight/obese	Aerobic + resist, mod, 90 min	Reg (12 weeks)	AM (09:00) vs. PM	Sleep, mood
Shen et al., 2025 (China)	RCT	*N* = 58; 22.9 yeas; 0% F; sedentary healthy males	Aerobic, mod, ≥ 150 min/week	Reg (12 weeks)	AM (06:00) vs. PM vs. control	Sleep
Arciero et al., 2022 (USA)	RCT	*N* = 47; ∼43.0 years; 57% F; active healthy	Multimodal RISE, mod-vig, <60 min	Reg (12 weeks)	AM (06:30) vs. PM	Mood
Seol et al., 2021 (Japan)	RCT	*N* = 49; 71.0 years; 75% F; older adults (sleep complaints)	Aerobic (step), low, 30 min	Reg (8 weeks)	AM (waking) vs. PM	Sleep
Vlahoyiannis et al., 2021 (Brazil)	RCT	*N* = 19; 45.0 years; 79% F; sedentary, chronic insomnia	Aerobic (treadmill), mod, 50 min	Reg (26 weeks)	AM (10:00) vs. late-PM	Sleep, Mood
O’Connor and Davis, 1992 (USA)	Crossover	*N* = 12; 23.2 years; 0% F; healthy males	Treadmill, mod-vig, 20 min	Acute	08:00 vs. 12:00 vs. 16:00 vs. 20:00	Mood
Teo et al., 2021 (Australia)	RCT	*N* = 40; 51.0 years; 58% F; sedentary, overweight/obese	Aerobic + resist, mod-vig, 60 min	Reg (12 weeks)	AM (08:00) vs. PM	Sleep
Vlahoyiannis et al., 2021 (Cyprus)	Crossover	*N* = 10; 23.9 years; 0% F; active males	Cycling (HIIT/MICT), mod-vig, 45 min	Acute	PM (19:00) vs. control	Sleep
Vitale et al., 2017 (Italy)	Crossover	*N* = 23; 22.0 years; 0% F; trained soccer players	HIIT run, high, 25 min	Acute	AM (08:00) vs. PM (by chronotype)	Sleep
Maraki et al., 2005 (UK)	Crossover	*N* = 12; 28.0 years; 100% F; Inactive females	Aerobic + resist, mod, 60 min	Acute	AM vs. PM vs. control	Mood
Saidi et al., 2023 (France)	Crossover	*N* = 42; 16.5 years; 0% F; adolescent rugby players	HIIT (rugby), high, 90 min	Acute	13:00 vs. 17:30 (by chronotype)	Sleep, mood
Fairbrother et al., 2014 (USA)	Crossover	*N* = 20; 41.9 years; NR; prehypertensive	Treadmill, mod, 30 min	Acute	07:00 vs. 13:00 vs. 19:00	Sleep

*N*, final analyzed sample size; F, female; NR, not reported; RCT, parallel randomized controlled trial; Crossover, randomized crossover trial; Reg, regular intervention; AM: Morning; PM: Afternoon/Evening; Mod, moderate intensity; Vig, vigorous intensity. Timing abbreviations (AM/PM) are used when specific clock hours were not strictly delimited or when encompassing broader blocks (e.g., waking–12:00).

For each eligible comparison, the post-intervention mean, standard deviation (SD), and sample size for each group were extracted. For studies reporting standard errors (SEM), the SEM was converted to SD using the formula SD = SEM ×  N. For studies reporting outcomes only as medians and interquartile ranges, the mean and SD were estimated using the methods recommended by Hozo et al. (2005), Wan et al. (2014). All estimated data were explicitly marked in the dataset, and their potential impact on the overall effect estimation was evaluated through sensitivity analyses where data permitted.

When a single article reported multiple effect sizes (e.g., multiple outcome indicators, multiple comparison groups, or multiple measurement time points), all eligible effect sizes were extracted and assigned a unique effect size identifier. Statistical dependencies arising from multiple effect sizes within the same study, shared control groups, and repeated measurements were addressed by constructing an off-diagonal sampling variance-covariance matrix. Effect sizes were then nested within studies in a three-level meta-analytic model to separately estimate within-study and between-study variance. Furthermore, cluster-robust variance estimation with a small-sample correction, clustered by Study_ID, was implemented as a robustness check for the main model inferences.

### 2.6 Risk of bias assessment

The risk of bias of the included studies was assessed using the Cochrane RoB 2 tool. For parallel randomized controlled trials, the standard RoB 2 was used (Sterne et al., 2019); for randomized crossover trials, the crossover-specific version was used. Both versions cover five domains: randomization process, deviations from intended interventions, missing outcome data, measurement of the outcome, and selection of the reported result. Additional risks specific to crossover trials (e.g., adequacy of the washout period, carryover effects, period effects) were integrated into the judgment of corresponding domains rather than listed as a separate Supplementary checklist. The assessment was independently conducted by two reviewers, and discrepancies were resolved through discussion. The final overall risk of bias judgment followed the RoB 2 guidelines: overall “low risk” if all domains were at low risk; “some concerns” if at least one domain had some concerns and no domain was at high risk; and “high risk” if any domain was at high risk.

In addition to the standard domains assessed by the RoB 2 tool, this study paid additional attention to methodological risks specific to crossover designs. Specifically, we reviewed whether each crossover study reported: (1) the duration and adequacy of the washout period (≥48 h); (2) whether statistical tests or design controls were implemented for potential carryover effects; and (3) how period effects were addressed. Although most studies did not directly quantify carryover effects, the use of counterbalanced randomization schemes and adjustments for order and period effects in the analyses partially mitigated this risk. These inherent limitations of crossover designs will be further elaborated in the Discussion section.

The assessment results were visually presented using a traffic light plot combined with a weighted bar chart, and these were considered when interpreting the meta-analysis results. In subsequent quantitative analyses, a sensitivity analysis excluding studies with an overall “high risk” of bias was performed to test the robustness of the primary analysis results. Furthermore, when a sufficient number of studies were included, subgroup analyses based on individual bias domains (e.g., comparing effect size differences between studies using objective versus subjective outcome measurements) were planned to explore the potential impact of specific methodological characteristics on effect estimates.

### 2.7 Effect size calculation

#### 2.7.1 Selection of effect size indices

This study employed the standardized mean difference (SMD) as the effect size index, specifically utilizing Hedges’ g to correct for potential small-sample bias inherent in the SMD. Hedges’ g and its sampling variance were calculated based on the post-intervention mean, standard deviation, and sample size of each comparison, using corresponding effect size calculation methods depending on whether the study design was a parallel randomized trial or a crossover trial.

For outcome indicators with inconsistent effect directions, direction standardization was performed before pooling the effect sizes. Specifically, when a lower score on a certain outcome indicator represented a better outcome (e.g., total PSQI score, sleep onset latency, wake after sleep onset, negative mood dimensions of the POMS, DASS-21 score), the effect direction was reversed by multiplying the calculated Hedges’ g value by −1. This ensured that a positive value uniformly represented an improvement effect of the experimental group relative to the control group. The standardized effect sizes are interpreted as follows: in direct comparisons of exercise times, a positive value consistently indicates that the earlier time point in the comparison yielded a superior effect compared to the later time point (e.g., in a Morning vs. Evening comparison, a positive value indicates Morning is superior to Evening; in Afternoon vs. Evening, positive means Afternoon is superior; in Morning vs. Afternoon, positive means Morning is superior). In comparisons between exercise and control, a positive value indicates that the exercise intervention yielded a superior effect relative to the non-exercise control.

#### 2.7.2 Effect size calculation for different study designs

For parallel randomized controlled trials, Hedges’ g was calculated based on the post-intervention means, standard deviations, and sample sizes of the independent groups. For randomized crossover trials, because the same participants underwent repeated measurements under different intervention conditions, an SMD suitable for paired/repeated measures designs was employed, and within-participant correlation was incorporated into the calculation of sampling variance. Specifically, a paired effect size standardized using the pooled raw standard deviations of the two conditions was used, followed by Hedges’ small-sample correction. Since most primary studies did not report the between-condition correlation coefficient, the primary analysis assumed a paired correlation coefficient of *r* = 0.50. Further sensitivity analyses were conducted by setting r to 0.30 and 0.70 to test the impact of this assumption on effect estimation.

#### 2.7.3 Handling multiple effect sizes contributed by a single study

According to the predefined extraction rules, when a single study reported multiple eligible effect sizes–for instance, when a study included multiple outcome indicators (e.g., simultaneously reporting total PSQI score, sleep efficiency, and sleep onset latency), multiple timing comparisons (e.g., Morning vs. Evening, Afternoon vs. Evening), multiple intervention conditions (e.g., different exercise intensities compared against the same control group), or the simultaneous use of subjective and objective measurement tools for the same outcome indicator–all eligible effect sizes were extracted and assigned a unique effect size identifier (Effect_Size_ID).

Statistical dependence may exist among these multiple effect sizes originating from the same study, particularly when they share an experimental or control group, or stem from repeated measurements of the same participants. In addition to employing a three-level random-effects structure nesting Effect_Size_ID within Study_ID, this study further constructed an off-diagonal sampling variance-covariance matrix (V matrix) to represent the sampling correlations generated by shared study groups, shared control groups, and multiple related effect sizes within the same study. Concurrently, CR2 cluster-robust variance estimation with small-sample correction, clustered by Study_ID, was implemented as a robustness check for the main model inferences.

#### 2.7.4 Handling non-standard data formats

For studies where the data reporting format did not fully meet the requirements for SMD calculation, the following procedures were applied:

For studies reporting outcome data as standard errors (SEM) rather than standard deviations (SD), the SEM was converted to SD using the formula SD = SEM ×  N.

For studies reporting outcome data solely as medians, lower quartiles, and upper quartiles, the mean and SD were estimated using the methods recommended by Wan et al. (2014); for studies reporting only medians and ranges, estimation was performed using the methods recommended by Hozo et al. (2005).

All means and SDs estimated from medians, quartiles, or ranges were explicitly.

### 2.8 Statistical analysis

All statistical analyses were performed in the R statistical software environment (R Core Team, 2024). The primary meta-analyses were conducted using the rma.mv() function from the metafor package, data wrangling and auxiliary analyses primarily utilized dplyr, cluster-robust inferences were performed using clubSandwich, and matrix operations and necessary numerical processing were executed using the Matrix package (Viechtbauer, 2010; Wickham et al., 2019; R Core Team, 2024). Three-level meta-analyses estimated model parameters and variance components using restricted maximum likelihood (REML). Unless otherwise specified, all statistical tests were two-tailed with the significance level set at alpha = 0.05.

#### 2.8.1 Three-level meta-analysis

The data in this study exhibit clear hierarchical structures and statistical dependencies. A single study could contribute multiple effect sizes, including multiple sleep parameters, multiple mood dimensions, comparisons across different exercise timeframes, and multiple intervention conditions. Furthermore, some effect sizes shared a common experimental or control group, or originated from repeated measurements of the same participants. Therefore, treating effect sizes as mutually independent could underestimate sampling uncertainty. To retain as much information provided by the primary studies as possible while addressing the dependency among multiple effect sizes within the same study, a three-level meta-analysis was employed. Additionally, an off-diagonal sampling variance-covariance matrix (V matrix) was constructed to represent known or assumed sampling correlations.

The sources of variance in the three-level model were defined as follows: (1) Level 1 represents the sampling error of effect sizes; (2) Level 2 represents the within-study variance among different true effect sizes within the same study; and (3) Level 3 represents the between-study variance of true effect sizes across different studies. The random effects structure nested the effect size identifier (Effect_Size_ID) within the study identifier (Study_ID), specified as:


random=∼1|Study_ID/Effect_Size_ID

Primary models used the full sampling variance-covariance matrix V as input, rather than relying solely on the independent sampling variance of each effect size. For parallel randomized trials, covariances resulting from shared study groups were represented using the experimental and control group identifiers and respective sample sizes of each comparison. For multiple outcomes measured simultaneously within the same participant sample, in the absence of original correlation coefficients, a working sampling-error correlation coefficient of rho = 0.50 was assumed in the primary analysis. For randomized crossover trials, the sampling variance of each effect size was initially calculated based on a paired correlation coefficient of *r* = 0.50; when a single subgroup of participants contributed multiple effect sizes, the off-diagonal sampling correlation between these effect sizes was likewise approximated using rho = 0.50. Independent participant subgroups from the same study were coded separately without artificially imputing sampling covariance. The final model specification was:


rma.mv(yi,V=V,random=∼1|Study_ID/Effect_Size_ID,

$$\mathrm{method}=``{\mathrm{REML}}^{\prime \prime })$$

In the final implementation, covariances for parallel trials were constructed via shared group identifiers and sample sizes, while for crossover trials, within-study off-diagonal covariances were built upon the foundation of paired effect size variances.

To address distinct research questions, all effect sizes were divided into two non-overlapping primary datasets. Model 1 (Exercise vs. Control) included only comparisons between exercise at a specific time of day and non-exercise controls, serving to estimate the overall effect of exercise intervention relative to no exercise. Model 2 (Time vs. Time) included only direct comparisons between different exercise timing windows, aiming to evaluate the intrinsic differences attributable to exercise timing *per se*. Sleep quality and mood states were analyzed separately. Furthermore, data from Model 1 and Model 2 were combined to form Model 3, in which Comparison Type (Exercise vs. Control vs. Time vs. Time) was incorporated as a categorical moderator for sleep outcomes to test whether systematic differences existed between the effects estimated by the two comparison types. Model 3 utilized the same off-diagonal V matrix and three-level random-effects structure as the primary models.

Considering that conventional model inference may be affected by a small number of clusters when independent studies are limited, cluster-robust inference with CR2 small-sample correction clustered by Study_ID was implemented, using the Satterthwaite approximation to estimate denominator degrees of freedom as a robustness check for the main REML model results. When a model contained fewer than 3 independent study clusters, CR2 inference was not conducted, and the reason for non-estimability was explicitly reported. CR2 results served as robustness checks and did not replace the primary pre-specified three-level REML models. Similarly, CR2 was explicitly configured in the final code as a robustness analysis clustered by Study_ID.

Heterogeneity was described using Cochran’s Q test, model-estimated variance components, and I-squared decomposition. For the random structure Study_ID/Effect_Size_ID, the first variance component corresponds to Level 3 between-study variance, while the second corresponds to Level 2 within-study variance among effect sizes. Level 2, Level 3, and overall I-squared were calculated separately to characterize the relative distribution of heterogeneity at both within- and between-study levels. I-squared decomposition was calculated using the model-estimated Level 2 and Level 3 variance components in combination with the typical sampling variance.

#### 2.8.2 Subgroup and moderator analyses

For the direct timing comparisons in Model 2, effect sizes were not categorized based on the timing label (“Morning”, “Afternoon”, or “Evening”) of a single experimental group, but were instead grouped by the actual Direct Comparison Pair for pre-specified subgroup analyses, including: Morning vs. Evening, Afternoon vs. Evening, and Morning vs. Afternoon. Effect directions were standardized such that positive values indicate that the earlier time window in the comparison pair was superior to the later time window.

Each direct comparison subgroup was estimated using its corresponding effect sizes and off-diagonal V matrix. When a comparison included at least two independent studies with sufficient effect sizes, the same three-level REML model as the primary analysis was applied; if a comparison originated from only one independent study, it was not interpreted as a pooled effect across studies, but was instead estimated using fixed-effect generalized least squares (GLS) incorporating the off-diagonal V matrix, and explicitly designated as a single-study estimate.

Additionally, Model 3 was constructed for sleep quality outcomes with Comparison Type as a moderating variable. Using Exercise vs. Control as the reference category, Time vs. Time was included as a categorical moderator in the three-level meta-regression model to evaluate effect differences between direct timing comparisons and exercise-control comparisons. This moderation model maintained the full off-diagonal V matrix, REML estimation, and Study_ID / Effect_Size_ID random-effects structure, reporting both conventional model estimates and CR2 small-sample robust inferences where estimable.

Factors such as intervention duration, measurement tools, population characteristics, exercise modality, age, and exercise dose were originally planned for exploration as potential moderators. However, because further stratification left most subcategories with an insufficient number of independent studies to yield reasonably stable multivariable or granular subgroup estimates, overly fine-grained meta-regression analyses were not performed. These variables were nevertheless described as study characteristics and taken into account in the interpretation of results and limitation analyses.

#### 2.8.3 Sensitivity analysis

To assess the sensitivity of the main conclusions to study quality, single-study influence, and correlation assumptions, the following sensitivity analyses were conducted:

First, a leave-one-study-out analysis was performed for the sleep quality analysis in Model 2. In each step, one independent study was omitted, followed by rebuilding the corresponding off-diagonal V matrix and refitting the three-level REML model to evaluate whether any single study exerted undue influence on the overall effect direction, confidence intervals, or statistical inferences.

Second, studies judged to have an overall high risk of bias according to the RoB 2 assessment were excluded, and the Model 2 sleep model was refitted to evaluate the impact of study quality on the direct exercise timing comparisons. After excluding high-risk studies, the same V matrix construction method and three-level REML model were retained.

Third, sensitivity analyses were conducted on the two working correlation coefficients required for constructing effect sizes and the V matrix. The primary analysis assumed a within-participant paired correlation coefficient of *r* = 0.50 for crossover trials, which was varied to *r* = 0.30 and *r* = 0.70 while keeping the sampling error correlation coefficient constant at rho = 0.50. Subsequently, maintaining *r* = 0.50, the working sampling error correlation coefficient between related effect sizes within the same study was adjusted to rho = 0.30 and rho = 0.70. These scenarios were applied to both sleep and mood outcomes in Model 1 and Model 2 to test whether findings were dependent on specific correlation assumptions.

Finally, data converted from medians and IQRs to means and SDs were reviewed, and their impact was evaluated by excluding these effect sizes where data permitted. If only one independent study remained after exclusion, pooled cross-study effects were not recalculated, and only the number of remaining effect sizes and independent studies was reported. Processing of converted data in the final dataset followed this principle.

Other originally planned sensitivity analyses, such as further stratifying data by measurement device, attrition rate, or study design type, were considered only if a sufficient number of independent studies remained after exclusion. When data sparsity prevented the models from obtaining meaningful cross-study estimates, further quantitative synthesis was omitted to avoid over-interpretation based on very few studies.

#### 2.8.4 Publication bias

Given the limited number of included studies and the presence of multiple correlated effect sizes from single studies, the evaluation of publication bias and small-sample effects was treated as exploratory. Funnel plots were generated separately for sleep quality and mood outcomes in Model 2 to describe the distribution of effect sizes against their sampling precision.

Because traditional Egger’s tests assume mutual independence among effect sizes—a condition not fully met by our data structure—a multilevel Egger-type regression test was conducted for exploratory purposes (R Core Team, 2024). Specifically, within the same three-level framework as the primary model, the standard error of effect sizes sqrt(vi) was included as a fixed-effect covariate while maintaining the full off-diagonal sampling variance-covariance matrix V, Study_ID/Effect_Size_ID random-effects structure, and REML estimation:


rma.mv(yi,V=V,mods=∼sqrt(vi),random=∼1|Study_ID/

$$\mathrm{Effect}\_\mathrm{Size}\_\mathrm{ID},\mathrm{method}=``{\mathrm{REML}}^{\prime \prime })$$

The regression coefficient of sqrt(vi) was examined to explore the presence of small-sample effects related to effect size precision. Funnel plots served solely for descriptive visualization, with formal statistical interpretation based on the multilevel regression results.

Considering that conventional funnel plot asymmetry tests typically require at least approximately 10 independent studies to achieve adequate statistical power, and the number of independent studies per outcome in this study was relatively small, results of the multilevel Egger-type regression were interpreted purely as exploratory evidence regardless of whether conventional statistical significance was reached, and were not used alone to confirm or rule out publication bias. Trim-and-fill procedures and fail-safe numbers were not applied, as their underlying assumption of effect size independence cannot be directly generalized to the multilevel dependent data structure of this study.

## 3 Results

### 3.1 Study selection

A total of 4,921 literature records were initially retrieved through systematic database searches. The specific search results from each database were as follows: Web of Science (Core Collection) 1,506, PubMed 337, Embase 756, Cochrane Library (CENTRAL) 298, APA PsycInfo and SPORTDiscus (EBSCOhost) combined 1,116, China National Knowledge Infrastructure (CNKI) 527, and Wanfang Data Knowledge Service Platform 381. All retrieved literature records were imported into EndNote X9 reference management software. Using automated deduplication combined with manual item-by-item comparison, a total of 1,863 duplicate records were removed, leaving 3,058 independent records for title and abstract screening.

Two trained reviewers independently performed title and abstract screening based on the pre-established PICOS inclusion and exclusion criteria, excluding a total of 2,811 clearly irrelevant records and retaining 247 reports for the full-text retrieval stage. Among them, full texts could not be obtained for 4 reports, leaving a total of 243 reports for full-text eligibility assessment. Following full-text review, studies not meeting the inclusion criteria were excluded due to: definitions of exercise intervention timing not meeting requirements (*n* = 86); control conditions not meeting pre-specified standards (*n* = 48); outcome measures not meeting requirements (*n* = 50); study design not meeting requirements (*n* = 29); study population not meeting requirements (*n* = 12); and duplicate publication or incomplete data (*n* = 5). Ultimately, a total of 13 studies were included. Disagreements arising during the screening process were resolved through discussion to reach a consensus, and no cases required adjudication by a third reviewer.

Ultimately, 13 randomized controlled trials meeting all inclusion criteria were included in this systematic review and meta-analysis. Following data organization, classification of comparison types, and construction of analysis datasets, a total of 81 effect sizes entered the final quantitative analysis, including 40 for sleep quality outcomes and 41 for mood state outcomes. The complete screening process and reasons for exclusion at each stage are detailed in the PRISMA 2020 flow diagram (Figure 1).

**FIGURE 1:**
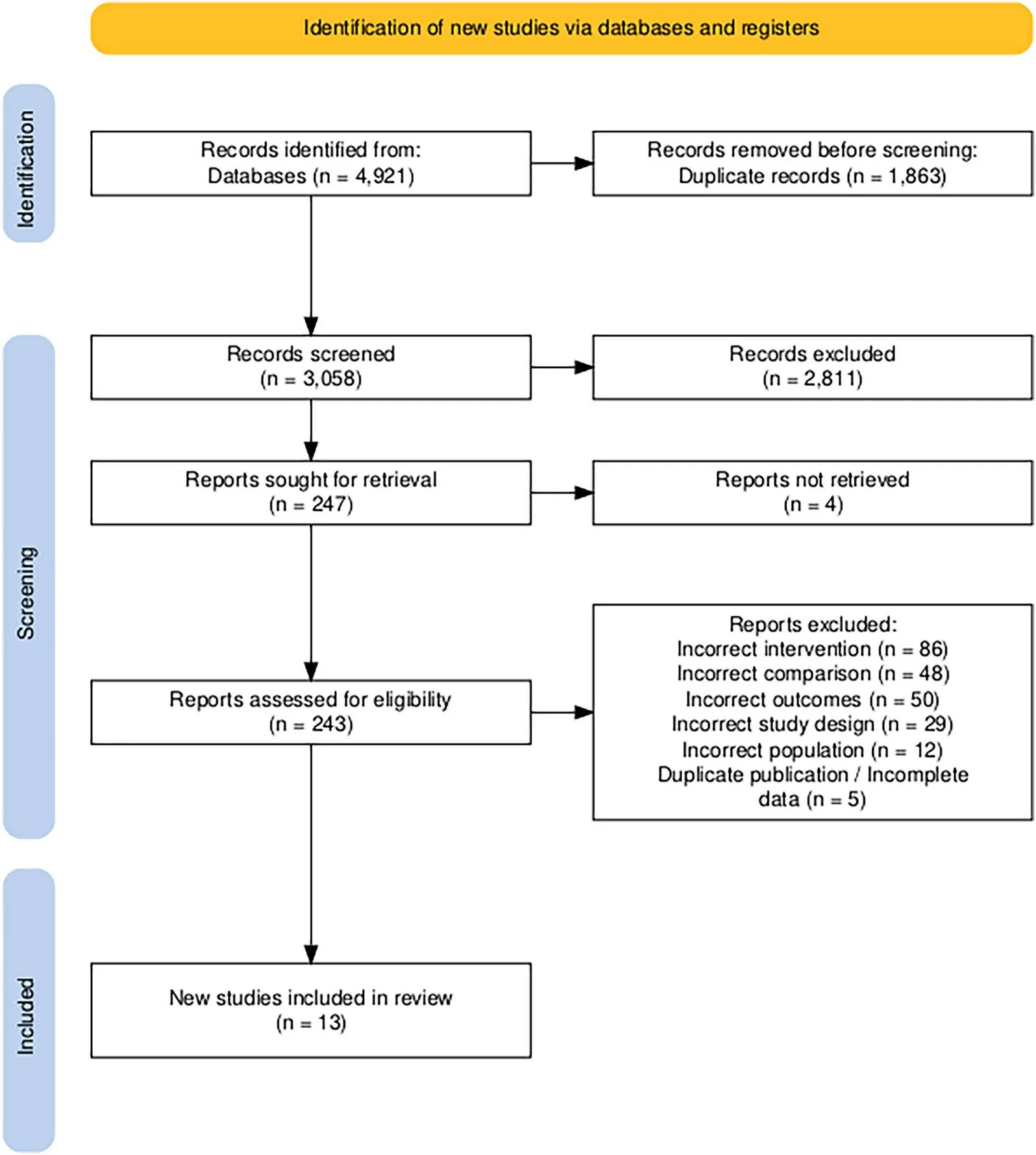
PRISMA flow diagram.

### 3.2 Study characteristics

The 13 included studies were published between 1992 and 2026 and originated from 10 countries (India, France, China, the United States, Japan, Brazil, Australia, Cyprus, Italy, and the United Kingdom) (O’Connor and Davis, 1992; Maraki et al., 2005; Passos et al., 2011; Fairbrother et al., 2014; Vitale et al., 2017; Saidi et al., 2021, 2023; Seol et al., 2021; Teo et al., 2021; Vlahoyiannis et al., 2021; Arciero et al., 2022; Shen et al., 2025; Iyer et al., 2026). The study designs included 7 parallel randomized controlled trials (RCTs) and 6 randomized crossover trials, involving a total of 442 participants finally included in the analysis, with the analytical sample size of individual studies ranging from 10 to 82 participants. The participant population exhibited high heterogeneity, covering healthy young adults, sedentary overweight/obese adults, patients with chronic insomnia, well-trained athletes, community-dwelling older adults, and individuals with borderline hypertension.

The types of exercise interventions were diverse, including yoga, running, aerobic exercise, resistance training, high-intensity interval training (HIIT), and combinations of multiple exercise modalities, with exercise intensity ranging from light to vigorous. According to the pre-established exercise timing classification criteria, the intervention conditions were primarily categorized into three time windows: morning, afternoon, and evening. For studies reporting only “AM”/“PM” or broader time windows, categorization was performed based on pre-set classification rules. Among them, 7 studies investigated regular exercise interventions lasting 4–26 weeks, while another 6 studies focused on a single bout of acute exercise. The comparison conditions of the included studies mainly comprised direct comparisons between different exercise timeframes, as well as comparisons between exercise at a specific time and no-exercise, blank, or waitlist controls.

Regarding outcome measures, sleep quality was assessed using subjective and objective multi-dimensional tools, among which the Pittsburgh Sleep Quality Index (PSQI) was the most widely used; actigraphy, polysomnography (PSG), and sleep diaries were also utilized. Mood states were measured using standardized scales such as the Profile of Mood States (POMS), Depression Anxiety Stress Scales-21 (DASS-21), Positive and Negative Affect Schedule (PANAS), and State-Anxiety Inventory (STAI-Y1). Detailed characteristics of each included study are presented in Table 1.

### 3.3 Risk of bias

The risk of bias of the included studies was assessed using the Cochrane RoB 2 tool. For parallel randomized controlled trials, the standard version of RoB 2 was used; for randomized crossover trials, the crossover-specific version was utilized. The evaluation covered five domains: bias arising from the randomization process (D1), bias due to deviations from intended interventions (D2), missing outcome data (D3), bias in measurement of the outcome (D4), and bias in selection of the reported result (D5). Based on these, an overall risk of bias judgment for each study was formed. The assessment was independently completed by two reviewers, and discrepancies were resolved through discussion and consensus. Detailed evaluation results for each study across all domains are presented in Figure 2 and Table 2.

**FIGURE 2:**
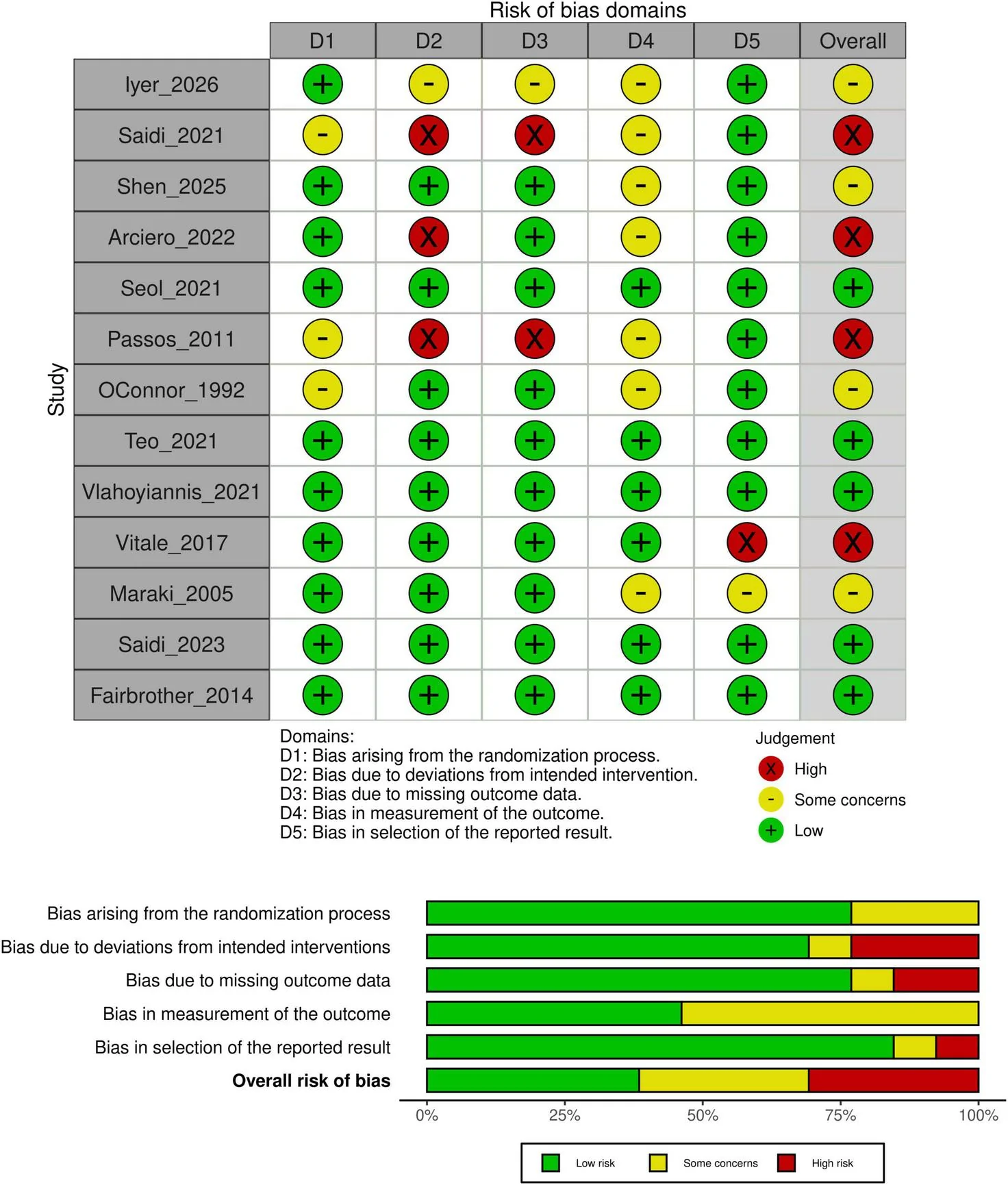
Risk of bias assessment for included studies using the RoB 2 tool. **(A)** Traffic light plot illustrating the domain-level judgments for each individual study. **(B)** Summary bar plot displaying the proportion of studies with each risk-of-bias judgment across all domains.

**TABLE 2:** Risk of bias summary.

Study	D1	D2	D3	D4	D5	Overall
Iyer_2026	Low	Some concerns	Some concerns	Some concerns	Low	Some concerns
Saidi_2021	Some concerns	High	High	Some concerns	Low	High
Shen_2025	Low	Low	Low	Some concerns	Low	Some concerns
Arciero_2022	Low	High	Low	Some concerns	Low	High
Seol_2021	Low	Low	Low	Low	Low	Low
Passos_2011	Some concerns	High	High	Some concerns	Low	High
OConnor_1992	Some concerns	Low	Low	Some concerns	Low	Some concerns
Teo_2021	Low	Low	Low	Low	Low	Low
Vlahoyiannis_2021	Low	Low	Low	Low	Low	Low
Vitale_2017	Low	Low	Low	Low	High	High
Maraki_2005	Low	Low	Low	Some concerns	Some concerns	Some concerns
Saidi_2023	Low	Low	Low	Low	Low	Low
Fairbrother_2014	Low	Low	Low	Low	Low	Low

D1, Bias arising from the randomization process; D2, bias due to deviations from intended interventions; D3, bias due to missing outcome data; D4, bias in measurement of the outcome; D5, bias in selection of the reported result. Overall risk of bias was rated as “Low” when all domains were judged as low risk; “Some concerns” when at least one domain raised some concerns but no domain was judged as high risk; and “High” when one or more domains were judged as high risk of bias.

Among the 13 included studies, the overall risk of bias was rated as low risk for 5 studies (Seol et al., 2021; Teo et al., 2021; Vlahoyiannis et al., 2021; Saidi et al., 2023; Fairbrother et al., 2014), some concerns for 4 studies (Iyer et al., 2026; Shen et al., 2025; O’Connor and Davis, 1992; Maraki et al., 2005), and high risk for the remaining 4 studies (Saidi et al., 2021; Arciero et al., 2022; Vlahoyiannis et al., 2021; Vitale et al., 2017). Overall, higher risks of bias were primarily associated with deviations from intended interventions, missing outcome data, and selective reporting. Several studies were rated as high risk in domains D2 or D3, mainly related to the inadequate use of intention-to-treat analysis, higher attrition rates, or missing outcome data. Additionally, some studies were rated as having some concerns in domain D4, suggesting that subjective outcome measurements may be susceptible to expectation effects or measurement bias.

Regarding the randomization process (D1), most studies adopted explicit random allocation procedures, resulting in an overall low risk; although some studies reported using random allocation, the descriptions of random sequence generation or allocation concealment processes were inadequate, leading to a rating of some concerns. Regarding selective reporting (D5), Vitale et al., 2017 was rated as high risk and Maraki et al., 2005 as having some concerns, while no obvious high-risk selective reporting issues were identified in the remaining studies.

To further evaluate the impact of study quality on pooled effect estimations, subsequent sensitivity analyses excluded studies rated as high risk in overall bias, and compared the refitted model results with the primary analysis to assess the sensitivity of the main conclusions to differences in study quality.

### 3.4 Effects on sleep quality

All eligible comparisons were divided into two non-overlapping datasets based on the research questions: Model 1 (Exercise vs. Control), which only included comparisons between exercise at a specific time and non-exercise controls; and Model 2 (Time vs. Time), which only included direct comparisons between different exercise timings. Three-level meta-analyses were conducted for both models separately. Model 1 and Model 2 were further combined to construct Model 3, with Comparison Type included as a moderator variable to examine whether there was a systematic difference in the effects estimated by the two types of comparisons.

In Model 1, a total of 10 sleep effect sizes from 3 independent studies were included. The three-level REML model showed that exercise at a specific time was significantly associated with improvements in sleep quality compared with non-exercise controls (Hedges’ *g* = 0.86, 95% CI [0.26, 1.46], *p* = 0.005). The test for heterogeneity did not reach statistical significance [Q(9) = 16.23, *p* = 0.062]. However, because this model included only 3 independent study clusters, after applying CR2 robust variance estimation with small-sample correction clustered by Study_ID, the point estimate of the effect remained unchanged, but the confidence interval substantially widened and crossed zero (*g* = 0.86, 95% CI [−0.47, 2.19], *p* = 0.109). Therefore, while the conventional three-level model of Model 1 suggests a substantial sleep-improving effect of exercise compared with no exercise, the robustness of this finding is limited when accounting for the inferential uncertainty introduced by the small number of independent studies.

In Model 2, a total of 30 sleep effect sizes from 9 independent studies were included. The overall effect of direct comparisons between different exercise timings was small and did not reach statistical significance (Hedges’ *g* = 0.15, 95% CI [−0.08, 0.39], *p* = 0.209). Heterogeneity was significant [Q(29) = 148.00, *p* < 0.001]. CR2 robust inference yielded similar results (*g* = 0.15, 95% CI [−0.06, 0.36], *p* = 0.127), further supporting that the overall direct timing differences did not reach statistical significance. Variance decomposition of I^2^ showed that the total I^2^ was approximately 74.0%, almost all of which originated from Level 2 within-study effect size variation (I^2^ = 74.0%), whereas Level 3 between-study heterogeneity was close to zero, suggesting that the heterogeneity in Model 2 primarily stemmed from differences among various sleep indices or comparison conditions within the same study.

Further subgroup analyses according to actual direct timing comparison combinations revealed that Morning vs. Evening included 20 effect sizes from 8 studies, showing a small and non-significant effect size (*g* = 0.08, 95% CI [−0.18, 0.35], *p* = 0.524); Afternoon vs. Evening included 8 effect sizes from 2 studies, similarly showing no significant difference (*g* = 0.05, 95% CI [−0.68, 0.77], *p* = 0.902). Morning vs. Afternoon included only 2 effect sizes from a single study, and thus a fixed-effect generalized least squares estimation accounting for its sampling covariance was employed, showing no statistically significant effect either (*g* = 0.26, 95% CI [−0.15, 0.68], *p* = 0.215); because this result derived solely from a single study, it should not be interpreted as pooled cross-study evidence. Overall, current direct comparison evidence does not indicate a stable sleep quality advantage among morning, afternoon, or evening exercise. The corresponding direct timing comparison results are presented in Figure 3.

**FIGURE 3:**
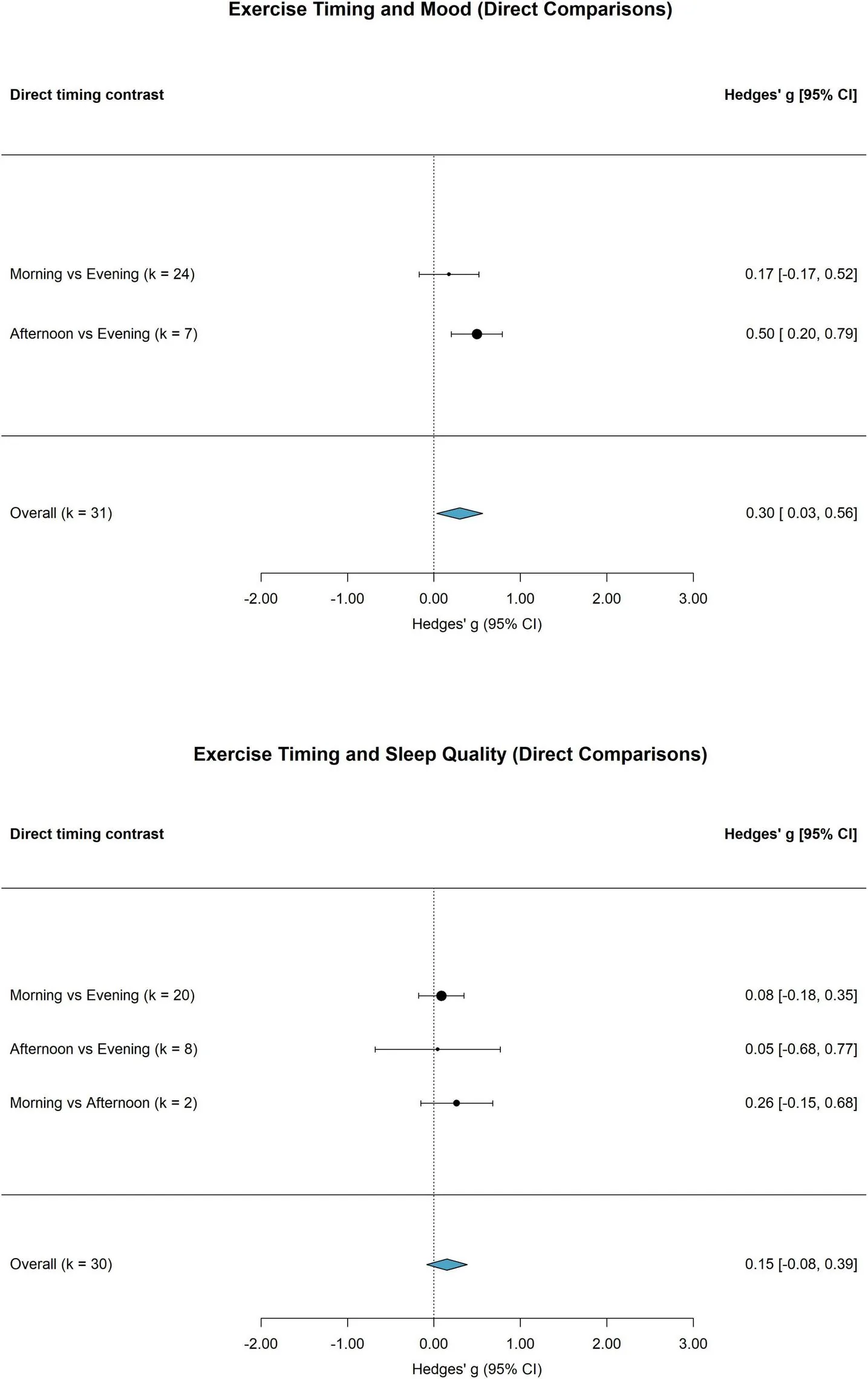
Forest plots of direct time-of-day comparisons for sleep quality and mood, based on Model 2 (Time vs. Time).

Model 3 incorporated a total of 40 sleep effect sizes and included Comparison Type as a moderator variable. In the conventional three-level REML model, the moderating effect of comparison type reached statistical significance (β = −0.65, SE = 0.24, QM(1) = 7.28, *p* = 0.007). The model-predicted effect for Exercise vs. Control was *g* = 0.80 (95% CI [0.38, 1.22]), whereas the effect for Time vs. Time was *g* = 0.15 (95% CI [−0.07, 0.37]), suggesting that the effect observed for “exercise versus no exercise” was significantly larger than that for “direct comparisons between different exercise timings.” However, after applying CR2 small-sample cluster-robust inference, this moderating effect no longer reached statistical significance (β = −0.65, 95% CI [−1.74, 0.44], *F*(1, 2.84) = 3.88, *p* = 0.149). Therefore, while the conventional model suggests that comparison type may account for part of the effect heterogeneity, this finding was not confirmed under small-sample robust inference and should be interpreted with caution.

Sensitivity analyses further supported the stability of the sleep results in Model 2. After omitting each study one by one, the pooled effect sizes ranged from *g* = 0.08 (excluding Saidi et al., 2023) to *g* = 0.19 (excluding Teo et al., 2021), and all 95% confidence.

### 3.5 Effects on mood

In Model 1, a total of 10 mood effect sizes from 2 independent studies were included. The three-level REML model showed that the effect size of exercise compared with non-exercise controls on mood was large but did not reach conventional statistical significance (Hedges’ *g* = 0.95, 95% CI [−0.02, 1.93], *p* = 0.056), with significant heterogeneity [Q(9) = 38.44, *p* < 0.001]. Because this model included only 2 independent Study_ID clusters, CR2 small-sample cluster-robust inference was not further performed; therefore, this result should be interpreted with caution in light of the limited number of independent studies.

In Model 2, a total of 31 mood effect sizes from 5 independent studies were included. The conventional three-level REML model showed that the overall effect of direct comparisons between different exercise timings reached statistical significance (Hedges’ *g* = 0.30, 95% CI [0.03, 0.56], *p* = 0.027), with a non-significant heterogeneity test [Q(30) = 34.76, *p* = 0.252]. However, after applying CR2 small-sample robust inference clustered by Study_ID, the point estimate of the effect remained unchanged, but the confidence interval widened and crossed zero (*g* = 0.30, 95% CI [−0.09, 0.68], *p* = 0.097), indicating that the statistical evidence for the overall timing effect is sensitive to small-sample robust inference.

Variance decomposition of I^2^ showed that the total I^2^ for Model 2 mood outcomes was approximately 20.2%, of which Level 3 between-study heterogeneity accounted for approximately 20.2%, whereas Level 2 within-study heterogeneity was close to zero, suggesting that the overall heterogeneity level of this model was low and primarily originated from differences between studies.

Subgroup analyses based on actual direct timing comparison combinations were further conducted. Morning vs. Evening included 24 effect sizes from 4 studies, with no significant difference found (*g* = 0.17, 95% CI [−0.17, 0.52], *p* = 0.324). Afternoon vs. Evening included 7 effect sizes from 2 studies, showing that afternoon exercise was associated with more favorable mood outcomes compared with evening exercise (*g* = 0.50, 95% CI [0.20, 0.79], *p* = 0.001). However, this comparison derived from only 2 independent studies, and thus its findings should still be interpreted with caution. There were no eligible Morning vs. Afternoon direct comparisons for mood outcomes. The main meta-analysis results for sleep quality and mood outcomes are summarized in Table 3.

**TABLE 3:** Summary of main meta-analytic results for sleep quality and mood.

Outcome/comparison type	k (effect sizes)	Studies	Hedges’ g [95% CI]	*p*
Sleep Quality				
Exercise vs. Control (Model 1)	10	3	0.86 [0.26, 1.46]	0.005
Time vs. Time (Model 2)	30	9	0.15 [−0.08, 0.39]	0.209
Morning vs. Evening	20	8	0.08 [−0.18, 0.35]	0.524
Afternoon vs. Evening	8	2	0.05 [−0.68, 0.77]	0.902
Morning vs. Afternoon	2	1	0.26 [−0.15, 0.68]^†^	0.215
Moderator analysis (Model 3)	40	10	Ex vs. Ctrl: 0.80 [0.38, 1.22]; Time vs. Time: 0.15 [−0.07, 0.37]; beta = −0.65	0.007
Sensitivity (Model 2, excl. high RoB)	21	6	0.11 [−0.16, 0.38]	0.417
Mood				
Exercise vs. Control (Model 1)	10	2	0.95 [−0.02, 1.93]	0.056
Time vs. Time (Model 2)	31	5	0.30 [0.03, 0.56]	0.027
Morning vs. Evening	24	4	0.17 [−0.17, 0.52]	0.324
Afternoon vs. Evening	7	2	0.50 [0.20, 0.79]	0.001

k, number of effect sizes; Studies, number of independent studies. Positive Hedges’ g values indicate a beneficial effect. For direct timing comparisons, positive values favor the first-named timing condition. Direct timing subgroup estimates were derived from Model 2.

† Morning vs. Afternoon for sleep quality was based on a single study and estimated using fixed-effect generalized least squares (GLS) with the corresponding sampling variance-covariance matrix; it should not be interpreted as a cross-study pooled estimate. Except for the dagger-marked single-study GLS estimate, p values presented in the table are based on the conventional three-level REML models. CR2 small-sample cluster-robust inference yielded *p* = 0.109 for Model 1 sleep, *p* = 0.127 for Model 2 sleep, *p* = 0.097 for Model 2 mood, and *p* = 0.149 for the Model 3 comparison-type moderator. CR2 was not performed for Model 1 mood because only two independent studies were available. Additional sensitivity analyses for the assumed paired and sampling correlations are reported in the text.

### 3.6 Sensitivity analyses

In addition to the leave-one-out analysis for sleep outcomes and the sensitivity analysis excluding studies with a high risk of bias reported in Section “3.4 Effects on sleep quality,” this study further examined the impact of different specifications of the crossover trial pairing correlation coefficient r and the within-study working sampling correlation coefficient rho on the primary model results. For Model 1 sleep outcomes, when r or rho were set to 0.30, 0.50, and 0.70, respectively, the effect sizes were approximately *g* = 0.84–0.88, and the conventional three-level models remained statistically significant, indicating that the point estimates were relatively stable against correlation parameter specifications. For Model 2 sleep outcomes, the effect sizes remained approximately *g* = 0.15 under different correlation specifications, and all 95% confidence intervals crossed zero, further supporting the conclusion that no stable sleep differences between exercise timings were observed in the primary analysis.

Mood outcomes were relatively more sensitive to correlation parameters. For Model 1 mood, the effect sizes ranged from approximately *g* = 0.94–0.96 across different specifications, but the *p*-values fell within a borderline range of approximately 0.050–0.064. For Model 2 mood, the effect sizes ranged from approximately *g* = 0.27–0.33 across different specifications; changing the crossover design pairing correlation coefficient r largely maintained the overall conclusions, whereas changing the working sampling correlation coefficient rho altered the statistical significance: when rho = 0.30, the effect no longer reached statistical significance (*p* = 0.074), whereas when rho = 0.70, it remained statistically significant (*p* = 0.011). Therefore, the overall effect for Model 2 mood showed some sensitivity to the within-study sampling correlation assumptions.

In addition, Maraki et al., 2005 contributed 4 effect sizes derived from medians and interquartile ranges in the Model 1 mood analysis. After excluding this study, Model 1 mood retained only 6 effect sizes from a single study (Iyer et al., 2026), making it impossible to obtain a pooled sensitivity estimate with cross-study significance. For other sensitivity analyses further stratified by measurement method, dropout status, or study design, given the limited number of available independent studies, further partitioning would have resulted in too few study clusters; therefore, no additional quantitative syntheses were performed.

### 3.7 Publication bias

Funnel plots were generated separately for sleep and mood outcomes in Model 2 (Time vs. Time) (Figure 4) to descriptively evaluate potential small-sample effects. Given the small number of independent studies, the funnel plots were presented for auxiliary illustration only. Using the same off-diagonal sampling variance-covariance matrix and three-level random-effects structure as in the primary analysis, an exploratory Egger-type regression was further conducted by including the sampling standard error of the effect sizes [sqrt(vi)] as a fixed-effect covariate.

**FIGURE 4:**
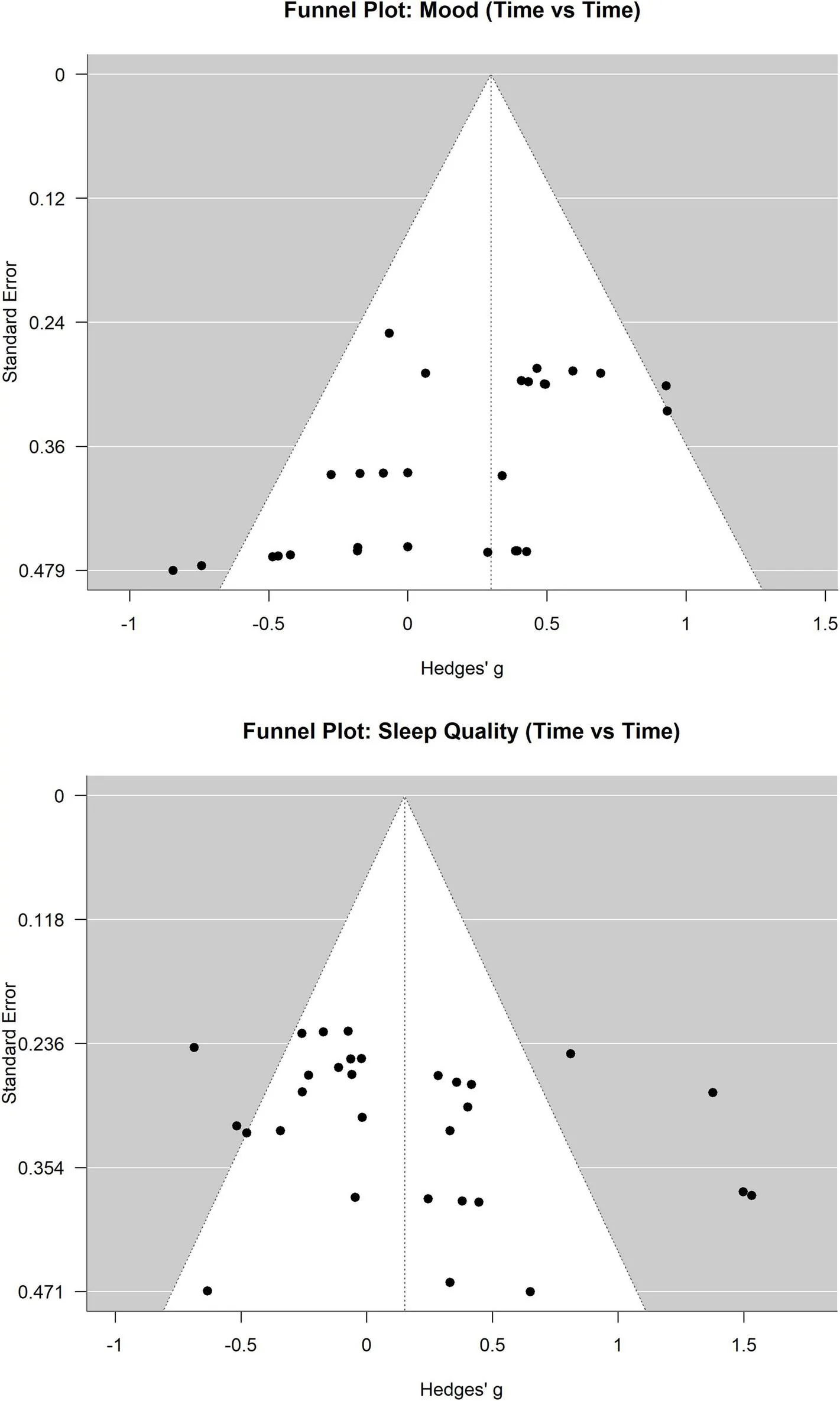
Funnel plots for sleep quality and mood based on Model 2 (Time vs. Time), presented as descriptive assessments of potential small-study effects.

For sleep outcomes, the regression coefficient for sqrt(vi) was beta = 3.12 (SE = 1.76, *p* = 0.076), which did not reach statistical significance, with a model intercept of −0.79 (*p* = 0.144). For mood outcomes, the regression coefficient for sqrt(vi) was beta = −2.28 (SE = 1.44, *p* = 0.113), which also did not reach statistical significance, with a model intercept of 1.08 (*p* = 0.025).

Because the number of independent studies included in the above analyses was only 9 (for sleep) and 5 (for mood), both of which are below the typical requirement for funnel plot asymmetry tests, the Egger-type regression results served solely as an exploratory analysis and should not be used to confirm or rule out publication bias. Overall, the available data did not provide clear statistical evidence of small-sample effects, but due to the limited number of studies, the possibility of publication bias could not be reliably determined.

## 4 Discussion

### 4.1 Summary of main findings

This study employed a three-level meta-analysis framework to systematically integrate the effects of acute and regular exercise at different times of day on sleep quality and mood states, distinguishing between two different research questions: Exercise vs. Control and Time vs. Time. Overall, current evidence increasingly supports the notion that “whether to exercise” may be more important than “specifically when to exercise,” while evidence regarding the optimal timing of exercise remains relatively limited.

Regarding sleep outcomes, the Exercise vs. Control model showed that exercise had a large favorable effect compared with non-exercise controls, with the conventional three-level model reaching statistical significance; however, the confidence interval crossed zero after CR2 robust inference, suggesting that this result remains limited by the small number of independent studies. In contrast, the Time vs. Time model found no significant overall differences between different exercise timings, and this conclusion remained stable across CR2 robust inference, leave-one-out analysis, correlation coefficient sensitivity analyses, and after excluding studies with a high risk of bias. Neither Morning vs. Evening nor Afternoon vs. Evening reached statistical significance, and Morning vs. Afternoon was based on only a single study. Therefore, current evidence is still insufficient to support a stable sleep advantage for morning, afternoon, or evening exercise.

Model 3 showed that in the conventional model, the effect of Exercise vs. Control was significantly larger than that of Time vs. Time, suggesting that the effect of exercise itself may outweigh the additional effect of exercise timing. However, this moderating effect was no longer significant after CR2 robust inference and should therefore be interpreted with caution.

Regarding mood outcomes, the effect size of the Exercise vs. Control model was large but only approached statistical significance. The Time vs. Time model reached significance in the conventional analysis, but was no longer significant after CR2 robust inference, and the result showed some sensitivity to the specification of within-study sampling correlation coefficients. In specific comparisons, Morning vs. Evening found no significant difference, whereas Afternoon vs. Evening showed a more favorable mood effect; however, this result derived from only 2 independent studies and is thus insufficient to form definitive conclusions.

Overall, this study found no stable evidence supporting a single “optimal exercise timing.” For sleep, exercise itself may be more important than exercise timing; for mood, although there are some signals of timing effects, their robustness remains limited. At this stage, it is more appropriate to view exercise timing as an additional factor that may influence health effects rather than a universally applicable decisive factor.

### 4.2 Comparison with previous literature

Previous research conclusions regarding exercise timing and sleep have been inconsistent. Some studies suggest that morning exercise may be beneficial for sleep (Seol et al., 2021), while others find that afternoon or evening exercise can similarly improve sleep (Passos et al., 2011; Vlahoyiannis et al., 2021; Saidi et al., 2023), and previous systematic reviews have indicated that evening moderate-to-low intensity exercise does not generally impair sleep quality (Stutz et al., 2019). Building upon this, the present study further employed direct comparisons between different exercise timings rather than judging the “optimal timing” solely based on the effect of exercise at a specific time compared with no exercise. The results showed that neither Morning vs. Evening nor Afternoon vs. Evening found stable sleep differences, indicating that current evidence is still insufficient to support a universal sleep advantage for any specific time of day.

This finding also suggests that when interpreting research on exercise timing, it is necessary to distinguish between the “effect of exercise itself” and the “additional effect of exercise timing.” In this study, the sleep effect of Exercise vs. Control was significantly larger than that of Time vs. Time, and the conventional Model 3 analysis also suggested a difference between the two types of comparisons, although this result was not confirmed after CR2 robust inference. Thus, the large improvement exhibited by a specific timing in previous studies may sometimes primarily reflect the overall benefit of exercise compared with no exercise, and cannot be directly equated with that timing being superior to other exercise timings.

Regarding mood outcomes, the pattern found in this study was not entirely consistent with that of sleep. The overall comparison of different exercise timings reached statistical significance in the conventional model, but its stability decreased in CR2 robust inference and some correlation coefficient sensitivity analyses, indicating that emotional responses may be influenced by multiple factors such as exercise intensity, duration, and specific exercise conditions (Basso and Suzuki, 2017; Chan et al., 2019). Notably, Afternoon vs. Evening showed more favorable mood effects, whereas Morning vs. Evening found no significant differences. However, because the former derived from only 2 independent studies, it is currently more suitable to be regarded as a signal worthy of further verification rather than forming a definitive timing recommendation.

Overall, the present study does not simply contradict previous literature, but rather further narrows the scope within which definitive interpretations can be made. Existing evidence supports that exercise itself may be beneficial for sleep and mood (Kredlow et al., 2015; Schuch et al., 2016), but evidence regarding “when to exercise to gain additional benefits” remains insufficient, and this timing effect may vary by outcome type.

### 4.3 Potential mechanisms

Exercise timing may affect sleep and mood through various factors such as circadian rhythms, post-exercise physiological recovery processes, and individual chronotypes; however, the results of this study indicate that these mechanisms do not form a consistent “optimal time window” across all studies. For sleep, exercise can influence core body temperature, autonomic nervous activity, and sleep homeostatic pressure, and these physiological responses interact potentially with the natural nocturnal sleep process (Dworak et al., 2007; Kräuchi, 2007; Porkka-Heiskanen and Kalinchuk, 2011; Stanley et al., 2013). Nevertheless, the lack of stable differences between different exercise timings suggests that these physiological effects may be jointly influenced more by exercise intensity, end time, distance to bedtime, population characteristics, and baseline sleep state rather than being solely determined by absolute clock time (Stutz et al., 2019; Vlahoyiannis et al., 2021).

Individual circadian preferences may further weaken the explanatory power of categorizing “morning, afternoon, and evening” by fixed clock times. Morning- and evening-type individuals differ in sleep-wake rhythms, physiological rhythms, subjective alertness, and optimal activity times (Adan et al., 2012); therefore, the same exercise time may correspond to different biological phases for different individuals. Some included studies have arranged or interpreted exercise timings based on chronotype (Vitale et al., 2017; Saidi et al., 2023), suggesting that future research may need to shift further from “absolute time” to the issue of “whether exercise time matches the individual’s circadian rhythm.”

Mood outcomes may be influenced by more complex contextual factors. In addition to circadian rhythms, post-exercise mood changes may also be modulated by factors such as exercise intensity, fatigue level, measurement time, and individual psychological state (Basso and Suzuki, 2017; Chan et al., 2019). Therefore, it is understandable that this study observed a positive signal for Afternoon vs. Evening, but the overall results were unstable in robust analyses. Compared with sleep, mood may not have a unified optimal exercise time, but rather manifest as the result of the combined action of individual characteristics and specific exercise contexts.

In addition, this study included both acute and regular exercise; however, due to an insufficient number of independent studies after further stratification, a formal stable moderation analysis of intervention duration was not performed. Therefore, it cannot yet be determined whether acute and regular exercise have different optimal timings. Future research needs to further compare different times of day under uniform conditions of exercise intensity, duration, and outcome measurement, while simultaneously considering chronotype and the time interval from exercise to bedtime, in order to more accurately identify truly clinically meaningful time effects.

### 4.4 Clinical implications

The results of this study suggest that, in sleep management, a fixed exercise time of day should not be regarded as a universally applicable priority at this stage. The Exercise vs. Control analysis showed that exercise may confer substantial sleep benefits compared with no exercise, whereas direct comparisons between different exercise timings revealed no stable differences. Therefore, from the perspective of practical exercise prescription, rather than emphasizing that one “must exercise in the morning, afternoon, or evening,” it may be more reasonable to prioritize ensuring that exercise can be implemented regularly and aligns with individual routines, lifestyle arrangements, and adherence.

This does not imply that exercise timing is entirely irrelevant, but rather that current evidence is insufficient to establish a uniform optimal time window. For individuals with sleep problems, exercise can be arranged flexibly on the basis of ensuring exercise safety and adherence, according to individual chronotypes, usual bedtime, and subjective post-exercise responses, without defaulting to the assumption that evening exercise is detrimental to sleep simply because it occurs at night. For mood outcomes, the Afternoon vs. Evening comparison showed a certain signal of an advantage for afternoon exercise, but because it is based on only a small number of independent studies, it is not yet sufficient to be translated into clear clinical recommendations.

Therefore, at present, exercise timing is more suitable as an ancillary moderating factor in individualized exercise prescriptions rather than a primary determinant of whether sleep or mood benefits are achieved. In the future, with the accumulation of more randomized trials directly comparing different exercise timings, it may be possible to further formulate refined timing recommendations tailored to different populations, chronotypes, and outcome goals.

### 4.5 Strengths and limitations

This study has several methodological strengths. First, it explicitly distinguished between two types of comparisons–Exercise vs. Control and Time vs. Time–avoiding the direct interpretation of the “effect of exercise itself” as the “advantage of a specific exercise timing.” Second, by employing a three-level meta-analysis and constructing a non-diagonal sampling variance-covariance matrix, multiple effect sizes from the same study were retained while accounting for shared study groups, repeated measures, and within-study effect size correlations; furthermore, CR2 small-sample robust inference clustered by Study_ID was adopted to validate the conclusions of the conventional models. Concurrently, this study conducted analyses according to actual direct comparison combinations of Morning vs. Evening, Afternoon vs. Evening, and Morning vs. Afternoon, and tested the stability of the results through sensitivity analyses including leave-one-out analysis, exclusion of studies with a high risk of bias, and different correlation coefficient assumptions. The study protocol was pre-registered, and dual outcomes of sleep and mood as well as evidence from both acute and regular exercise were systematically incorporated, thereby enhancing the completeness of evidence synthesis.

This study also has notable limitations. First, although a total of 13 studies and 81 effect sizes were included, the precision of statistical inference is fundamentally determined by the number of independent studies, whereas some models and direct timing comparisons included only a few studies. For example, Model 1 sleep included only 3 studies, Model 1 mood included only 2 studies, and the Afternoon vs. Evening mood comparison also derived from only 2 studies; therefore, although some effects had large point estimates or reached significance in conventional models, their stability remains limited. The fact that some results were no longer significant after CR2 analysis additionally reflects the uncertainty introduced by the limited number of studies.

Second, the included studies exhibited substantial variability in participant age, health status, exercise type, exercise intensity, intervention duration, and tools for measuring sleep and mood. Although the three-level model can account for part of the within- and between-study variance, it cannot entirely eliminate the influence of such clinical and methodological heterogeneity. Due to an insufficient number of independent studies following further stratification, this study was unable to perform robust moderation analyses for factors such as acute versus regular exercise, subjective versus objective measurements, exercise types, and population characteristics; thus, it remains unclear whether these factors alter the effect of exercise timing.

In addition, most crossover trials did not report correlation coefficients between conditions; therefore, effect size calculations and the sampling variance-covariance matrix required working assumptions regarding correlation coefficients. Although sensitivity analyses with varying levels of correlation coefficients generally supported the stability of sleep results, mood results exhibited a degree of sensitivity. Furthermore, some data required conversion from medians and interquartile ranges to means and standard deviations, and excluding these data left an insufficient number of independent studies for re-conducting cross-study pooling. Finally, the evaluations of publication bias for sleep and mood were based on only 9 and 5 independent studies, respectively, with limited statistical power; consequently, potential small-sample effects or publication bias cannot be reliably ruled out.

Overall, these limitations do not invalidate the existing findings, but rather indicate that randomized trial evidence regarding exercise timing remains relatively sparse at present. Particularly regarding conclusions as to whether “one time of day is truly superior to another,” further validation is required from randomized controlled trials with adequate sample sizes that directly compare multiple time points and fully account for individual chronotypes.

### 4.6 Future directions

Future research should further enhance the directness and interpretability of evidence regarding exercise timing. First, adequately powered multi-arm randomized controlled trials are needed to directly compare morning, afternoon, and evening exercise within the same study, rather than relying primarily on indirect inferences between exercise at a specific time and non-exercise controls. Study designs should also standardize exercise type, intensity, duration, and outcome measurement timing as much as possible to minimize the confounding effects of non-time factors on the results.

Second, future research should place greater emphasis on individual chronotypes and the matching relationship between exercise time and circadian rhythms. Classifying Morning, Afternoon, and Evening purely based on absolute clock time may fail to fully reflect the biological time experienced by different individuals. Incorporating chronotype, bedtime, the time interval from the end of exercise to sleep, and circadian rhythm-related indicators into study designs will help determine whether what truly matters is a “fixed time of day” or a “time matched to the individual’s biological clock.”

In addition, there is a need to separately accumulate evidence for acute and regular exercise, while strengthening the standardization of measurements for objective sleep parameters and mood outcomes. The current number of studies is insufficient to robustly evaluate the moderating roles of factors such as intervention duration, measurement methods, and population characteristics; therefore, future studies should utilize larger sample sizes and more consistent research designs to further identify which populations, outcomes, and exercise conditions are most likely to be influenced by exercise timing.

## 5 Conclusion

This study systematically integrated the effects of acute and regular exercise at different times of day on sleep quality and mood states using a three-level meta-analysis combined with a non-diagonal sampling variance-covariance matrix. The results indicate that compared with no exercise, exercise may have a pronounced favorable effect on sleep, but direct comparisons between different exercise timings did not demonstrate a stable sleep advantage. Current evidence is still insufficient to support any single time of day–morning, afternoon, or evening–as a universally applicable optimal time for exercise.

Regarding mood outcomes, different exercise timings showed a certain overall effect in conventional models, with Afternoon vs. Evening showing relatively favorable results; however, these findings were sensitive to robust inference and correlation assumptions, and the number of independent studies was limited. Consequently, clear timing recommendations cannot yet be established.

Overall, this study leans toward supporting the notion that “the implementation of exercise itself” may be more important than “specifically when exercise is performed,” whereas stable evidence for additional effects brought by exercise timing remains lacking. Future research requires more high-quality randomized controlled trials that directly compare multiple exercise timings, fully account for individual chronotypes, and employ standardized outcome measurements to further clarify the practical value of exercise timing in precision exercise prescriptions.

## Data Availability

The original contributions presented in this study are included in this article/Supplementary material, further inquiries can be directed to the corresponding author.
