# Supplementary material for: Time-of-day effects of acute and regular exercise on sleep quality and mood: a three-level meta-analysis
Source: Front Psychol. 2026 Sep 25;17:1933599. doi: 10.3389/fpsyg.2026.1933599 (PMC13649999; doi:10.3389/fpsyg.2026.1933599)
Supplement: Supplementary file 1 [file Data_Sheet_1.docx]

**1. PubMed**

(“exercise”[tiab] OR “physical activity”[tiab] OR “resistance training”[tiab] OR “aerobic”[tiab] OR “yoga”[tiab] OR “high-intensity interval”[tiab] OR “HIIT”[tiab] OR “structured training”[tiab])

AND

(“time of day”[tiab] OR “diurnal”[tiab] OR “morning”[tiab] OR “afternoon”[tiab] OR “evening”[tiab] OR “circadian rhythm”[tiab] OR “chronobiology”[tiab] OR “timing”[tiab])

AND

(“sleep”[tiab] OR “sleep quality”[tiab] OR “polysomnography”[tiab] OR “actigraphy”[tiab] OR “sleep efficiency”[tiab] OR “sleep onset latency”[tiab] OR “mood”[tiab] OR “affect”[tiab] OR “depression”[tiab] OR “anxiety”[tiab] OR “POMS”[tiab] OR “DASS-21”[tiab] OR “PANAS”[tiab])

AND

(“randomized controlled trial”[pt] OR “randomized”[tiab] OR “randomised”[tiab] OR “randomly”[tiab] OR “RCT”[tiab] OR “crossover”[tiab] OR “cross-over”[tiab])

**2. Web of Science**

TS=(“exercise” OR “physical activity” OR “resistance training” OR “aerobic” OR “yoga” OR “high-intensity interval” OR “HIIT” OR “structured training”)

AND

TS=(“time of day” OR “diurnal” OR “morning” OR “afternoon” OR “evening” OR “circadian rhythm” OR “chronobiology” OR “timing”)

AND

TS=(“sleep” OR “sleep quality” OR “polysomnography” OR “actigraphy” OR “sleep efficiency” OR “sleep onset latency” OR “mood” OR “affect” OR “depression” OR “anxiety” OR “POMS” OR “DASS-21” OR “PANAS”)

AND

TS=(“randomized” OR “randomised” OR “RCT” OR “crossover” OR “cross-over” OR “randomly”)

**3. Embase**

(‘exercise’:ti,ab OR ‘physical activity’:ti,ab OR ‘resistance training’:ti,ab OR ‘aerobic’:ti,ab OR ‘yoga’:ti,ab OR ‘high-intensity interval’:ti,ab OR ‘HIIT’:ti,ab OR ‘structured training’:ti,ab)

AND

(‘time of day’:ti,ab OR ‘diurnal’:ti,ab OR ‘morning’:ti,ab OR ‘afternoon’:ti,ab OR ‘evening’:ti,ab OR ‘circadian rhythm’:ti,ab OR ‘chronobiology’:ti,ab OR ‘timing’:ti,ab)

AND

(‘sleep’:ti,ab OR ‘sleep quality’:ti,ab OR ‘polysomnography’:ti,ab OR ‘actigraphy’:ti,ab OR ‘sleep efficiency’:ti,ab OR ‘sleep onset latency’:ti,ab OR ‘mood’:ti,ab OR ‘affect’:ti,ab OR ‘depression’:ti,ab OR ‘anxiety’:ti,ab OR ‘POMS’:ti,ab OR ‘DASS-21’:ti,ab OR ‘PANAS’:ti,ab)

AND

(‘randomized’:ti,ab OR ‘randomised’:ti,ab OR ‘randomly’:ti,ab OR ‘RCT’:ti,ab OR ‘crossover’:ti,ab OR ‘cross-over’:ti,ab)

**4. Cochrane Library**

(“exercise” OR “physical activity” OR “resistance training” OR “aerobic” OR “yoga” OR “high-intensity interval” OR “HIIT” OR “structured training”):ti,ab,kw

AND

(“time of day” OR “diurnal” OR “morning” OR “afternoon” OR “evening” OR “circadian rhythm” OR “chronobiology” OR “timing”):ti,ab,kw

AND

(“sleep” OR “sleep quality” OR “polysomnography” OR “actigraphy” OR “sleep efficiency” OR “sleep onset latency” OR “mood” OR “affect” OR “depression” OR “anxiety” OR “POMS” OR “DASS-21” OR “PANAS”):ti,ab,kw

AND

(“randomized” OR “randomised” OR “RCT” OR “crossover” OR “cross-over”):ti,ab,kw

**5 & 6. APA PsycInfo / SportDiscus (EBSCOhost)**

(TI(“exercise” OR “physical activity” OR “resistance training” OR “aerobic” OR “yoga” OR “high-intensity interval” OR “HIIT” OR “structured training”) OR AB(“exercise” OR “physical activity” OR “resistance training” OR “aerobic” OR “yoga” OR “high-intensity interval” OR “HIIT” OR “structured training”))

AND

(TI(“time of day” OR “diurnal” OR “morning” OR “afternoon” OR “evening” OR “circadian rhythm” OR “chronobiology” OR “timing”) OR AB(“time of day” OR “diurnal” OR “morning” OR “afternoon” OR “evening” OR “circadian rhythm” OR “chronobiology” OR “timing”))

AND

(TI(“sleep” OR “sleep quality” OR “polysomnography” OR “actigraphy” OR “sleep efficiency” OR “sleep onset latency” OR “mood” OR “affect” OR “depression” OR “anxiety” OR “POMS” OR “DASS-21” OR “PANAS”) OR AB(“sleep” OR “sleep quality” OR “polysomnography” OR “actigraphy” OR “sleep efficiency” OR “sleep onset latency” OR “mood” OR “affect” OR “depression” OR “anxiety” OR “POMS” OR “DASS-21” OR “PANAS”))

AND

(TI(“randomized” OR “randomised” OR “RCT” OR “crossover” OR “cross-over”) OR AB(“randomized” OR “randomised” OR “RCT” OR “crossover” OR “cross-over”))

**7. 中国知网 (CNKI)**

SU=(“运动” + “锻炼” + “身体活动” + “有氧” + “抗阻” + “力量训练” + “瑜伽” + “高强度间歇” + “HIIT”)

AND

SU=(“时段” + “运动时机” + “昼夜节律” + “生物钟” + “早晨” + “上午” + “下午” + “傍晚” + “晚间” + “夜间”)

AND

SU=(“睡眠” + “睡眠质量” + “匹兹堡” + “PSQI” + “多导睡眠” + “体动记录” + “睡眠效率” + “入睡潜伏期” + “情绪” + “心境状态” + “POMS” + “抑郁” + “焦虑” + “DASS-21” + “PANAS”)

AND

SU=(“随机对照” + “RCT” + “随机分组” + “交叉试验” + “交叉设计”)

**8. 万方数据 (Wanfang Data)**

主题:(“运动” OR “锻炼” OR “身体活动” OR “有氧” OR “抗阻” OR “力量训练” OR “瑜伽” OR “高强度间歇” OR “HIIT”)

AND

主题:(“时段” OR “运动时机” OR “昼夜节律” OR “生物钟” OR “早晨” OR “上午” OR “下午” OR “傍晚” OR “晚间” OR “夜间”)

AND

主题:(“睡眠” OR “睡眠质量” OR “匹兹堡” OR “PSQI” OR “多导睡眠” OR “体动记录” OR “睡眠效率” OR “入睡潜伏期” OR “情绪” OR “心境状态” OR “POMS” OR “抑郁” OR “焦虑” OR “DASS-21” OR “PANAS”)

AND

主题:(“随机对照” OR “RCT” OR “随机分组” OR “交叉试验” OR “交叉设计”)
