# Supplementary material for: Time-of-day effects of acute and regular exercise on sleep quality and mood: a three-level meta-analysis
Source: Front Psychol. 2026 Sep 25;17:1933599. doi: 10.3389/fpsyg.2026.1933599 (PMC13649999; doi:10.3389/fpsyg.2026.1933599)
Supplement: Supplementary file 2 [file Data_Sheet_3.docx]

\# =====================================================================

\# Supplementary File S3

\# Fully reproducible final R script

\#

\# Primary revisions in this version:

\#	1) Corrected Iyer 2026 classification:

\#	Model 1 = Iyer_2026_001 to _008

\#	Model 2 = Iyer_2026_009 to _012

\#	2) Model 3 is generated programmatically as Model 1 + Model 2.

\#	3) Sampling dependence is represented through a non-diagonal V matrix.

\#	- Parallel trials: shared arms are encoded with grp1/grp2 in vcalc().

\#	- Multiple concurrent outcomes: working sampling correlation rho = 0.50.

\#	- Crossover trials: paired standardized effects use r = 0.50, and

\#	off-diagonal sampling correlations are approximated with rho = 0.50.

\#	4) Direct timing subgroups are defined by comparison PAIR:

\#	Morning vs Evening, Afternoon vs Evening, Morning vs Afternoon.

\#	5) I-squared labels are corrected:

\#	sigma2[1] = between-study (Level 3)

\#	sigma2[2] = within-study (Level 2)

\#	6) Forest plots retain the metafor-native style used previously, but

\#	subgroup estimates are drawn directly and the overall estimate is

\#	added with addpoly(); no second meta-analysis is performed.

\#	7) CR2 cluster-robust inference (Study_ID clusters) is printed as a

\#	robustness analysis when estimable.

\#	8) Sensitivity analyses vary the assumed working correlations.

\#

\# Positive Hedges' g is oriented as a beneficial effect.

\# For direct timing contrasts, positive g favors the FIRST-named timing

\# category in Direct_Comparison_Pair.

\# =====================================================================

\# ---------------------------------------------------------------------

\# 0. Packages and analysis assumptions

\# ---------------------------------------------------------------------

required_packages <- c("metafor", "dplyr", "clubSandwich", "Matrix")

missing_packages <- required_packages[

!vapply(required_packages, requireNamespace, logical(1), quietly = TRUE)

]

if (length(missing_packages) > 0) {

stop(

"Please install the following packages before running this script: ",

paste(missing_packages, collapse = ", ")

)

}

library(metafor)

library(dplyr)

library(clubSandwich)

PAIRED_R_PRIMARY <- 0.50

SAMPLING_RHO_PRIMARY <- 0.50

cat("=========================================================\n")

cat("ANALYSIS ASSUMPTIONS\n")

cat("=========================================================\n")

cat(sprintf("Paired/crossover within-person correlation r = %.2f\n", PAIRED_R_PRIMARY))

cat(sprintf("Working sampling-error correlation rho = %.2f\n", SAMPLING_RHO_PRIMARY))

cat("Primary model: rma.mv with a full sampling variance-covariance matrix V.\n")

cat("Random effects: ~ 1 | Study_ID / Effect_Size_ID\n")

cat("CR2 robustness: cluster = Study_ID; Satterthwaite small-sample df.\n")

\# ---------------------------------------------------------------------

\# 1. Embedded data

\# ---------------------------------------------------------------------

\# ---- Model 1: Exercise vs. Control ----

model1_data <- "Study_ID	Effect_Size_ID	Time_of_Day_Exp	Direction	N_Exp	Mean_Exp_Post	SD_Exp_Post	N_Ctrl	Mean_Ctrl_Post	SD_Ctrl_Post	Comparison_Type	Intervention_Length	Outcome_Domain	Shared_Control_ID

Iyer_2026	Iyer_2026_001	Morning (06:00–07:00)	Lower_Better	27	5.07	4.224	31	10.45	4.186	Exercise vs. Control	4	Sleep Quality	Iyer_Control

Iyer_2026	Iyer_2026_002	Morning (06:00–07:00)	Lower_Better	27	3.48	3.21	31	13.1	7.89	Exercise vs. Control	4	Mood	Iyer_Control

Iyer_2026	Iyer_2026_003	Morning (06:00–07:00)	Lower_Better	27	4.3	3.45	31	13.81	7.23	Exercise vs. Control	4	Mood	Iyer_Control

Iyer_2026	Iyer_2026_004	Morning (06:00–07:00)	Lower_Better	27	3.78	2.98	31	14.84	7.34	Exercise vs. Control	4	Mood	Iyer_Control

Iyer_2026	Iyer_2026_005	Evening (18:00–19:00)	Lower_Better	24	4.08	3.256	31	10.45	4.186	Exercise vs. Control	4	Sleep Quality	Iyer_Control

Iyer_2026	Iyer_2026_006	Evening (18:00–19:00)	Lower_Better	24	5.83	4.56	31	13.1	7.89	Exercise vs. Control	4	Mood	Iyer_Control

Iyer_2026	Iyer_2026_007	Evening (18:00–19:00)	Lower_Better	24	6.08	4.12	31	13.81	7.23	Exercise vs. Control	4	Mood	Iyer_Control

Iyer_2026	Iyer_2026_008	Evening (18:00–19:00)	Lower_Better	24	6.5	4.67	31	14.84	7.34	Exercise vs. Control	4	Mood	Iyer_Control

Shen_2025	Shen_2025_001	Morning (06:00–08:00)	Lower_Better	20	3.2	1.34	15	4.4	1.82	Exercise vs. Control	12 weeks	Sleep Quality	Shen_Control

Shen_2025	Shen_2025_002	Evening (18:00–20:00)	Lower_Better	23	3.17	1.77	15	4.4	1.82	Exercise vs. Control	12 weeks	Sleep Quality	Shen_Control

Vlahoyiannis_2020	Vlahoyiannis_2020_001	Evening (7–8 pm)	Higher_Better	10	397.2	87.5	10	352.2	93.9	Exercise vs. Control	Acute (single session)	Sleep Quality	Vlahoyiannis_2020_Shared_CON

Vlahoyiannis_2020	Vlahoyiannis_2020_002	Evening (7–8 pm)	Higher_Better	10	86.2	8.4	10	79.2	8.1	Exercise vs. Control	Acute (single session)	Sleep Quality	Vlahoyiannis_2020_Shared_CON

Vlahoyiannis_2020	Vlahoyiannis_2020_003	Evening (7–8 pm)	Higher_Better	10	357.7	98.6	10	352.2	93.9	Exercise vs. Control	Acute (single session)	Sleep Quality	Vlahoyiannis_2020_Shared_CON

Vlahoyiannis_2020	Vlahoyiannis_2020_004	Evening (7–8 pm)	Higher_Better	10	84.6	8.4	10	79.2	8.1	Exercise vs. Control	Acute (single session)	Sleep Quality	Vlahoyiannis_2020_Shared_CON

Vlahoyiannis_2020	Vlahoyiannis_2020_005	Evening (7–8 pm)	Higher_Better	10	378.3	62.1	10	352.2	93.9	Exercise vs. Control	Acute (single session)	Sleep Quality	Vlahoyiannis_2020_Shared_CON

Vlahoyiannis_2020	Vlahoyiannis_2020_006	Evening (7–8 pm)	Higher_Better	10	84.1	7.2	10	79.2	8.1	Exercise vs. Control	Acute (single session)	Sleep Quality	Vlahoyiannis_2020_Shared_CON

Maraki_2005	Maraki_2005_002	Morning (08:15–09:15)	Higher_Better	12	29.5	9.26	12	19.5	7.41	Exercise vs. Control	Acute (single session)	Mood	Maraki_2005_Shared_MC

Maraki_2005	Maraki_2005_003	Evening (19:15–20:15)	Higher_Better	12	29.5	7.78	12	22.5	7.56	Exercise vs. Control	Acute (single session)	Mood	Maraki_2005_Shared_EC

Maraki_2005	Maraki_2005_005	Morning (08:15–09:15)	Lower_Better	12	10.0	0.74	12	10.0	0.74	Exercise vs. Control	Acute (single session)	Mood	Maraki_2005_Shared_MC

Maraki_2005	Maraki_2005_006	Evening (19:15–20:15)	Lower_Better	12	10.0	0.74	12	10.0	0.74	Exercise vs. Control	Acute (single session)	Mood	Maraki_2005_Shared_EC"

\# ---- Model 2: direct Time vs. Time comparisons ----

model2_data <- "Study_ID	Effect_Size_ID	Time_of_Day_Exp	Direction	N_Exp	Mean_Exp_Post	SD_Exp_Post	N_Ctrl	Mean_Ctrl_Post	SD_Ctrl_Post	Comparison_Type	Intervention_Length	Outcome_Domain

Iyer_2026	Iyer_2026_009	Morning (06:00–07:00)	Lower_Better	27	5.07	4.224	24	4.08	3.256	Time vs. Time	4	Sleep Quality

Iyer_2026	Iyer_2026_010	Morning (06:00–07:00)	Lower_Better	27	3.48	3.21	24	5.83	4.56	Time vs. Time	4	Mood

Iyer_2026	Iyer_2026_011	Morning (06:00–07:00)	Lower_Better	27	4.3	3.45	24	6.08	4.12	Time vs. Time	4	Mood

Iyer_2026	Iyer_2026_012	Morning (06:00–07:00)	Lower_Better	27	3.78	2.98	24	6.5	4.67	Time vs. Time	4	Mood

Saidi_2021	Saidi_2021_001	Morning (09:00)	Lower_Better	16	5.9	3.1	12	7.6	4.4	Time vs. Time	12	Sleep Quality

Saidi_2021	Saidi_2021_002	Morning (09:00)	Higher_Better	16	87.2	5.0	12	84.6	8.4	Time vs. Time	12	Sleep Quality

Saidi_2021	Saidi_2021_003	Morning (09:00)	Lower_Better	16	19.9	17.6	12	19.2	9.6	Time vs. Time	12	Sleep Quality

Saidi_2021	Saidi_2021_004	Morning (09:00)	Higher_Better	16	404.5	88.9	12	384.4	65.8	Time vs. Time	12	Sleep Quality

Shen_2025	Shen_2025_003	Morning (06:00–08:00)	Lower_Better	20	3.2	1.34	23	3.17	1.77	Time vs. Time	12 weeks	Sleep Quality

Arciero_2022	Arciero_2022_001	Morning (06:30–08:30)	Lower_Better	14	5.0	3.0	13	4.0	4.0	Time vs. Time	12 weeks	Mood

Arciero_2022	Arciero_2022_002	Morning (06:30–08:30)	Lower_Better	14	-2.0	12.0	13	-4.0	29.0	Time vs. Time	12 weeks	Mood

Arciero_2022	Arciero_2022_003	Morning (06:30–08:30)	Lower_Better	12	5.0	5.0	8	5.0	4.0	Time vs. Time	12 weeks	Mood

Arciero_2022	Arciero_2022_004	Morning (06:30–08:30)	Lower_Better	12	6.0	6.0	8	5.0	4.0	Time vs. Time	12 weeks	Mood

Arciero_2022	Arciero_2022_005	Morning (06:30–08:30)	Lower_Better	12	2.0	2.0	8	3.0	3.0	Time vs. Time	12 weeks	Mood

Arciero_2022	Arciero_2022_006	Morning (06:30–08:30)	Lower_Better	12	4.0	5.0	8	4.0	5.0	Time vs. Time	12 weeks	Mood

Arciero_2022	Arciero_2022_007	Morning (06:30–08:30)	Lower_Better	12	3.0	2.0	8	4.0	3.0	Time vs. Time	12 weeks	Mood

Arciero_2022	Arciero_2022_008	Morning (06:30–08:30)	Lower_Better	12	-3.0	20.0	8	4.0	12.0	Time vs. Time	12 weeks	Mood

Arciero_2022	Arciero_2022_009	Morning (06:30–08:30)	Higher_Better	12	21.0	8.0	8	18.0	4.0	Time vs. Time	12 weeks	Mood

Arciero_2022	Arciero_2022_010	Morning (06:30–08:30)	Higher_Better	14	20.0	4.0	13	21.0	7.0	Time vs. Time	12 weeks	Mood

Arciero_2022	Arciero_2022_011	Morning (06:30–08:30)	Lower_Better	14	2.0	2.0	13	4.0	8.0	Time vs. Time	12 weeks	Mood

Arciero_2022	Arciero_2022_012	Morning (06:30–08:30)	Lower_Better	14	3.0	3.0	13	3.0	4.0	Time vs. Time	12 weeks	Mood

Seol_2020	Seol_2020_001	Morning (waking–12:00)	Lower_Better	30	6.5	2.7	30	6.2	2.6	Time vs. Time	8 weeks	Sleep Quality

Seol_2020	Seol_2020_002	Morning (waking–12:00)	Higher_Better	29	89.2	4.4	28	89.5	5.6	Time vs. Time	8 weeks	Sleep Quality

Seol_2020	Seol_2020_003	Morning (waking–12:00)	Lower_Better	29	8.6	4.4	28	7.8	1.9	Time vs. Time	8 weeks	Sleep Quality

Seol_2020	Seol_2020_004	Morning (waking–12:00)	Higher_Better	29	416.8	53.0	28	400.9	57.3	Time vs. Time	8 weeks	Sleep Quality

Passos_2011	Passos_2011_001	Morning (10:00 ± 1h)	Lower_Better	10	10.5	6.0	9	6.7	5.4	Time vs. Time	6 months (3 sessions/week)	Sleep Quality

Passos_2011	Passos_2011_002	Morning (10:00 ± 1h)	Higher_Better	10	89.6	6.6	9	84.6	8.1	Time vs. Time	6 months (3 sessions/week)	Sleep Quality

Passos_2011	Passos_2011_003	Morning (10:00 ± 1h)	Lower_Better	10	4.8	3.8	9	1.7	4.2	Time vs. Time	6 months (3 sessions/week)	Mood

Passos_2011	Passos_2011_004	Morning (10:00 ± 1h)	Lower_Better	10	4.1	4.4	9	2.0	4.2	Time vs. Time	6 months (3 sessions/week)	Mood

Passos_2011	Passos_2011_005	Morning (10:00 ± 1h)	Lower_Better	10	4.8	3.8	9	1.4	3.9	Time vs. Time	6 months (3 sessions/week)	Mood

Passos_2011	Passos_2011_006	Morning (10:00 ± 1h)	Lower_Better	10	2.9	19.3	9	-7.2	20.4	Time vs. Time	6 months (3 sessions/week)	Mood

Passos_2011	Passos_2011_007	Morning (10:00 ± 1h)	Lower_Better	10	5.1	3.8	9	3.4	3.9	Time vs. Time	6 months (3 sessions/week)	Mood

Passos_2011	Passos_2011_008	Morning (10:00 ± 1h)	Lower_Better	10	1.7	3.8	9	2.9	4.2	Time vs. Time	6 months (3 sessions/week)	Mood

Passos_2011	Passos_2011_009	Morning (10:00 ± 1h)	Higher_Better	10	17.6	5.1	9	18.6	5.4	Time vs. Time	6 months (3 sessions/week)	Mood

Passos_2011	Passos_2011_010	Morning (10:00 ± 1h)	Higher_Better	10	6.2	2.2	9	5.5	1.8	Time vs. Time	6 months (3 sessions/week)	Sleep Quality

OConnor_1992	OConnor_1992_001	Morning (08:00)	Lower_Better	12	31.9	7.6	12	32.5	10.5	Time vs. Time	Acute (single session)	Mood

OConnor_1992	OConnor_1992_002	Morning (08:00)	Lower_Better	12	10.8	1.6	12	13.5	8.8	Time vs. Time	Acute (single session)	Mood

OConnor_1992	OConnor_1992_003	Afternoon (12:00)	Lower_Better	12	28.5	6.8	12	32.5	10.5	Time vs. Time	Acute (single session)	Mood

OConnor_1992	OConnor_1992_004	Afternoon (12:00)	Lower_Better	12	10.3	0.6	12	13.5	8.8	Time vs. Time	Acute (single session)	Mood

OConnor_1992	OConnor_1992_005	Afternoon (16:00)	Lower_Better	12	28.2	5.3	12	32.5	10.5	Time vs. Time	Acute (single session)	Mood

OConnor_1992	OConnor_1992_006	Afternoon (16:00)	Lower_Better	12	10.8	1.7	12	13.5	8.8	Time vs. Time	Acute (single session)	Mood

Teo_2021	Teo_2021_001	Morning (08:00–10:00)	Higher_Better	20	389.9	78.9	20	413.4	52.3	Time vs. Time	12 weeks (3 sessions/week)	Sleep Quality

Teo_2021	Teo_2021_002	Morning (08:00–10:00)	Higher_Better	20	83.6	6.9	20	86.6	5.3	Time vs. Time	12 weeks (3 sessions/week)	Sleep Quality

Teo_2021	Teo_2021_003	Morning (08:00–10:00)	Lower_Better	20	3.4	1.84	20	4.2	2.8	Time vs. Time	12 weeks (3 sessions/week)	Sleep Quality

Vitale_2016	Vitale_2016_001	Morning (08:00-09:00)	Higher_Better	12	88.5	5.5	12	78.3	7.4	Time vs. Time	Acute (single session)	Sleep Quality

Vitale_2016	Vitale_2016_002	Morning (08:00-09:00)	Higher_Better	11	83.0	6.0	11	86.0	5.0	Time vs. Time	Acute (single session)	Sleep Quality

Saidi_2023	Saidi_2023_001	Afternoon (13:00-15:00)	Higher_Better	12	89.6	6.9	12	86.4	8.3	Time vs. Time	Acute (single bout)	Sleep Quality

Saidi_2023	Saidi_2023_002	Afternoon (13:00-15:00)	Lower_Better	12	10.5	6.6	12	22.5	8.3	Time vs. Time	Acute (single bout)	Sleep Quality

Saidi_2023	Saidi_2023_003	Afternoon (13:00-15:00)	Lower_Better	12	34.2	5.5	12	43.8	12.8	Time vs. Time	Acute (single bout)	Mood

Saidi_2023	Saidi_2023_004	Afternoon (13:00-15:00)	Higher_Better	14	88.3	5.6	14	85.7	6.4	Time vs. Time	Acute (single bout)	Sleep Quality

Saidi_2023	Saidi_2023_005	Afternoon (13:00-15:00)	Lower_Better	14	34.0	7.85	14	40.9	6.4	Time vs. Time	Acute (single bout)	Mood

Saidi_2023	Saidi_2023_006	Afternoon (13:00-15:00)	Lower_Better	14	14.0	11.4	14	18.5	12.8	Time vs. Time	Acute (single bout)	Sleep Quality

Saidi_2023	Saidi_2023_007	Afternoon (13:00-15:00)	Higher_Better	16	83.4	9.2	16	83.6	8.8	Time vs. Time	Acute (single bout)	Sleep Quality

Saidi_2023	Saidi_2023_008	Afternoon (13:00-15:00)	Lower_Better	16	33.8	7.0	16	33.1	12.5	Time vs. Time	Acute (single bout)	Mood

Saidi_2023	Saidi_2023_009	Afternoon (13:00-15:00)	Lower_Better	16	18.2	11.7	16	17.4	12.6	Time vs. Time	Acute (single bout)	Sleep Quality

Fairbrother_2014	Fairbrother_2014_001	7:00 AM	Lower_Better	20	11.2	10.73	20	32.8	18.78	Time vs. Time	Acute (single session)	Sleep Quality

Fairbrother_2014	Fairbrother_2014_002	7:00 AM	Lower_Better	20	11.2	10.73	20	21.3	13.42	Time vs. Time	Acute (single session)	Sleep Quality

Fairbrother_2014	Fairbrother_2014_003	1:00 PM	Lower_Better	20	32.8	18.78	20	21.3	13.42	Time vs. Time	Acute (single session)	Sleep Quality

Fairbrother_2014	Fairbrother_2014_004	7:00 AM	Higher_Better	20	429.4	54.53	20	439.3	57.66	Time vs. Time	Acute (single session)	Sleep Quality

Fairbrother_2014	Fairbrother_2014_005	7:00 AM	Higher_Better	20	429.4	54.53	20	443.4	50.96	Time vs. Time	Acute (single session)	Sleep Quality

Fairbrother_2014	Fairbrother_2014_006	1:00 PM	Higher_Better	20	439.3	57.66	20	443.4	50.96	Time vs. Time	Acute (single session)	Sleep Quality"

\# ---------------------------------------------------------------------

\# 2. Read embedded data

\# ---------------------------------------------------------------------

dat1 <- read.delim(

text = model1_data,

header = TRUE,

sep = "\t",

quote = "",

na.strings = "NA",

stringsAsFactors = FALSE,

check.names = FALSE

)

dat2 <- read.delim(

text = model2_data,

header = TRUE,

sep = "\t",

quote = "",

na.strings = "NA",

stringsAsFactors = FALSE,

check.names = FALSE

)

id_num <- function(x) {

as.integer(sub(".*_(\\d+)$", "\\1", x))

}

\# ---------------------------------------------------------------------

\# 3. Explicit design/dependence metadata

\# ---------------------------------------------------------------------

add_model1_metadata <- function(d) {

num <- id_num(d$Effect_Size_ID)

d %>%

mutate(

Source_Model = "Model 1",

Direct_Comparison_Pair = NA_character_,

Design_Type = ifelse(

Study_ID %in% c("Vlahoyiannis_2020", "Maraki_2005"),

"Crossover",

"Parallel"

),

Sampling_Subgroup_ID = case_when(

Study_ID == "Iyer_2026" ~ "Iyer_All",

Study_ID == "Shen_2025" ~ "Shen_All",

Study_ID == "Vlahoyiannis_2020" ~ "Vlahoyiannis_All",

Study_ID == "Maraki_2005" ~ "Maraki_All",

TRUE ~ paste0(Study_ID, "_All")

),

\# Arm identifiers are used for parallel trials by vcalc().

\# For crossover rows they are retained for auditability, but

\# the crossover sampling blocks are constructed separately.

Arm1_ID = case_when(

Study_ID == "Iyer_2026" & num %in% 1:4 ~ "Iyer_Morning",

Study_ID == "Iyer_2026" & num %in% 5:8 ~ "Iyer_Evening",

Effect_Size_ID == "Shen_2025_001" ~ "Shen_Morning",

Effect_Size_ID == "Shen_2025_002" ~ "Shen_Evening",

Study_ID == "Vlahoyiannis_2020" & num %in% c(1, 2) ~ "Vlahoyiannis_SIT",

Study_ID == "Vlahoyiannis_2020" & num %in% c(3, 4) ~ "Vlahoyiannis_MICT",

Study_ID == "Vlahoyiannis_2020" & num %in% c(5, 6) ~ "Vlahoyiannis_HIIT",

Study_ID == "Maraki_2005" & num %in% c(2, 5) ~ "Maraki_Morning_Exercise",

Study_ID == "Maraki_2005" & num %in% c(3, 6) ~ "Maraki_Evening_Exercise",

TRUE ~ paste0(Study_ID, "_Experimental")

),

Arm2_ID = case_when(

Study_ID == "Iyer_2026" ~ "Iyer_Control",

Study_ID == "Shen_2025" ~ "Shen_Control",

Study_ID == "Vlahoyiannis_2020" ~ "Vlahoyiannis_CON",

Study_ID == "Maraki_2005" & num %in% c(2, 5) ~ "Maraki_Morning_Control",

Study_ID == "Maraki_2005" & num %in% c(3, 6) ~ "Maraki_Evening_Control",

TRUE ~ paste0(Study_ID, "_Control")

),

\# Same outcome labels are intentionally shared for the Iyer and Shen

\# multi-arm contrasts so vcalc() can use the arm-overlap structure

\# without imposing an additional rho for the same measured outcome.

Outcome_Obs_ID = case_when(

Study_ID == "Iyer_2026" & num %in% c(1, 5) ~ "Iyer_PSQI",

Study_ID == "Iyer_2026" & num %in% c(2, 6) ~ "Iyer_Depression",

Study_ID == "Iyer_2026" & num %in% c(3, 7) ~ "Iyer_Anxiety",

Study_ID == "Iyer_2026" & num %in% c(4, 8) ~ "Iyer_Stress",

Study_ID == "Shen_2025" ~ "Shen_PSQI",

TRUE ~ Effect_Size_ID

)

)

}

add_model2_metadata <- function(d) {

num <- id_num(d$Effect_Size_ID)

d %>%

mutate(

Source_Model = "Model 2",

Direct_Comparison_Pair = case_when(

Study_ID %in% c(

"Iyer_2026", "Saidi_2021", "Shen_2025",

"Arciero_2022", "Seol_2020", "Passos_2011",

"Teo_2021", "Vitale_2016"

) ~ "Morning_vs_Evening",

Study_ID == "OConnor_1992" & num %in% 1:2 ~ "Morning_vs_Evening",

Study_ID == "OConnor_1992" & num %in% 3:6 ~ "Afternoon_vs_Evening",

Study_ID == "Saidi_2023" ~ "Afternoon_vs_Evening",

Study_ID == "Fairbrother_2014" & num %in% c(1, 4) ~ "Morning_vs_Afternoon",

Study_ID == "Fairbrother_2014" & num %in% c(2, 5) ~ "Morning_vs_Evening",

Study_ID == "Fairbrother_2014" & num %in% c(3, 6) ~ "Afternoon_vs_Evening",

TRUE ~ NA_character_

),

Design_Type = ifelse(

Study_ID %in% c(

"OConnor_1992", "Vitale_2016",

"Saidi_2023", "Fairbrother_2014"

),

"Crossover",

"Parallel"

),

\# Independent participant subgroups within a study are separated here.

Sampling_Subgroup_ID = case_when(

Study_ID == "Iyer_2026" ~ "Iyer_All",

Study_ID == "Saidi_2021" ~ "Saidi2021_All",

Study_ID == "Shen_2025" ~ "Shen_All",

Study_ID == "Arciero_2022" & num %in% c(1, 2, 10, 11, 12) ~ "Arciero_Women",

Study_ID == "Arciero_2022" & num %in% 3:9 ~ "Arciero_Men",

Study_ID == "Seol_2020" ~ "Seol_All",

Study_ID == "Passos_2011" ~ "Passos_All",

Study_ID == "OConnor_1992" ~ "OConnor_All",

Study_ID == "Teo_2021" ~ "Teo_All",

Effect_Size_ID == "Vitale_2016_001" ~ "Vitale_MorningType",

Effect_Size_ID == "Vitale_2016_002" ~ "Vitale_EveningType",

Study_ID == "Saidi_2023" & num %in% 1:3 ~ "Saidi2023_Group12",

Study_ID == "Saidi_2023" & num %in% 4:6 ~ "Saidi2023_Group14",

Study_ID == "Saidi_2023" & num %in% 7:9 ~ "Saidi2023_Group16",

Study_ID == "Fairbrother_2014" ~ "Fairbrother_All",

TRUE ~ paste0(Study_ID, "_All")

),

\# Arm identifiers for parallel studies. Iyer and Shen deliberately

\# match Model 1 identifiers, which permits Model 3 to represent the

\# covariance among Morning-vs-Control, Evening-vs-Control, and

\# Morning-vs-Evening contrasts from the same trial.

Arm1_ID = case_when(

Study_ID == "Iyer_2026" ~ "Iyer_Morning",

Study_ID == "Shen_2025" ~ "Shen_Morning",

Design_Type == "Parallel" & Direct_Comparison_Pair == "Morning_vs_Evening" ~

paste0(Sampling_Subgroup_ID, "_Morning"),

Study_ID == "OConnor_1992" & num %in% 1:2 ~ "OConnor_Morning",

Study_ID == "OConnor_1992" & num %in% 3:4 ~ "OConnor_Afternoon12",

Study_ID == "OConnor_1992" & num %in% 5:6 ~ "OConnor_Afternoon16",

Study_ID == "Vitale_2016" ~ paste0(Sampling_Subgroup_ID, "_Morning"),

Study_ID == "Saidi_2023" ~ paste0(Sampling_Subgroup_ID, "_Afternoon"),

Study_ID == "Fairbrother_2014" & Direct_Comparison_Pair == "Morning_vs_Afternoon" ~ "Fairbrother_Morning",

Study_ID == "Fairbrother_2014" & Direct_Comparison_Pair == "Morning_vs_Evening" ~ "Fairbrother_Morning",

Study_ID == "Fairbrother_2014" & Direct_Comparison_Pair == "Afternoon_vs_Evening" ~ "Fairbrother_Afternoon",

TRUE ~ paste0(Sampling_Subgroup_ID, "_First")

),

Arm2_ID = case_when(

Study_ID == "Iyer_2026" ~ "Iyer_Evening",

Study_ID == "Shen_2025" ~ "Shen_Evening",

Design_Type == "Parallel" & Direct_Comparison_Pair == "Morning_vs_Evening" ~

paste0(Sampling_Subgroup_ID, "_Evening"),

Study_ID == "OConnor_1992" ~ "OConnor_Evening",

Study_ID == "Vitale_2016" ~ paste0(Sampling_Subgroup_ID, "_Evening"),

Study_ID == "Saidi_2023" ~ paste0(Sampling_Subgroup_ID, "_Evening"),

Study_ID == "Fairbrother_2014" & Direct_Comparison_Pair == "Morning_vs_Afternoon" ~ "Fairbrother_Afternoon",

Study_ID == "Fairbrother_2014" & Direct_Comparison_Pair == "Morning_vs_Evening" ~ "Fairbrother_Evening",

Study_ID == "Fairbrother_2014" & Direct_Comparison_Pair == "Afternoon_vs_Evening" ~ "Fairbrother_Evening",

TRUE ~ paste0(Sampling_Subgroup_ID, "_Second")

),

Outcome_Obs_ID = case_when(

Study_ID == "Iyer_2026" & num == 9 ~ "Iyer_PSQI",

Study_ID == "Iyer_2026" & num == 10 ~ "Iyer_Depression",

Study_ID == "Iyer_2026" & num == 11 ~ "Iyer_Anxiety",

Study_ID == "Iyer_2026" & num == 12 ~ "Iyer_Stress",

Study_ID == "Shen_2025" ~ "Shen_PSQI",

TRUE ~ Effect_Size_ID

)

)

}

dat1 <- add_model1_metadata(dat1)

dat2 <- add_model2_metadata(dat2)

\# Model 3 is the exact union of Models 1 and 2.

dat3 <- bind_rows(dat1, dat2)

\# ---------------------------------------------------------------------

\# 4. Automatic data-integrity checks

\# ---------------------------------------------------------------------

stopifnot(nrow(dat1) == 20L)

stopifnot(nrow(dat2) == 61L)

stopifnot(nrow(dat3) == 81L)

stopifnot(sum(dat1$Outcome_Domain == "Sleep Quality") == 10L)

stopifnot(sum(dat1$Outcome_Domain == "Mood") == 10L)

stopifnot(sum(dat2$Outcome_Domain == "Sleep Quality") == 30L)

stopifnot(sum(dat2$Outcome_Domain == "Mood") == 31L)

stopifnot(sum(dat3$Outcome_Domain == "Sleep Quality") == 40L)

stopifnot(sum(dat3$Outcome_Domain == "Mood") == 41L)

stopifnot(all(dat1$Comparison_Type == "Exercise vs. Control"))

stopifnot(all(dat2$Comparison_Type == "Time vs. Time"))

stopifnot(!anyNA(dat2$Direct_Comparison_Pair))

stopifnot(

identical(

dat1$Effect_Size_ID[dat1$Study_ID == "Iyer_2026"],

sprintf("Iyer_2026_%03d", 1:8)

)

)

stopifnot(

identical(

dat2$Effect_Size_ID[dat2$Study_ID == "Iyer_2026"],

sprintf("Iyer_2026_%03d", 9:12)

)

)

stopifnot(

dat1$Time_of_Day_Exp[dat1$Effect_Size_ID == "Iyer_2026_002"] ==

"Morning (06:00–07:00)"

)

stopifnot(!"Maraki_2005_001" %in% dat2$Effect_Size_ID)

stopifnot(!"Maraki_2005_004" %in% dat2$Effect_Size_ID)

pair_counts <- dat2 %>%

count(Outcome_Domain, Direct_Comparison_Pair)

\# Exact expected pair structure after the Iyer correction.

stopifnot(

pair_counts$n[

pair_counts$Outcome_Domain == "Sleep Quality" &

pair_counts$Direct_Comparison_Pair == "Morning_vs_Evening"

] == 20L

)

stopifnot(

pair_counts$n[

pair_counts$Outcome_Domain == "Sleep Quality" &

pair_counts$Direct_Comparison_Pair == "Afternoon_vs_Evening"

] == 8L

)

stopifnot(

pair_counts$n[

pair_counts$Outcome_Domain == "Sleep Quality" &

pair_counts$Direct_Comparison_Pair == "Morning_vs_Afternoon"

] == 2L

)

stopifnot(

pair_counts$n[

pair_counts$Outcome_Domain == "Mood" &

pair_counts$Direct_Comparison_Pair == "Morning_vs_Evening"

] == 24L

)

stopifnot(

pair_counts$n[

pair_counts$Outcome_Domain == "Mood" &

pair_counts$Direct_Comparison_Pair == "Afternoon_vs_Evening"

] == 7L

)

cat("\n=========================================================\n")

cat("DATA LOADED AND VERIFIED\n")

cat("=========================================================\n")

cat(sprintf("Model 1: %d rows (Sleep 10; Mood 10)\n", nrow(dat1)))

cat(sprintf("Model 2: %d rows (Sleep 30; Mood 31)\n", nrow(dat2)))

cat(sprintf("Model 3: %d rows (Sleep 40; Mood 41)\n", nrow(dat3)))

cat("\nModel 2 direct-comparison pair counts:\n")

print(pair_counts)

\# Export exact enriched analysis datasets.

write.csv(dat1, "Final_Model1_Data_With_Dependency_Metadata.csv", row.names = FALSE)

write.csv(dat2, "Final_Model2_Data_With_Dependency_Metadata.csv", row.names = FALSE)

write.csv(dat3, "Final_Model3_Data_With_Dependency_Metadata.csv", row.names = FALSE)

\# ---------------------------------------------------------------------

\# 5. Data cleaning

\# ---------------------------------------------------------------------

clean_data <- function(data) {

data %>%

filter(

!is.na(N_Exp),

!is.na(Mean_Exp_Post),

!is.na(SD_Exp_Post),

!is.na(N_Ctrl),

!is.na(Mean_Ctrl_Post),

!is.na(SD_Ctrl_Post)

)

}

dat1_clean <- clean_data(dat1)

dat2_clean <- clean_data(dat2)

dat3_clean <- clean_data(dat3)

stopifnot(nrow(dat1_clean) == 20L)

stopifnot(nrow(dat2_clean) == 61L)

stopifnot(nrow(dat3_clean) == 81L)

\# ---------------------------------------------------------------------

\# 6. Effect-size calculation

\#

\# Parallel trials:

\#	Hedges' g from independent-group SMD.

\#

\# Crossover trials:

\#	Hedges-corrected standardized matched-pair effect using pooled raw SD

\#	(SMCRP), with assumed within-person correlation r = 0.50.

\#

\# All signs are harmonized so positive g = beneficial.

\# ---------------------------------------------------------------------

calc_es <- function(data, domain = NULL, paired_r = PAIRED_R_PRIMARY) {

d <- data

if (!is.null(domain)) {

d <- d %>% filter(Outcome_Domain == domain)

}

if (nrow(d) == 0) return(NULL)

d <- d %>% mutate(.row_order = row_number())

d_parallel <- d %>% filter(Design_Type == "Parallel")

d_crossover <- d %>% filter(Design_Type == "Crossover")

out <- list()

if (nrow(d_parallel) > 0) {

es_parallel <- metafor::escalc(

measure = "SMD",

n1i = N_Exp,

m1i = Mean_Exp_Post,

sd1i = SD_Exp_Post,

n2i = N_Ctrl,

m2i = Mean_Ctrl_Post,

sd2i = SD_Ctrl_Post,

data = d_parallel

)

out[[length(out) + 1L]] <- as.data.frame(es_parallel)

}

if (nrow(d_crossover) > 0) {

if (any(d_crossover$N_Exp != d_crossover$N_Ctrl)) {

stop("Crossover rows require equal N_Exp and N_Ctrl in this script.")

}

es_crossover <- metafor::escalc(

measure = "SMCRP",

m1i = Mean_Exp_Post,

m2i = Mean_Ctrl_Post,

sd1i = SD_Exp_Post,

sd2i = SD_Ctrl_Post,

ri = paired_r,

ni = N_Exp,

data = d_crossover

)

out[[length(out) + 1L]] <- as.data.frame(es_crossover)

}

es <- bind_rows(out) %>%

arrange(.row_order) %>%

mutate(

yi = ifelse(

grepl("lower", tolower(Direction)),

-yi,

yi

),

Paired_r_Used = ifelse(Design_Type == "Crossover", paired_r, NA_real_)

)

es

}

\# ---------------------------------------------------------------------

\# 7. Sampling variance-covariance matrix V

\#

\# Parallel trials:

\#	vcalc() uses grp1/grp2 and group sizes to represent covariance induced

\#	by shared arms. Outcome_Obs_ID + rho represents unknown correlation

\#	among concurrently measured different outcomes.

\#

\# Crossover trials:

\#	effect-size variances already use paired r. Off-diagonal covariance

\#	among multiple estimates from the same participant subgroup is

\#	approximated with compound-symmetric working rho via vcalc().

\#

\# Independent subgroups within the same study are separated by

\# Sampling_Subgroup_ID and therefore receive zero off-diagonal covariance.

\# ---------------------------------------------------------------------

build_V <- function(es, sampling_rho = SAMPLING_RHO_PRIMARY) {

if (is.null(es) || nrow(es) == 0) return(NULL)

V <- diag(es$vi)

rownames(V) <- es$Effect_Size_ID

colnames(V) <- es$Effect_Size_ID

idx_parallel <- which(es$Design_Type == "Parallel")

idx_crossover <- which(es$Design_Type == "Crossover")

if (length(idx_parallel) > 0) {

dp <- es[idx_parallel, , drop = FALSE]

Vp <- metafor::vcalc(

vi = vi,

cluster = Study_ID,

subgroup = Sampling_Subgroup_ID,

obs = Outcome_Obs_ID,

grp1 = Arm1_ID,

grp2 = Arm2_ID,

w1 = N_Exp,

w2 = N_Ctrl,

data = dp,

rho = sampling_rho,

checkpd = TRUE,

nearpd = TRUE

)

V[idx_parallel, idx_parallel] <- as.matrix(Vp)

}

if (length(idx_crossover) > 0) {

dc <- es[idx_crossover, , drop = FALSE]

Vc <- metafor::vcalc(

vi = vi,

cluster = Study_ID,

subgroup = Sampling_Subgroup_ID,

obs = Outcome_Obs_ID,

data = dc,

rho = sampling_rho,

checkpd = TRUE,

nearpd = TRUE

)

V[idx_crossover, idx_crossover] <- as.matrix(Vc)

}

\# Numerical safeguard only. If the assembled matrix is not positive

\# definite, use the nearest positive-definite matrix while preserving

\# the diagonal. Any such adjustment is reported in the console.

eig_min <- min(eigen(V, symmetric = TRUE, only.values = TRUE)$values)

if (!is.finite(eig_min) || eig_min <= 1e-10) {

cat(sprintf(

"NOTE: V required nearPD adjustment (minimum eigenvalue = %.3e).\n",

eig_min

))

V <- as.matrix(Matrix::nearPD(V, keepDiag = TRUE)$mat)

rownames(V) <- es$Effect_Size_ID

colnames(V) <- es$Effect_Size_ID

}

V

}

\# ---------------------------------------------------------------------

\# 8. Main model fitting and CR2 robustness

\# ---------------------------------------------------------------------

fit_3level <- function(es, V, label, mods = NULL) {

if (is.null(es) || nrow(es) < 2) {

cat("\nWARNING:", label, "- insufficient data; skipped.\n")

return(NULL)

}

if (is.null(mods)) {

res <- metafor::rma.mv(

yi = yi,

V = V,

random = ~ 1 | Study_ID / Effect_Size_ID,

data = es,

method = "REML"

)

} else {

res <- metafor::rma.mv(

yi = yi,

V = V,

mods = mods,

random = ~ 1 | Study_ID / Effect_Size_ID,

data = es,

method = "REML"

)

}

cat("\n=========================================================\n")

cat(label, "\n")

cat("=========================================================\n")

print(summary(res))

res

}

run_cr2 <- function(res, es, label) {

if (is.null(res)) return(NULL)

n_clusters <- dplyr::n_distinct(es$Study_ID)

if (n_clusters < 3) {

cat(

"\n", label,

": CR2 not performed because fewer than 3 independent Study_ID clusters are available.\n",

sep = ""

)

return(NULL)

}

cr2 <- tryCatch(

metafor::robust(

res,

cluster = es$Study_ID,

clubSandwich = TRUE

),

error = function(e) {

message(label, " CR2 failed: ", e$message)

NULL

}

)

if (!is.null(cr2)) {

cat("\n", label, " - CR2 cluster-robust inference:\n", sep = "")

print(cr2)

}

cr2

}

\# ---------------------------------------------------------------------

\# 9. Model 1: Exercise vs Control

\# ---------------------------------------------------------------------

cat("\n=========================================================\n")

cat("MODEL 1: EXERCISE VS CONTROL\n")

cat("=========================================================\n")

es1_sleep <- calc_es(dat1_clean, "Sleep Quality")

es1_mood <- calc_es(dat1_clean, "Mood")

V1_sleep <- build_V(es1_sleep)

V1_mood <- build_V(es1_mood)

res1_sleep <- fit_3level(

es1_sleep, V1_sleep,

"Model 1 - Sleep Quality (Exercise vs Control)"

)

res1_mood <- fit_3level(

es1_mood, V1_mood,

"Model 1 - Mood (Exercise vs Control)"

)

res1_sleep_cr2 <- run_cr2(

res1_sleep, es1_sleep,

"Model 1 Sleep"

)

res1_mood_cr2 <- run_cr2(

res1_mood, es1_mood,

"Model 1 Mood"

)

\# ---------------------------------------------------------------------

\# 10. Model 2: direct Time vs Time comparisons

\# ---------------------------------------------------------------------

cat("\n=========================================================\n")

cat("MODEL 2: DIRECT TIME VS TIME COMPARISONS\n")

cat("=========================================================\n")

es2_sleep <- calc_es(dat2_clean, "Sleep Quality")

es2_mood <- calc_es(dat2_clean, "Mood")

V2_sleep <- build_V(es2_sleep)

V2_mood <- build_V(es2_mood)

res2_sleep <- fit_3level(

es2_sleep, V2_sleep,

"Model 2 - Sleep Quality (Time vs Time)"

)

res2_mood <- fit_3level(

es2_mood, V2_mood,

"Model 2 - Mood (Time vs Time)"

)

res2_sleep_cr2 <- run_cr2(

res2_sleep, es2_sleep,

"Model 2 Sleep"

)

res2_mood_cr2 <- run_cr2(

res2_mood, es2_mood,

"Model 2 Mood"

)

\# ---------------------------------------------------------------------

\# 11. Direct-comparison-pair subgroup analysis

\# ---------------------------------------------------------------------

pair_levels <- c(

"Morning_vs_Evening",

"Afternoon_vs_Evening",

"Morning_vs_Afternoon"

)

pretty_pair <- function(x) {

gsub("_vs_", " vs ", x, fixed = TRUE)

}

run_pair_subgroup <- function(

es,

label,

sampling_rho = SAMPLING_RHO_PRIMARY) {

if (is.null(es) || nrow(es) < 2) return(NULL)

results <- list()

for (pair in pair_levels) {

sub <- es %>% filter(Direct_Comparison_Pair == pair)

if (nrow(sub) == 0) next

Vsub <- build_V(sub, sampling_rho = sampling_rho)

n_studies <- dplyr::n_distinct(sub$Study_ID)

\# With >=2 independent studies, retain the three-level structure.

\# With only one study, use a fixed-effects GLS synthesis with the

\# non-diagonal V matrix and explicitly flag the estimate as single-study.

if (n_studies >= 2 && nrow(sub) >= 3) {

mod <- tryCatch(

metafor::rma.mv(

yi = yi,

V = Vsub,

random = ~ 1 | Study_ID / Effect_Size_ID,

data = sub,

method = "REML"

),

error = function(e) NULL

)

model_type <- "Three-level REML"

} else {

mod <- tryCatch(

metafor::rma.mv(

yi = yi,

V = Vsub,

data = sub

),

error = function(e) NULL

)

model_type <- "Fixed-effect GLS (single study)"

}

if (is.null(mod)) next

results[[length(results) + 1L]] <- data.frame(

Pair = pair,

Contrast = pretty_pair(pair),

Estimate = as.numeric(mod$b[1]),

CI_lower = as.numeric(mod$ci.lb),

CI_upper = as.numeric(mod$ci.ub),

P_value = as.numeric(mod$pval),

k = nrow(sub),

Studies = n_studies,

Model_Type = model_type,

stringsAsFactors = FALSE

)

}

if (length(results) == 0) return(NULL)

out <- bind_rows(results) %>%

mutate(

Pair = factor(Pair, levels = pair_levels)

) %>%

arrange(Pair)

cat("\n=========================================================\n")

cat(label, "\n")

cat("=========================================================\n")

print(out)

out

}

sleep_pairs <- run_pair_subgroup(

es2_sleep,

"Model 2 Sleep - direct timing contrast subgroups"

)

mood_pairs <- run_pair_subgroup(

es2_mood,

"Model 2 Mood - direct timing contrast subgroups"

)

write.csv(

sleep_pairs,

"Direct_Comparison_Pair_Subgroups_Sleep.csv",

row.names = FALSE

)

write.csv(

mood_pairs,

"Direct_Comparison_Pair_Subgroups_Mood.csv",

row.names = FALSE

)

\# ---------------------------------------------------------------------

\# 12. Forest plots: metafor-native style

\#	No re-pooling of subgroup estimates.

\# ---------------------------------------------------------------------

plot_native_forest <- function(

subgroup_df,

overall_res,

title_text,

filename) {

if (is.null(subgroup_df) ||

nrow(subgroup_df) == 0 ||

is.null(overall_res)) {

return(NULL)

}

plot_df <- subgroup_df %>%

filter(

is.finite(Estimate),

is.finite(CI_lower),

is.finite(CI_upper)

)

if (nrow(plot_df) == 0) return(NULL)

\# 由95% CI反推标准误和方差，仅用于绘图

sei_sub <- (plot_df$CI_upper - plot_df$CI_lower) / (2 * 1.96)

vi_sub <- sei_sub^2

\# 标签缩短：不再显示 studies = X，避免与森林图区重叠

slab_labels <- sprintf(

"%s (k = %d)",

plot_df$Contrast,

plot_df$k

)

n_sub <- nrow(plot_df)

\# 拉开各行距离

rows_sub <- seq(

from = n_sub + 3,

to = 4,

by = -1

)

png(

filename,

width = 3200,

height = 2400,

res = 300

)

par(

mar = c(5, 4, 4, 2) + 0.1,

family = "sans"

)

metafor::forest(

x = plot_df$Estimate,

vi = vi_sub,

slab = slab_labels,

rows = rows_sub,

\# 增加左右留白，避免左侧文字与0线重合

ylim = c(0, n_sub + 7),

xlim = c(-5.0, 5.0),

\# 真正的效应量横轴范围仍保持与旧图一致

alim = c(-2, 3),

at = c(-2, -1, 0, 1, 2, 3),

xlab = "Hedges' g (95% CI)",

\# 稍微缩小字体

cex = 1.05,

header = c(

"Direct timing contrast",

"Hedges' g [95% CI]"

),

\# 全部改成黑色，模仿原来的图

pch = 19,

col = "black",

\# CI端点样式

efac = 0.7,

\# 0效应参考线

refline = 0,

digits = 2

)

\# 总体效应与亚组之间加横线

abline(h = 2.3, lty = 1, lwd = 1)

\# 直接添加总体效应，不重新做第二次meta分析

\# 保留你旧图中比较好看的蓝色总体diamond

metafor::addpoly(

x = as.numeric(overall_res$b[1]),

ci.lb = as.numeric(overall_res$ci.lb),

ci.ub = as.numeric(overall_res$ci.ub),

row = 1,

mlab = sprintf(

"Overall (k = %d)",

nrow(overall_res$data)

),

col = "#4FA3C7",

border = "black",

cex = 1.05

)

title(

main = title_text,

line = 2.2,

font.main = 2,

cex.main = 1.35

)

dev.off()

cat(sprintf("\nForest plot saved: %s\n", filename))

}

plot_native_forest(

sleep_pairs,

res2_sleep,

"Exercise Timing and Sleep Quality (Direct Comparisons)",

"Forest_Plot_Sleep.png"

)

plot_native_forest(

mood_pairs,

res2_mood,

"Exercise Timing and Mood (Direct Comparisons)",

"Forest_Plot_Mood.png"

)

\# ---------------------------------------------------------------------

\# 13. Model 3: Comparison Type moderator - Sleep Quality

\# ---------------------------------------------------------------------

cat("\n=========================================================\n")

cat("MODEL 3: COMPARISON TYPE MODERATOR - SLEEP QUALITY\n")

cat("=========================================================\n")

es3_sleep <- calc_es(dat3_clean, "Sleep Quality")

V3_sleep <- build_V(es3_sleep)

es3_sleep$Comparison_Type <- factor(

es3_sleep$Comparison_Type,

levels = c("Exercise vs. Control", "Time vs. Time")

)

res3 <- metafor::rma.mv(

yi = yi,

V = V3_sleep,

mods = ~ Comparison_Type,

random = ~ 1 | Study_ID / Effect_Size_ID,

data = es3_sleep,

method = "REML"

)

print(summary(res3))

res3_cr2 <- run_cr2(

res3, es3_sleep,

"Model 3 Sleep moderator"

)

pred_exercise_control <- predict(res3, newmods = 0)

pred_time_time <- predict(res3, newmods = 1)

cat("\nModel 3 predicted estimates by comparison type:\n")

cat(sprintf(

"Exercise vs Control: g = %.4f [%.4f, %.4f]\n",

pred_exercise_control$pred,

pred_exercise_control$ci.lb,

pred_exercise_control$ci.ub

))

cat(sprintf(

"Time vs Time: g = %.4f [%.4f, %.4f]\n",

pred_time_time$pred,

pred_time_time$ci.lb,

pred_time_time$ci.ub

))

\# ---------------------------------------------------------------------

\# 14. Corrected I-squared decomposition

\#

\# For random = ~1 | Study_ID / Effect_Size_ID:

\#	sigma2[1] = Study_ID variance	= Level 3 / between-study

\#	sigma2[2] = nested Effect_Size variance = Level 2 / within-study

\# ---------------------------------------------------------------------

calc_i2_3level <- function(model, V) {

if (is.null(model) || length(model$sigma2) < 2) return(NULL)

sigma2_between <- model$sigma2[1]

sigma2_within <- model$sigma2[2]

typical_sampling_var <- median(diag(V), na.rm = TRUE)

total_var <- sigma2_between + sigma2_within + typical_sampling_var

data.frame(

Level = c(

"Level 2 (Within study)",

"Level 3 (Between study)",

"Total"

),

I2_Percent = c(

sigma2_within / total_var * 100,

sigma2_between / total_var * 100,

(sigma2_within + sigma2_between) / total_var * 100

)

)

}

i2_sleep <- calc_i2_3level(res2_sleep, V2_sleep)

i2_mood <- calc_i2_3level(res2_mood, V2_mood)

cat("\nModel 2 Sleep - corrected I-squared decomposition:\n")

print(i2_sleep)

cat("\nModel 2 Mood - corrected I-squared decomposition:\n")

print(i2_mood)

write.csv(i2_sleep, "I2_Decomposition_Sleep.csv", row.names = FALSE)

write.csv(i2_mood, "I2_Decomposition_Mood.csv", row.names = FALSE)

\# ---------------------------------------------------------------------

\# 15. Leave-one-study-out sensitivity analysis: Model 2 Sleep

\# ---------------------------------------------------------------------

leave_one_study_out <- function(

es,

label,

sampling_rho = SAMPLING_RHO_PRIMARY) {

studies <- sort(unique(es$Study_ID))

out <- lapply(studies, function(study_to_remove) {

d <- es %>% filter(Study_ID != study_to_remove)

Vd <- build_V(d, sampling_rho = sampling_rho)

mod <- tryCatch(

metafor::rma.mv(

yi = yi,

V = Vd,

random = ~ 1 | Study_ID / Effect_Size_ID,

data = d,

method = "REML"

),

error = function(e) NULL

)

if (is.null(mod)) {

return(data.frame(

Excluded_Study = study_to_remove,

k = nrow(d),

Studies = dplyr::n_distinct(d$Study_ID),

Estimate = NA_real_,

CI_lower = NA_real_,

CI_upper = NA_real_,

P_value = NA_real_

))

}

data.frame(

Excluded_Study = study_to_remove,

k = nrow(d),

Studies = dplyr::n_distinct(d$Study_ID),

Estimate = as.numeric(mod$b[1]),

CI_lower = as.numeric(mod$ci.lb),

CI_upper = as.numeric(mod$ci.ub),

P_value = as.numeric(mod$pval)

)

}) %>% bind_rows()

cat("\n=========================================================\n")

cat(label, "\n")

cat("=========================================================\n")

print(out)

out

}

loo_sleep <- leave_one_study_out(

es2_sleep,

"Model 2 Sleep - Leave-one-study-out analysis"

)

write.csv(

loo_sleep,

"Leave_One_Out_Sleep.csv",

row.names = FALSE

)

\# ---------------------------------------------------------------------

\# 16. High-risk-of-bias sensitivity: Model 2 Sleep

\# ---------------------------------------------------------------------

high_risk_studies <- c(

"Saidi_2021",

"Arciero_2022",

"Passos_2011",

"Vitale_2016"

)

es2_sleep_lowrob <- es2_sleep %>%

filter(!Study_ID %in% high_risk_studies)

V2_sleep_lowrob <- build_V(es2_sleep_lowrob)

cat("\n=========================================================\n")

cat("MODEL 2 SLEEP - EXCLUDING HIGH-RISK-OF-BIAS STUDIES\n")

cat("=========================================================\n")

cat(sprintf(

"Remaining: %d effect sizes from %d studies\n",

nrow(es2_sleep_lowrob),

dplyr::n_distinct(es2_sleep_lowrob$Study_ID)

))

res_sleep_lowrob <- metafor::rma.mv(

yi = yi,

V = V2_sleep_lowrob,

random = ~ 1 | Study_ID / Effect_Size_ID,

data = es2_sleep_lowrob,

method = "REML"

)

print(summary(res_sleep_lowrob))

\# ---------------------------------------------------------------------

\# 17. Working-correlation sensitivity analysis

\#

\# Scenario A: vary paired/crossover r, keep sampling rho = .50.

\# Scenario B: vary sampling rho, keep paired r = .50.

\#

\# These scenarios are run for Models 1 and 2 so the reviewer can directly

\# inspect sensitivity to the working correlation assumptions.

\# ---------------------------------------------------------------------

fit_scenario <- function(

source_data,

model_name,

domain,

paired_r,

sampling_rho,

scenario_label) {

es <- calc_es(

source_data,

domain = domain,

paired_r = paired_r

)

V <- build_V(

es,

sampling_rho = sampling_rho

)

mod <- metafor::rma.mv(

yi = yi,

V = V,

random = ~ 1 | Study_ID / Effect_Size_ID,

data = es,

method = "REML"

)

data.frame(

Scenario = scenario_label,

Model = model_name,

Outcome = domain,

Paired_r = paired_r,

Sampling_rho = sampling_rho,

k = nrow(es),

Studies = dplyr::n_distinct(es$Study_ID),

Estimate = as.numeric(mod$b[1]),

CI_lower = as.numeric(mod$ci.lb),

CI_upper = as.numeric(mod$ci.ub),

P_value = as.numeric(mod$pval),

stringsAsFactors = FALSE

)

}

sensitivity_scenarios <- bind_rows(

data.frame(

Scenario = c("Primary", "Paired r = .30", "Paired r = .70"),

Paired_r = c(.50, .30, .70),

Sampling_rho = c(.50, .50, .50)

),

data.frame(

Scenario = c("Sampling rho = .30", "Sampling rho = .70"),

Paired_r = c(.50, .50),

Sampling_rho = c(.30, .70)

)

)

analysis_sources <- list(

"Model 1" = dat1_clean,

"Model 2" = dat2_clean

)

corr_sensitivity <- bind_rows(

lapply(names(analysis_sources), function(model_name) {

source_data <- analysis_sources[[model_name]]

bind_rows(lapply(c("Sleep Quality", "Mood"), function(domain) {

bind_rows(lapply(seq_len(nrow(sensitivity_scenarios)), function(i) {

fit_scenario(

source_data = source_data,

model_name = model_name,

domain = domain,

paired_r = sensitivity_scenarios$Paired_r[i],

sampling_rho = sensitivity_scenarios$Sampling_rho[i],

scenario_label = sensitivity_scenarios$Scenario[i]

)

}))

}))

})

)

cat("\n=========================================================\n")

cat("WORKING-CORRELATION SENSITIVITY - MODELS 1 AND 2\n")

cat("=========================================================\n")

print(corr_sensitivity)

write.csv(

corr_sensitivity,

"Working_Correlation_Sensitivity_Models1_2.csv",

row.names = FALSE

)

\# ---------------------------------------------------------------------

\# 17b. Median/IQR-converted data audit

\#

\# Maraki_2005 contributes median/IQR-converted data to Model 1 Mood.

\# The two Maraki direct Time-vs-Time rows are not in Model 2.

\# Excluding Maraki from Model 1 Mood leaves only Iyer_2026 (one study),

\# so a pooled Model 1 Mood sensitivity estimate is not identifiable.

\# ---------------------------------------------------------------------

maraki_model1_rows <- dat1_clean %>%

filter(Study_ID == "Maraki_2005")

cat("\n=========================================================\n")

cat("MEDIAN/IQR-CONVERTED DATA AUDIT\n")

cat("=========================================================\n")

cat(sprintf(

"Maraki_2005 contributes %d Model 1 Mood effect sizes converted from median/IQR.\n",

nrow(maraki_model1_rows)

))

cat("Maraki_2005 direct Time-vs-Time rows are excluded from Model 2.\n")

model1_mood_no_maraki <- dat1_clean %>%

filter(

Outcome_Domain == "Mood",

Study_ID != "Maraki_2005"

)

cat(sprintf(

"After excluding Maraki_2005, Model 1 Mood contains %d effect sizes from %d study/studies; pooled sensitivity is therefore not estimated.\n",

nrow(model1_mood_no_maraki),

dplyr::n_distinct(model1_mood_no_maraki$Study_ID)

))

\# ---------------------------------------------------------------------

\# 18. Exploratory Egger-type regression and funnel plots

\#	The same non-diagonal V matrix is used in the regression.

\#	Interpret cautiously because the number of independent studies is small.

\# ---------------------------------------------------------------------

run_egger <- function(es, V, overall_model, label, filename, main_title) {

cat("\n=========================================================\n")

cat(label, "\n")

cat("=========================================================\n")

egger <- tryCatch(

metafor::rma.mv(

yi = yi,

V = V,

mods = ~ sqrt(vi),

random = ~ 1 | Study_ID / Effect_Size_ID,

data = es,

method = "REML"

),

error = function(e) {

message("Egger-type regression failed: ", e$message)

NULL

}

)

if (!is.null(egger)) {

print(summary(egger))

}

\# The funnel display is descriptive. The fitted Model 2 object is used

\# only for plotting; formal interpretation comes from the multilevel

\# regression above.

png(

filename,

width = 2400,

height = 2000,

res = 300

)

metafor::funnel(

x = es$yi,

vi = es$vi,

refline = as.numeric(overall_model$b[1]),

main = main_title,

xlab = "Hedges' g",

las = 1

)

dev.off()

cat(sprintf("Funnel plot saved: %s\n", filename))

egger

}

egger_sleep <- run_egger(

es2_sleep,

V2_sleep,

res2_sleep,

"SLEEP - EXPLORATORY EGGER-TYPE REGRESSION",

"Funnel_Plot_Sleep.png",

"Funnel Plot: Sleep Quality (Time vs Time)"

)

egger_mood <- run_egger(

es2_mood,

V2_mood,

res2_mood,

"MOOD - EXPLORATORY EGGER-TYPE REGRESSION",

"Funnel_Plot_Mood.png",

"Funnel Plot: Mood (Time vs Time)"

)

\# ---------------------------------------------------------------------

\# 19. Compact key-results table

\# ---------------------------------------------------------------------

extract_main_result <- function(model, model_name, outcome_name) {

if (is.null(model)) return(NULL)

data.frame(

Model = model_name,

Outcome = outcome_name,

k = model$k,

Studies = dplyr::n_distinct(model$data$Study_ID),

Hedges_g = as.numeric(model$b[1]),

CI_lower = as.numeric(model$ci.lb),

CI_upper = as.numeric(model$ci.ub),

p_value = as.numeric(model$pval),

Q = if (!is.null(model$QE)) as.numeric(model$QE) else NA_real_,

Q_df = if (!is.null(model$k)) model$k - length(model$b) else NA_real_,

Q_p = if (!is.null(model$QEp)) as.numeric(model$QEp) else NA_real_,

stringsAsFactors = FALSE

)

}

main_results <- bind_rows(

extract_main_result(res1_sleep, "Model 1", "Sleep Quality"),

extract_main_result(res1_mood, "Model 1", "Mood"),

extract_main_result(res2_sleep, "Model 2", "Sleep Quality"),

extract_main_result(res2_mood, "Model 2", "Mood")

)

cat("\n=========================================================\n")

cat("KEY RESULTS\n")

cat("=========================================================\n")

print(main_results)

write.csv(

main_results,

"Main_Model_Results.csv",

row.names = FALSE

)

model3_results <- data.frame(

Term = names(stats::coef(res3)),

Estimate = as.numeric(stats::coef(res3)),

SE = as.numeric(res3$se),

CI_lower = as.numeric(res3$ci.lb),

CI_upper = as.numeric(res3$ci.ub),

P_value = as.numeric(res3$pval),

stringsAsFactors = FALSE

)

write.csv(

model3_results,

"Model3_Moderator_Results.csv",

row.names = FALSE

)

\# Save the exact V matrices for audit/reproduction.

write.csv(V1_sleep, "V_Model1_Sleep.csv", row.names = TRUE)

write.csv(V1_mood, "V_Model1_Mood.csv", row.names = TRUE)

write.csv(V2_sleep, "V_Model2_Sleep.csv", row.names = TRUE)

write.csv(V2_mood, "V_Model2_Mood.csv", row.names = TRUE)

write.csv(V3_sleep, "V_Model3_Sleep.csv", row.names = TRUE)

capture.output(

sessionInfo(),

file = "Session_Info.txt"

)

cat("\n=========================================================\n")

cat("ANALYSIS COMPLETE\n")

cat("=========================================================\n")

cat("Expected embedded-data counts:\n")

cat(" Model 1 = 20 (Sleep 10; Mood 10)\n")

cat(" Model 2 = 61 (Sleep 30; Mood 31)\n")

cat(" Model 3 = 81 (Sleep 40; Mood 41)\n")

cat("\nPrimary dependency assumptions:\n")

cat(sprintf(" Paired/crossover r = %.2f\n", PAIRED_R_PRIMARY))

cat(sprintf(" Sampling-error rho = %.2f\n", SAMPLING_RHO_PRIMARY))

cat("\nKey output files include:\n")

cat(" Main_Model_Results.csv\n")

cat(" Model3_Moderator_Results.csv\n")

cat(" Direct_Comparison_Pair_Subgroups_Sleep.csv\n")

cat(" Direct_Comparison_Pair_Subgroups_Mood.csv\n")

cat(" Working_Correlation_Sensitivity_Models1_2.csv\n")

cat(" Leave_One_Out_Sleep.csv\n")

cat(" Forest_Plot_Sleep.png\n")

cat(" Forest_Plot_Mood.png\n")

cat(" Funnel_Plot_Sleep.png\n")

cat(" Funnel_Plot_Mood.png\n")

cat(" V_Model1_Sleep.csv / V_Model1_Mood.csv\n")

cat(" V_Model2_Sleep.csv / V_Model2_Mood.csv\n")

cat(" V_Model3_Sleep.csv\n")

cat(" Session_Info.txt\n")

cat("=========================================================\n")
