# Supplementary material for: Time-of-day effects of acute and regular exercise on sleep quality and mood: a three-level meta-analysis
Source: Front Psychol. 2026 Sep 25;17:1933599. doi: 10.3389/fpsyg.2026.1933599 (PMC13649999; doi:10.3389/fpsyg.2026.1933599)
Supplement: Supplementary file 3 [file Data_Sheet_4.docx]

> # =====================================================================

> # Supplementary File S4

> # Fully reproducible final R script

> #

> # Primary revisions in this version:

> #	1) Corrected Iyer 2026 classification:

> #	Model 1 = Iyer_2026_001 to _008

> #	Model 2 = Iyer_2026_009 to _012

> #	2) Model 3 is generated programmatically as Model 1 + Model 2.

> #	3) Sampling dependence is represented through a non-diagonal V matrix.

> #	- Parallel trials: shared arms are encoded with grp1/grp2 in vcalc().

> #	- Multiple concurrent outcomes: working sampling correlation rho = 0.50.

> #	- Crossover trials: paired standardized effects use r = 0.50, and

> #	off-diagonal sampling correlations are approximated with rho = 0.50.

> #	4) Direct timing subgroups are defined by comparison PAIR:

> #	Morning vs Evening, Afternoon vs Evening, Morning vs Afternoon.

> #	5) I-squared labels are corrected:

> #	sigma2[1] = between-study (Level 3)

> #	sigma2[2] = within-study (Level 2)

> #	6) Forest plots retain the metafor-native style used previously, but

> #	subgroup estimates are drawn directly and the overall estimate is

> #	added with addpoly(); no second meta-analysis is performed.

> #	7) CR2 cluster-robust inference (Study_ID clusters) is printed as a

> #	robustness analysis when estimable.

> #	8) Sensitivity analyses vary the assumed working correlations.

> #

> # Positive Hedges' g is oriented as a beneficial effect.

> # For direct timing contrasts, positive g favors the FIRST-named timing

> # category in Direct_Comparison_Pair.

> # =====================================================================

>

> # ---------------------------------------------------------------------

> # 0. Packages and analysis assumptions

> # ---------------------------------------------------------------------

> required_packages <- c("metafor", "dplyr", "clubSandwich", "Matrix")

> missing_packages <- required_packages[

+	!vapply(required_packages, requireNamespace, logical(1), quietly = TRUE)

+ ]

Registered S3 method overwritten by 'clubSandwich':

method	from

bread.mlm sandwich

>

> if (length(missing_packages) > 0) {

+	stop(

+	"Please install the following packages before running this script: ",

+	paste(missing_packages, collapse = ", ")

+	)

+ }

>

> library(metafor)

载入需要的程序包：Matrix

载入需要的程序包：metadat

载入需要的程序包：numDeriv

Loading the 'metafor' package (version 5.0-1). For an

introduction to the package please type: help(metafor)

警告信息:

程序包‘metafor’是用R版本4.6.1 来建造的

> library(dplyr)

载入程序包：‘dplyr’

The following objects are masked from ‘package:stats’:

filter, lag

The following objects are masked from ‘package:base’:

intersect, setdiff, setequal, union

警告信息:

程序包‘dplyr’是用R版本4.6.1 来建造的

> library(clubSandwich)

警告信息:

程序包‘clubSandwich’是用R版本4.6.1 来建造的

>

> PAIRED_R_PRIMARY <- 0.50

> SAMPLING_RHO_PRIMARY <- 0.50

>

> cat("=========================================================\n")

=========================================================

> cat("ANALYSIS ASSUMPTIONS\n")

ANALYSIS ASSUMPTIONS

> cat("=========================================================\n")

=========================================================

> cat(sprintf("Paired/crossover within-person correlation r = %.2f\n", PAIRED_R_PRIMARY))

Paired/crossover within-person correlation r = 0.50

> cat(sprintf("Working sampling-error correlation rho = %.2f\n", SAMPLING_RHO_PRIMARY))

Working sampling-error correlation rho = 0.50

> cat("Primary model: rma.mv with a full sampling variance-covariance matrix V.\n")

Primary model: rma.mv with a full sampling variance-covariance matrix V.

> cat("Random effects: ~ 1 | Study_ID / Effect_Size_ID\n")

Random effects: ~ 1 | Study_ID / Effect_Size_ID

> cat("CR2 robustness: cluster = Study_ID; Satterthwaite small-sample df.\n")

CR2 robustness: cluster = Study_ID; Satterthwaite small-sample df.

>

> # ---------------------------------------------------------------------

> # 1. Embedded data

> # ---------------------------------------------------------------------

>

> # ---- Model 1: Exercise vs. Control ----

> model1_data <- "Study_ID	Effect_Size_ID	Time_of_Day_Exp	Direction	N_Exp	Mean_Exp_Post	SD_Exp_Post	N_Ctrl	Mean_Ctrl_Post	SD_Ctrl_Post	Comparison_Type	Intervention_Length	Outcome_Domain	Shared_Control_ID

+ Iyer_2026	Iyer_2026_001	Morning (06:00–07:00)	Lower_Better	27	5.07	4.224	31	10.45	4.186	Exercise vs. Control	4	Sleep Quality	Iyer_Control

+ Iyer_2026	Iyer_2026_002	Morning (06:00–07:00)	Lower_Better	27	3.48	3.21	31	13.1	7.89	Exercise vs. Control	4	Mood	Iyer_Control

+ Iyer_2026	Iyer_2026_003	Morning (06:00–07:00)	Lower_Better	27	4.3	3.45	31	13.81	7.23	Exercise vs. Control	4	Mood	Iyer_Control

+ Iyer_2026	Iyer_2026_004	Morning (06:00–07:00)	Lower_Better	27	3.78	2.98	31	14.84	7.34	Exercise vs. Control	4	Mood	Iyer_Control

+ Iyer_2026	Iyer_2026_005	Evening (18:00–19:00)	Lower_Better	24	4.08	3.256	31	10.45	4.186	Exercise vs. Control	4	Sleep Quality	Iyer_Control

+ Iyer_2026	Iyer_2026_006	Evening (18:00–19:00)	Lower_Better	24	5.83	4.56	31	13.1	7.89	Exercise vs. Control	4	Mood	Iyer_Control

+ Iyer_2026	Iyer_2026_007	Evening (18:00–19:00)	Lower_Better	24	6.08	4.12	31	13.81	7.23	Exercise vs. Control	4	Mood	Iyer_Control

+ Iyer_2026	Iyer_2026_008	Evening (18:00–19:00)	Lower_Better	24	6.5	4.67	31	14.84	7.34	Exercise vs. Control	4	Mood	Iyer_Control

+ Shen_2025	Shen_2025_001	Morning (06:00–08:00)	Lower_Better	20	3.2	1.34	15	4.4	1.82	Exercise vs. Control	12 weeks	Sleep Quality	Shen_Control

+ Shen_2025	Shen_2025_002	Evening (18:00–20:00)	Lower_Better	23	3.17	1.77	15	4.4	1.82	Exercise vs. Control	12 weeks	Sleep Quality	Shen_Control

+ Vlahoyiannis_2020	Vlahoyiannis_2020_001	Evening (7–8 pm)	Higher_Better	10	397.2	87.5	10	352.2	93.9	Exercise vs. Control	Acute (single session)	Sleep Quality	Vlahoyiannis_2020_Shared_CON

+ Vlahoyiannis_2020	Vlahoyiannis_2020_002	Evening (7–8 pm)	Higher_Better	10	86.2	8.4	10	79.2	8.1	Exercise vs. Control	Acute (single session)	Sleep Quality	Vlahoyiannis_2020_Shared_CON

+ Vlahoyiannis_2020	Vlahoyiannis_2020_003	Evening (7–8 pm)	Higher_Better	10	357.7	98.6	10	352.2	93.9	Exercise vs. Control	Acute (single session)	Sleep Quality	Vlahoyiannis_2020_Shared_CON

+ Vlahoyiannis_2020	Vlahoyiannis_2020_004	Evening (7–8 pm)	Higher_Better	10	84.6	8.4	10	79.2	8.1	Exercise vs. Control	Acute (single session)	Sleep Quality	Vlahoyiannis_2020_Shared_CON

+ Vlahoyiannis_2020	Vlahoyiannis_2020_005	Evening (7–8 pm)	Higher_Better	10	378.3	62.1	10	352.2	93.9	Exercise vs. Control	Acute (single session)	Sleep Quality	Vlahoyiannis_2020_Shared_CON

+ Vlahoyiannis_2020	Vlahoyiannis_2020_006	Evening (7–8 pm)	Higher_Better	10	84.1	7.2	10	79.2	8.1	Exercise vs. Control	Acute (single session)	Sleep Quality	Vlahoyiannis_2020_Shared_CON

+ Maraki_2005	Maraki_2005_002	Morning (08:15–09:15)	Higher_Better	12	29.5	9.26	12	19.5	7.41	Exercise vs. Control	Acute (single session)	Mood	Maraki_2005_Shared_MC

+ Maraki_2005	Maraki_2005_003	Evening (19:15–20:15)	Higher_Better	12	29.5	7.78	12	22.5	7.56	Exercise vs. Control	Acute (single session)	Mood	Maraki_2005_Shared_EC

+ Maraki_2005	Maraki_2005_005	Morning (08:15–09:15)	Lower_Better	12	10.0	0.74	12	10.0	0.74	Exercise vs. Control	Acute (single session)	Mood	Maraki_2005_Shared_MC

+ Maraki_2005	Maraki_2005_006	Evening (19:15–20:15)	Lower_Better	12	10.0	0.74	12	10.0	0.74	Exercise vs. Control	Acute (single session)	Mood	Maraki_2005_Shared_EC"

>

> # ---- Model 2: direct Time vs. Time comparisons ----

> model2_data <- "Study_ID	Effect_Size_ID	Time_of_Day_Exp	Direction	N_Exp	Mean_Exp_Post	SD_Exp_Post	N_Ctrl	Mean_Ctrl_Post	SD_Ctrl_Post	Comparison_Type	Intervention_Length	Outcome_Domain

+ Iyer_2026	Iyer_2026_009	Morning (06:00–07:00)	Lower_Better	27	5.07	4.224	24	4.08	3.256	Time vs. Time	4	Sleep Quality

+ Iyer_2026	Iyer_2026_010	Morning (06:00–07:00)	Lower_Better	27	3.48	3.21	24	5.83	4.56	Time vs. Time	4	Mood

+ Iyer_2026	Iyer_2026_011	Morning (06:00–07:00)	Lower_Better	27	4.3	3.45	24	6.08	4.12	Time vs. Time	4	Mood

+ Iyer_2026	Iyer_2026_012	Morning (06:00–07:00)	Lower_Better	27	3.78	2.98	24	6.5	4.67	Time vs. Time	4	Mood

+ Saidi_2021	Saidi_2021_001	Morning (09:00)	Lower_Better	16	5.9	3.1	12	7.6	4.4	Time vs. Time	12	Sleep Quality

+ Saidi_2021	Saidi_2021_002	Morning (09:00)	Higher_Better	16	87.2	5.0	12	84.6	8.4	Time vs. Time	12	Sleep Quality

+ Saidi_2021	Saidi_2021_003	Morning (09:00)	Lower_Better	16	19.9	17.6	12	19.2	9.6	Time vs. Time	12	Sleep Quality

+ Saidi_2021	Saidi_2021_004	Morning (09:00)	Higher_Better	16	404.5	88.9	12	384.4	65.8	Time vs. Time	12	Sleep Quality

+ Shen_2025	Shen_2025_003	Morning (06:00–08:00)	Lower_Better	20	3.2	1.34	23	3.17	1.77	Time vs. Time	12 weeks	Sleep Quality

+ Arciero_2022	Arciero_2022_001	Morning (06:30–08:30)	Lower_Better	14	5.0	3.0	13	4.0	4.0	Time vs. Time	12 weeks	Mood

+ Arciero_2022	Arciero_2022_002	Morning (06:30–08:30)	Lower_Better	14	-2.0	12.0	13	-4.0	29.0	Time vs. Time	12 weeks	Mood

+ Arciero_2022	Arciero_2022_003	Morning (06:30–08:30)	Lower_Better	12	5.0	5.0	8	5.0	4.0	Time vs. Time	12 weeks	Mood

+ Arciero_2022	Arciero_2022_004	Morning (06:30–08:30)	Lower_Better	12	6.0	6.0	8	5.0	4.0	Time vs. Time	12 weeks	Mood

+ Arciero_2022	Arciero_2022_005	Morning (06:30–08:30)	Lower_Better	12	2.0	2.0	8	3.0	3.0	Time vs. Time	12 weeks	Mood

+ Arciero_2022	Arciero_2022_006	Morning (06:30–08:30)	Lower_Better	12	4.0	5.0	8	4.0	5.0	Time vs. Time	12 weeks	Mood

+ Arciero_2022	Arciero_2022_007	Morning (06:30–08:30)	Lower_Better	12	3.0	2.0	8	4.0	3.0	Time vs. Time	12 weeks	Mood

+ Arciero_2022	Arciero_2022_008	Morning (06:30–08:30)	Lower_Better	12	-3.0	20.0	8	4.0	12.0	Time vs. Time	12 weeks	Mood

+ Arciero_2022	Arciero_2022_009	Morning (06:30–08:30)	Higher_Better	12	21.0	8.0	8	18.0	4.0	Time vs. Time	12 weeks	Mood

+ Arciero_2022	Arciero_2022_010	Morning (06:30–08:30)	Higher_Better	14	20.0	4.0	13	21.0	7.0	Time vs. Time	12 weeks	Mood

+ Arciero_2022	Arciero_2022_011	Morning (06:30–08:30)	Lower_Better	14	2.0	2.0	13	4.0	8.0	Time vs. Time	12 weeks	Mood

+ Arciero_2022	Arciero_2022_012	Morning (06:30–08:30)	Lower_Better	14	3.0	3.0	13	3.0	4.0	Time vs. Time	12 weeks	Mood

+ Seol_2020	Seol_2020_001	Morning (waking–12:00)	Lower_Better	30	6.5	2.7	30	6.2	2.6	Time vs. Time	8 weeks	Sleep Quality

+ Seol_2020	Seol_2020_002	Morning (waking–12:00)	Higher_Better	29	89.2	4.4	28	89.5	5.6	Time vs. Time	8 weeks	Sleep Quality

+ Seol_2020	Seol_2020_003	Morning (waking–12:00)	Lower_Better	29	8.6	4.4	28	7.8	1.9	Time vs. Time	8 weeks	Sleep Quality

+ Seol_2020	Seol_2020_004	Morning (waking–12:00)	Higher_Better	29	416.8	53.0	28	400.9	57.3	Time vs. Time	8 weeks	Sleep Quality

+ Passos_2011	Passos_2011_001	Morning (10:00 ± 1h)	Lower_Better	10	10.5	6.0	9	6.7	5.4	Time vs. Time	6 months (3 sessions/week)	Sleep Quality

+ Passos_2011	Passos_2011_002	Morning (10:00 ± 1h)	Higher_Better	10	89.6	6.6	9	84.6	8.1	Time vs. Time	6 months (3 sessions/week)	Sleep Quality

+ Passos_2011	Passos_2011_003	Morning (10:00 ± 1h)	Lower_Better	10	4.8	3.8	9	1.7	4.2	Time vs. Time	6 months (3 sessions/week)	Mood

+ Passos_2011	Passos_2011_004	Morning (10:00 ± 1h)	Lower_Better	10	4.1	4.4	9	2.0	4.2	Time vs. Time	6 months (3 sessions/week)	Mood

+ Passos_2011	Passos_2011_005	Morning (10:00 ± 1h)	Lower_Better	10	4.8	3.8	9	1.4	3.9	Time vs. Time	6 months (3 sessions/week)	Mood

+ Passos_2011	Passos_2011_006	Morning (10:00 ± 1h)	Lower_Better	10	2.9	19.3	9	-7.2	20.4	Time vs. Time	6 months (3 sessions/week)	Mood

+ Passos_2011	Passos_2011_007	Morning (10:00 ± 1h)	Lower_Better	10	5.1	3.8	9	3.4	3.9	Time vs. Time	6 months (3 sessions/week)	Mood

+ Passos_2011	Passos_2011_008	Morning (10:00 ± 1h)	Lower_Better	10	1.7	3.8	9	2.9	4.2	Time vs. Time	6 months (3 sessions/week)	Mood

+ Passos_2011	Passos_2011_009	Morning (10:00 ± 1h)	Higher_Better	10	17.6	5.1	9	18.6	5.4	Time vs. Time	6 months (3 sessions/week)	Mood

+ Passos_2011	Passos_2011_010	Morning (10:00 ± 1h)	Higher_Better	10	6.2	2.2	9	5.5	1.8	Time vs. Time	6 months (3 sessions/week)	Sleep Quality

+ OConnor_1992	OConnor_1992_001	Morning (08:00)	Lower_Better	12	31.9	7.6	12	32.5	10.5	Time vs. Time	Acute (single session)	Mood

+ OConnor_1992	OConnor_1992_002	Morning (08:00)	Lower_Better	12	10.8	1.6	12	13.5	8.8	Time vs. Time	Acute (single session)	Mood

+ OConnor_1992	OConnor_1992_003	Afternoon (12:00)	Lower_Better	12	28.5	6.8	12	32.5	10.5	Time vs. Time	Acute (single session)	Mood

+ OConnor_1992	OConnor_1992_004	Afternoon (12:00)	Lower_Better	12	10.3	0.6	12	13.5	8.8	Time vs. Time	Acute (single session)	Mood

+ OConnor_1992	OConnor_1992_005	Afternoon (16:00)	Lower_Better	12	28.2	5.3	12	32.5	10.5	Time vs. Time	Acute (single session)	Mood

+ OConnor_1992	OConnor_1992_006	Afternoon (16:00)	Lower_Better	12	10.8	1.7	12	13.5	8.8	Time vs. Time	Acute (single session)	Mood

+ Teo_2021	Teo_2021_001	Morning (08:00–10:00)	Higher_Better	20	389.9	78.9	20	413.4	52.3	Time vs. Time	12 weeks (3 sessions/week)	Sleep Quality

+ Teo_2021	Teo_2021_002	Morning (08:00–10:00)	Higher_Better	20	83.6	6.9	20	86.6	5.3	Time vs. Time	12 weeks (3 sessions/week)	Sleep Quality

+ Teo_2021	Teo_2021_003	Morning (08:00–10:00)	Lower_Better	20	3.4	1.84	20	4.2	2.8	Time vs. Time	12 weeks (3 sessions/week)	Sleep Quality

+ Vitale_2016	Vitale_2016_001	Morning (08:00-09:00)	Higher_Better	12	88.5	5.5	12	78.3	7.4	Time vs. Time	Acute (single session)	Sleep Quality

+ Vitale_2016	Vitale_2016_002	Morning (08:00-09:00)	Higher_Better	11	83.0	6.0	11	86.0	5.0	Time vs. Time	Acute (single session)	Sleep Quality

+ Saidi_2023	Saidi_2023_001	Afternoon (13:00-15:00)	Higher_Better	12	89.6	6.9	12	86.4	8.3	Time vs. Time	Acute (single bout)	Sleep Quality

+ Saidi_2023	Saidi_2023_002	Afternoon (13:00-15:00)	Lower_Better	12	10.5	6.6	12	22.5	8.3	Time vs. Time	Acute (single bout)	Sleep Quality

+ Saidi_2023	Saidi_2023_003	Afternoon (13:00-15:00)	Lower_Better	12	34.2	5.5	12	43.8	12.8	Time vs. Time	Acute (single bout)	Mood

+ Saidi_2023	Saidi_2023_004	Afternoon (13:00-15:00)	Higher_Better	14	88.3	5.6	14	85.7	6.4	Time vs. Time	Acute (single bout)	Sleep Quality

+ Saidi_2023	Saidi_2023_005	Afternoon (13:00-15:00)	Lower_Better	14	34.0	7.85	14	40.9	6.4	Time vs. Time	Acute (single bout)	Mood

+ Saidi_2023	Saidi_2023_006	Afternoon (13:00-15:00)	Lower_Better	14	14.0	11.4	14	18.5	12.8	Time vs. Time	Acute (single bout)	Sleep Quality

+ Saidi_2023	Saidi_2023_007	Afternoon (13:00-15:00)	Higher_Better	16	83.4	9.2	16	83.6	8.8	Time vs. Time	Acute (single bout)	Sleep Quality

+ Saidi_2023	Saidi_2023_008	Afternoon (13:00-15:00)	Lower_Better	16	33.8	7.0	16	33.1	12.5	Time vs. Time	Acute (single bout)	Mood

+ Saidi_2023	Saidi_2023_009	Afternoon (13:00-15:00)	Lower_Better	16	18.2	11.7	16	17.4	12.6	Time vs. Time	Acute (single bout)	Sleep Quality

+ Fairbrother_2014	Fairbrother_2014_001	7:00 AM	Lower_Better	20	11.2	10.73	20	32.8	18.78	Time vs. Time	Acute (single session)	Sleep Quality

+ Fairbrother_2014	Fairbrother_2014_002	7:00 AM	Lower_Better	20	11.2	10.73	20	21.3	13.42	Time vs. Time	Acute (single session)	Sleep Quality

+ Fairbrother_2014	Fairbrother_2014_003	1:00 PM	Lower_Better	20	32.8	18.78	20	21.3	13.42	Time vs. Time	Acute (single session)	Sleep Quality

+ Fairbrother_2014	Fairbrother_2014_004	7:00 AM	Higher_Better	20	429.4	54.53	20	439.3	57.66	Time vs. Time	Acute (single session)	Sleep Quality

+ Fairbrother_2014	Fairbrother_2014_005	7:00 AM	Higher_Better	20	429.4	54.53	20	443.4	50.96	Time vs. Time	Acute (single session)	Sleep Quality

+ Fairbrother_2014	Fairbrother_2014_006	1:00 PM	Higher_Better	20	439.3	57.66	20	443.4	50.96	Time vs. Time	Acute (single session)	Sleep Quality"

>

> # ---------------------------------------------------------------------

> # 2. Read embedded data

> # ---------------------------------------------------------------------

> dat1 <- read.delim(

+	text = model1_data,

+	header = TRUE,

+	sep = "\t",

+	quote = "",

+	na.strings = "NA",

+	stringsAsFactors = FALSE,

+	check.names = FALSE

+ )

>

> dat2 <- read.delim(

+	text = model2_data,

+	header = TRUE,

+	sep = "\t",

+	quote = "",

+	na.strings = "NA",

+	stringsAsFactors = FALSE,

+	check.names = FALSE

+ )

>

> id_num <- function(x) {

+	as.integer(sub(".*_(\\d+)$", "\\1", x))

+ }

>

> # ---------------------------------------------------------------------

> # 3. Explicit design/dependence metadata

> # ---------------------------------------------------------------------

>

> add_model1_metadata <- function(d) {

+	num <- id_num(d$Effect_Size_ID)

+

+	d %>%

+	mutate(

+	Source_Model = "Model 1",

+	Direct_Comparison_Pair = NA_character_,

+

+	Design_Type = ifelse(

+	Study_ID %in% c("Vlahoyiannis_2020", "Maraki_2005"),

+	"Crossover",

+	"Parallel"

+	),

+

+	Sampling_Subgroup_ID = case_when(

+	Study_ID == "Iyer_2026" ~ "Iyer_All",

+	Study_ID == "Shen_2025" ~ "Shen_All",

+	Study_ID == "Vlahoyiannis_2020" ~ "Vlahoyiannis_All",

+	Study_ID == "Maraki_2005" ~ "Maraki_All",

+	TRUE ~ paste0(Study_ID, "_All")

+	),

+

+	# Arm identifiers are used for parallel trials by vcalc().

+	# For crossover rows they are retained for auditability, but

+	# the crossover sampling blocks are constructed separately.

+	Arm1_ID = case_when(

+	Study_ID == "Iyer_2026" & num %in% 1:4 ~ "Iyer_Morning",

+	Study_ID == "Iyer_2026" & num %in% 5:8 ~ "Iyer_Evening",

+	Effect_Size_ID == "Shen_2025_001" ~ "Shen_Morning",

+	Effect_Size_ID == "Shen_2025_002" ~ "Shen_Evening",

+

+	Study_ID == "Vlahoyiannis_2020" & num %in% c(1, 2) ~ "Vlahoyiannis_SIT",

+	Study_ID == "Vlahoyiannis_2020" & num %in% c(3, 4) ~ "Vlahoyiannis_MICT",

+	Study_ID == "Vlahoyiannis_2020" & num %in% c(5, 6) ~ "Vlahoyiannis_HIIT",

+

+	Study_ID == "Maraki_2005" & num %in% c(2, 5) ~ "Maraki_Morning_Exercise",

+	Study_ID == "Maraki_2005" & num %in% c(3, 6) ~ "Maraki_Evening_Exercise",

+	TRUE ~ paste0(Study_ID, "_Experimental")

+	),

+

+	Arm2_ID = case_when(

+	Study_ID == "Iyer_2026" ~ "Iyer_Control",

+	Study_ID == "Shen_2025" ~ "Shen_Control",

+	Study_ID == "Vlahoyiannis_2020" ~ "Vlahoyiannis_CON",

+	Study_ID == "Maraki_2005" & num %in% c(2, 5) ~ "Maraki_Morning_Control",

+	Study_ID == "Maraki_2005" & num %in% c(3, 6) ~ "Maraki_Evening_Control",

+	TRUE ~ paste0(Study_ID, "_Control")

+	),

+

+	# Same outcome labels are intentionally shared for the Iyer and Shen

+	# multi-arm contrasts so vcalc() can use the arm-overlap structure

+	# without imposing an additional rho for the same measured outcome.

+	Outcome_Obs_ID = case_when(

+	Study_ID == "Iyer_2026" & num %in% c(1, 5) ~ "Iyer_PSQI",

+	Study_ID == "Iyer_2026" & num %in% c(2, 6) ~ "Iyer_Depression",

+	Study_ID == "Iyer_2026" & num %in% c(3, 7) ~ "Iyer_Anxiety",

+	Study_ID == "Iyer_2026" & num %in% c(4, 8) ~ "Iyer_Stress",

+	Study_ID == "Shen_2025" ~ "Shen_PSQI",

+	TRUE ~ Effect_Size_ID

+	)

+	)

+ }

>

> add_model2_metadata <- function(d) {

+	num <- id_num(d$Effect_Size_ID)

+

+	d %>%

+	mutate(

+	Source_Model = "Model 2",

+

+	Direct_Comparison_Pair = case_when(

+	Study_ID %in% c(

+	"Iyer_2026", "Saidi_2021", "Shen_2025",

+	"Arciero_2022", "Seol_2020", "Passos_2011",

+	"Teo_2021", "Vitale_2016"

+	) ~ "Morning_vs_Evening",

+

+	Study_ID == "OConnor_1992" & num %in% 1:2 ~ "Morning_vs_Evening",

+	Study_ID == "OConnor_1992" & num %in% 3:6 ~ "Afternoon_vs_Evening",

+

+	Study_ID == "Saidi_2023" ~ "Afternoon_vs_Evening",

+

+	Study_ID == "Fairbrother_2014" & num %in% c(1, 4) ~ "Morning_vs_Afternoon",

+	Study_ID == "Fairbrother_2014" & num %in% c(2, 5) ~ "Morning_vs_Evening",

+	Study_ID == "Fairbrother_2014" & num %in% c(3, 6) ~ "Afternoon_vs_Evening",

+

+	TRUE ~ NA_character_

+	),

+

+	Design_Type = ifelse(

+	Study_ID %in% c(

+	"OConnor_1992", "Vitale_2016",

+	"Saidi_2023", "Fairbrother_2014"

+	),

+	"Crossover",

+	"Parallel"

+	),

+

+	# Independent participant subgroups within a study are separated here.

+	Sampling_Subgroup_ID = case_when(

+	Study_ID == "Iyer_2026" ~ "Iyer_All",

+	Study_ID == "Saidi_2021" ~ "Saidi2021_All",

+	Study_ID == "Shen_2025" ~ "Shen_All",

+

+	Study_ID == "Arciero_2022" & num %in% c(1, 2, 10, 11, 12) ~ "Arciero_Women",

+	Study_ID == "Arciero_2022" & num %in% 3:9 ~ "Arciero_Men",

+

+	Study_ID == "Seol_2020" ~ "Seol_All",

+	Study_ID == "Passos_2011" ~ "Passos_All",

+	Study_ID == "OConnor_1992" ~ "OConnor_All",

+	Study_ID == "Teo_2021" ~ "Teo_All",

+

+	Effect_Size_ID == "Vitale_2016_001" ~ "Vitale_MorningType",

+	Effect_Size_ID == "Vitale_2016_002" ~ "Vitale_EveningType",

+

+	Study_ID == "Saidi_2023" & num %in% 1:3 ~ "Saidi2023_Group12",

+	Study_ID == "Saidi_2023" & num %in% 4:6 ~ "Saidi2023_Group14",

+	Study_ID == "Saidi_2023" & num %in% 7:9 ~ "Saidi2023_Group16",

+

+	Study_ID == "Fairbrother_2014" ~ "Fairbrother_All",

+	TRUE ~ paste0(Study_ID, "_All")

+	),

+

+	# Arm identifiers for parallel studies. Iyer and Shen deliberately

+	# match Model 1 identifiers, which permits Model 3 to represent the

+	# covariance among Morning-vs-Control, Evening-vs-Control, and

+	# Morning-vs-Evening contrasts from the same trial.

+	Arm1_ID = case_when(

+	Study_ID == "Iyer_2026" ~ "Iyer_Morning",

+	Study_ID == "Shen_2025" ~ "Shen_Morning",

+

+	Design_Type == "Parallel" & Direct_Comparison_Pair == "Morning_vs_Evening" ~

+	paste0(Sampling_Subgroup_ID, "_Morning"),

+

+	Study_ID == "OConnor_1992" & num %in% 1:2 ~ "OConnor_Morning",

+	Study_ID == "OConnor_1992" & num %in% 3:4 ~ "OConnor_Afternoon12",

+	Study_ID == "OConnor_1992" & num %in% 5:6 ~ "OConnor_Afternoon16",

+	Study_ID == "Vitale_2016" ~ paste0(Sampling_Subgroup_ID, "_Morning"),

+	Study_ID == "Saidi_2023" ~ paste0(Sampling_Subgroup_ID, "_Afternoon"),

+	Study_ID == "Fairbrother_2014" & Direct_Comparison_Pair == "Morning_vs_Afternoon" ~ "Fairbrother_Morning",

+	Study_ID == "Fairbrother_2014" & Direct_Comparison_Pair == "Morning_vs_Evening" ~ "Fairbrother_Morning",

+	Study_ID == "Fairbrother_2014" & Direct_Comparison_Pair == "Afternoon_vs_Evening" ~ "Fairbrother_Afternoon",

+	TRUE ~ paste0(Sampling_Subgroup_ID, "_First")

+	),

+

+	Arm2_ID = case_when(

+	Study_ID == "Iyer_2026" ~ "Iyer_Evening",

+	Study_ID == "Shen_2025" ~ "Shen_Evening",

+

+	Design_Type == "Parallel" & Direct_Comparison_Pair == "Morning_vs_Evening" ~

+	paste0(Sampling_Subgroup_ID, "_Evening"),

+

+	Study_ID == "OConnor_1992" ~ "OConnor_Evening",

+	Study_ID == "Vitale_2016" ~ paste0(Sampling_Subgroup_ID, "_Evening"),

+	Study_ID == "Saidi_2023" ~ paste0(Sampling_Subgroup_ID, "_Evening"),

+	Study_ID == "Fairbrother_2014" & Direct_Comparison_Pair == "Morning_vs_Afternoon" ~ "Fairbrother_Afternoon",

+	Study_ID == "Fairbrother_2014" & Direct_Comparison_Pair == "Morning_vs_Evening" ~ "Fairbrother_Evening",

+	Study_ID == "Fairbrother_2014" & Direct_Comparison_Pair == "Afternoon_vs_Evening" ~ "Fairbrother_Evening",

+	TRUE ~ paste0(Sampling_Subgroup_ID, "_Second")

+	),

+

+	Outcome_Obs_ID = case_when(

+	Study_ID == "Iyer_2026" & num == 9 ~ "Iyer_PSQI",

+	Study_ID == "Iyer_2026" & num == 10 ~ "Iyer_Depression",

+	Study_ID == "Iyer_2026" & num == 11 ~ "Iyer_Anxiety",

+	Study_ID == "Iyer_2026" & num == 12 ~ "Iyer_Stress",

+	Study_ID == "Shen_2025" ~ "Shen_PSQI",

+	TRUE ~ Effect_Size_ID

+	)

+	)

+ }

>

> dat1 <- add_model1_metadata(dat1)

> dat2 <- add_model2_metadata(dat2)

>

> # Model 3 is the exact union of Models 1 and 2.

> dat3 <- bind_rows(dat1, dat2)

>

> # ---------------------------------------------------------------------

> # 4. Automatic data-integrity checks

> # ---------------------------------------------------------------------

> stopifnot(nrow(dat1) == 20L)

> stopifnot(nrow(dat2) == 61L)

> stopifnot(nrow(dat3) == 81L)

>

> stopifnot(sum(dat1$Outcome_Domain == "Sleep Quality") == 10L)

> stopifnot(sum(dat1$Outcome_Domain == "Mood") == 10L)

> stopifnot(sum(dat2$Outcome_Domain == "Sleep Quality") == 30L)

> stopifnot(sum(dat2$Outcome_Domain == "Mood") == 31L)

> stopifnot(sum(dat3$Outcome_Domain == "Sleep Quality") == 40L)

> stopifnot(sum(dat3$Outcome_Domain == "Mood") == 41L)

>

> stopifnot(all(dat1$Comparison_Type == "Exercise vs. Control"))

> stopifnot(all(dat2$Comparison_Type == "Time vs. Time"))

> stopifnot(!anyNA(dat2$Direct_Comparison_Pair))

>

> stopifnot(

+	identical(

+	dat1$Effect_Size_ID[dat1$Study_ID == "Iyer_2026"],

+	sprintf("Iyer_2026_%03d", 1:8)

+	)

+ )

>

> stopifnot(

+	identical(

+	dat2$Effect_Size_ID[dat2$Study_ID == "Iyer_2026"],

+	sprintf("Iyer_2026_%03d", 9:12)

+	)

+ )

>

> stopifnot(

+	dat1$Time_of_Day_Exp[dat1$Effect_Size_ID == "Iyer_2026_002"] ==

+	"Morning (06:00–07:00)"

+ )

>

> stopifnot(!"Maraki_2005_001" %in% dat2$Effect_Size_ID)

> stopifnot(!"Maraki_2005_004" %in% dat2$Effect_Size_ID)

>

> pair_counts <- dat2 %>%

+	count(Outcome_Domain, Direct_Comparison_Pair)

>

> # Exact expected pair structure after the Iyer correction.

> stopifnot(

+	pair_counts$n[

+	pair_counts$Outcome_Domain == "Sleep Quality" &

+	pair_counts$Direct_Comparison_Pair == "Morning_vs_Evening"

+	] == 20L

+ )

> stopifnot(

+	pair_counts$n[

+	pair_counts$Outcome_Domain == "Sleep Quality" &

+	pair_counts$Direct_Comparison_Pair == "Afternoon_vs_Evening"

+	] == 8L

+ )

> stopifnot(

+	pair_counts$n[

+	pair_counts$Outcome_Domain == "Sleep Quality" &

+	pair_counts$Direct_Comparison_Pair == "Morning_vs_Afternoon"

+	] == 2L

+ )

> stopifnot(

+	pair_counts$n[

+	pair_counts$Outcome_Domain == "Mood" &

+	pair_counts$Direct_Comparison_Pair == "Morning_vs_Evening"

+	] == 24L

+ )

> stopifnot(

+	pair_counts$n[

+	pair_counts$Outcome_Domain == "Mood" &

+	pair_counts$Direct_Comparison_Pair == "Afternoon_vs_Evening"

+	] == 7L

+ )

>

> cat("\n=========================================================\n")

=========================================================

> cat("DATA LOADED AND VERIFIED\n")

DATA LOADED AND VERIFIED

> cat("=========================================================\n")

=========================================================

> cat(sprintf("Model 1: %d rows (Sleep 10; Mood 10)\n", nrow(dat1)))

Model 1: 20 rows (Sleep 10; Mood 10)

> cat(sprintf("Model 2: %d rows (Sleep 30; Mood 31)\n", nrow(dat2)))

Model 2: 61 rows (Sleep 30; Mood 31)

> cat(sprintf("Model 3: %d rows (Sleep 40; Mood 41)\n", nrow(dat3)))

Model 3: 81 rows (Sleep 40; Mood 41)

> cat("\nModel 2 direct-comparison pair counts:\n")

Model 2 direct-comparison pair counts:

> print(pair_counts)

Outcome_Domain Direct_Comparison_Pair n

1	Mood	Afternoon_vs_Evening 7

2	Mood	Morning_vs_Evening 24

3 Sleep Quality	Afternoon_vs_Evening 8

4 Sleep Quality	Morning_vs_Afternoon 2

5 Sleep Quality	Morning_vs_Evening 20

>

> # Export exact enriched analysis datasets.

> write.csv(dat1, "Final_Model1_Data_With_Dependency_Metadata.csv", row.names = FALSE)

> write.csv(dat2, "Final_Model2_Data_With_Dependency_Metadata.csv", row.names = FALSE)

> write.csv(dat3, "Final_Model3_Data_With_Dependency_Metadata.csv", row.names = FALSE)

>

> # ---------------------------------------------------------------------

> # 5. Data cleaning

> # ---------------------------------------------------------------------

> clean_data <- function(data) {

+	data %>%

+	filter(

+	!is.na(N_Exp),

+	!is.na(Mean_Exp_Post),

+	!is.na(SD_Exp_Post),

+	!is.na(N_Ctrl),

+	!is.na(Mean_Ctrl_Post),

+	!is.na(SD_Ctrl_Post)

+	)

+ }

>

> dat1_clean <- clean_data(dat1)

> dat2_clean <- clean_data(dat2)

> dat3_clean <- clean_data(dat3)

>

> stopifnot(nrow(dat1_clean) == 20L)

> stopifnot(nrow(dat2_clean) == 61L)

> stopifnot(nrow(dat3_clean) == 81L)

>

> # ---------------------------------------------------------------------

> # 6. Effect-size calculation

> #

> # Parallel trials:

> #	Hedges' g from independent-group SMD.

> #

> # Crossover trials:

> #	Hedges-corrected standardized matched-pair effect using pooled raw SD

> #	(SMCRP), with assumed within-person correlation r = 0.50.

> #

> # All signs are harmonized so positive g = beneficial.

> # ---------------------------------------------------------------------

> calc_es <- function(data, domain = NULL, paired_r = PAIRED_R_PRIMARY) {

+	d <- data

+

+	if (!is.null(domain)) {

+	d <- d %>% filter(Outcome_Domain == domain)

+	}

+

+	if (nrow(d) == 0) return(NULL)

+

+	d <- d %>% mutate(.row_order = row_number())

+

+	d_parallel <- d %>% filter(Design_Type == "Parallel")

+	d_crossover <- d %>% filter(Design_Type == "Crossover")

+

+	out <- list()

+

+	if (nrow(d_parallel) > 0) {

+	es_parallel <- metafor::escalc(

+	measure = "SMD",

+	n1i = N_Exp,

+	m1i = Mean_Exp_Post,

+	sd1i = SD_Exp_Post,

+	n2i = N_Ctrl,

+	m2i = Mean_Ctrl_Post,

+	sd2i = SD_Ctrl_Post,

+	data = d_parallel

+	)

+

+	out[[length(out) + 1L]] <- as.data.frame(es_parallel)

+	}

+

+	if (nrow(d_crossover) > 0) {

+	if (any(d_crossover$N_Exp != d_crossover$N_Ctrl)) {

+	stop("Crossover rows require equal N_Exp and N_Ctrl in this script.")

+	}

+

+	es_crossover <- metafor::escalc(

+	measure = "SMCRP",

+	m1i = Mean_Exp_Post,

+	m2i = Mean_Ctrl_Post,

+	sd1i = SD_Exp_Post,

+	sd2i = SD_Ctrl_Post,

+	ri = paired_r,

+	ni = N_Exp,

+	data = d_crossover

+	)

+

+	out[[length(out) + 1L]] <- as.data.frame(es_crossover)

+	}

+

+	es <- bind_rows(out) %>%

+	arrange(.row_order) %>%

+	mutate(

+	yi = ifelse(

+	grepl("lower", tolower(Direction)),

+	-yi,

+	yi

+	),

+	Paired_r_Used = ifelse(Design_Type == "Crossover", paired_r, NA_real_)

+	)

+

+	es

+ }

>

> # ---------------------------------------------------------------------

> # 7. Sampling variance-covariance matrix V

> #

> # Parallel trials:

> #	vcalc() uses grp1/grp2 and group sizes to represent covariance induced

> #	by shared arms. Outcome_Obs_ID + rho represents unknown correlation

> #	among concurrently measured different outcomes.

> #

> # Crossover trials:

> #	effect-size variances already use paired r. Off-diagonal covariance

> #	among multiple estimates from the same participant subgroup is

> #	approximated with compound-symmetric working rho via vcalc().

> #

> # Independent subgroups within the same study are separated by

> # Sampling_Subgroup_ID and therefore receive zero off-diagonal covariance.

> # ---------------------------------------------------------------------

> build_V <- function(es, sampling_rho = SAMPLING_RHO_PRIMARY) {

+	if (is.null(es) || nrow(es) == 0) return(NULL)

+

+	V <- diag(es$vi)

+	rownames(V) <- es$Effect_Size_ID

+	colnames(V) <- es$Effect_Size_ID

+

+	idx_parallel <- which(es$Design_Type == "Parallel")

+	idx_crossover <- which(es$Design_Type == "Crossover")

+

+	if (length(idx_parallel) > 0) {

+	dp <- es[idx_parallel, , drop = FALSE]

+

+	Vp <- metafor::vcalc(

+	vi = vi,

+	cluster = Study_ID,

+	subgroup = Sampling_Subgroup_ID,

+	obs = Outcome_Obs_ID,

+	grp1 = Arm1_ID,

+	grp2 = Arm2_ID,

+	w1 = N_Exp,

+	w2 = N_Ctrl,

+	data = dp,

+	rho = sampling_rho,

+	checkpd = TRUE,

+	nearpd = TRUE

+	)

+

+	V[idx_parallel, idx_parallel] <- as.matrix(Vp)

+	}

+

+	if (length(idx_crossover) > 0) {

+	dc <- es[idx_crossover, , drop = FALSE]

+

+	Vc <- metafor::vcalc(

+	vi = vi,

+	cluster = Study_ID,

+	subgroup = Sampling_Subgroup_ID,

+	obs = Outcome_Obs_ID,

+	data = dc,

+	rho = sampling_rho,

+	checkpd = TRUE,

+	nearpd = TRUE

+	)

+

+	V[idx_crossover, idx_crossover] <- as.matrix(Vc)

+	}

+

+	# Numerical safeguard only. If the assembled matrix is not positive

+	# definite, use the nearest positive-definite matrix while preserving

+	# the diagonal. Any such adjustment is reported in the console.

+	eig_min <- min(eigen(V, symmetric = TRUE, only.values = TRUE)$values)

+

+	if (!is.finite(eig_min) || eig_min <= 1e-10) {

+	cat(sprintf(

+	"NOTE: V required nearPD adjustment (minimum eigenvalue = %.3e).\n",

+	eig_min

+	))

+	V <- as.matrix(Matrix::nearPD(V, keepDiag = TRUE)$mat)

+	rownames(V) <- es$Effect_Size_ID

+	colnames(V) <- es$Effect_Size_ID

+	}

+

+	V

+ }

>

> # ---------------------------------------------------------------------

> # 8. Main model fitting and CR2 robustness

> # ---------------------------------------------------------------------

> fit_3level <- function(es, V, label, mods = NULL) {

+	if (is.null(es) || nrow(es) < 2) {

+	cat("\nWARNING:", label, "- insufficient data; skipped.\n")

+	return(NULL)

+	}

+

+	if (is.null(mods)) {

+	res <- metafor::rma.mv(

+	yi = yi,

+	V = V,

+	random = ~ 1 | Study_ID / Effect_Size_ID,

+	data = es,

+	method = "REML"

+	)

+	} else {

+	res <- metafor::rma.mv(

+	yi = yi,

+	V = V,

+	mods = mods,

+	random = ~ 1 | Study_ID / Effect_Size_ID,

+	data = es,

+	method = "REML"

+	)

+	}

+

+	cat("\n=========================================================\n")

+	cat(label, "\n")

+	cat("=========================================================\n")

+	print(summary(res))

+

+	res

+ }

>

> run_cr2 <- function(res, es, label) {

+	if (is.null(res)) return(NULL)

+

+	n_clusters <- dplyr::n_distinct(es$Study_ID)

+

+	if (n_clusters < 3) {

+	cat(

+	"\n", label,

+	": CR2 not performed because fewer than 3 independent Study_ID clusters are available.\n",

+	sep = ""

+	)

+	return(NULL)

+	}

+

+	cr2 <- tryCatch(

+	metafor::robust(

+	res,

+	cluster = es$Study_ID,

+	clubSandwich = TRUE

+	),

+	error = function(e) {

+	message(label, " CR2 failed: ", e$message)

+	NULL

+	}

+	)

+

+	if (!is.null(cr2)) {

+	cat("\n", label, " - CR2 cluster-robust inference:\n", sep = "")

+	print(cr2)

+	}

+

+	cr2

+ }

>

> # ---------------------------------------------------------------------

> # 9. Model 1: Exercise vs Control

> # ---------------------------------------------------------------------

> cat("\n=========================================================\n")

=========================================================

> cat("MODEL 1: EXERCISE VS CONTROL\n")

MODEL 1: EXERCISE VS CONTROL

> cat("=========================================================\n")

=========================================================

>

> es1_sleep <- calc_es(dat1_clean, "Sleep Quality")

> es1_mood <- calc_es(dat1_clean, "Mood")

>

> V1_sleep <- build_V(es1_sleep)

> V1_mood <- build_V(es1_mood)

>

> res1_sleep <- fit_3level(

+	es1_sleep, V1_sleep,

+	"Model 1 - Sleep Quality (Exercise vs Control)"

+ )

=========================================================

Model 1 - Sleep Quality (Exercise vs Control)

=========================================================

Multivariate Meta-Analysis Model (k = 10; method: REML)

logLik Deviance	AIC	BIC	AICc

-2.6751	5.3502	11.3502	11.9419	16.1502

Variance Components:

estim	sqrt nlvls fixed	factor

sigma^2.1 0.2063 0.4542	3	no	Study_ID

sigma^2.2 0.0082 0.0904	10	no Study_ID/Effect_Size_ID

Test for Heterogeneity:

Q(df = 9) = 16.2330, p-val = 0.0622

Model Results:

estimate	se	zval	pval	ci.lb	ci.ub

0.8579 0.3073 2.7919 0.0052 0.2556 1.4602 **

---

Signif. codes: 0 ‘***’ 0.001 ‘**’ 0.01 ‘*’ 0.05 ‘.’ 0.1 ‘ ’ 1

>

> res1_mood <- fit_3level(

+	es1_mood, V1_mood,

+	"Model 1 - Mood (Exercise vs Control)"

+ )

=========================================================

Model 1 - Mood (Exercise vs Control)

=========================================================

Multivariate Meta-Analysis Model (k = 10; method: REML)

logLik Deviance	AIC	BIC	AICc

-6.2803	12.5606	18.5606	19.1523	23.3606

Variance Components:

estim	sqrt nlvls fixed	factor

sigma^2.1 0.4199 0.6480	2	no	Study_ID

sigma^2.2 0.1192 0.3452	10	no Study_ID/Effect_Size_ID

Test for Heterogeneity:

Q(df = 9) = 38.4405, p-val < .0001

Model Results:

estimate	se	zval	pval	ci.lb	ci.ub

0.9512 0.4980 1.9098 0.0562 -0.0250 1.9273 .

---

Signif. codes: 0 ‘***’ 0.001 ‘**’ 0.01 ‘*’ 0.05 ‘.’ 0.1 ‘ ’ 1

>

> res1_sleep_cr2 <- run_cr2(

+	res1_sleep, es1_sleep,

+	"Model 1 Sleep"

+ )

Model 1 Sleep - CR2 cluster-robust inference:

Multivariate Meta-Analysis Model (k = 10; method: REML)

Variance Components:

estim	sqrt nlvls fixed	factor

sigma^2.1 0.2063 0.4542	3	no	Study_ID

sigma^2.2 0.0082 0.0904	10	no Study_ID/Effect_Size_ID

Test for Heterogeneity:

Q(df = 9) = 16.2330, p-val = 0.0622

Number of estimates: 10

Number of clusters: 3

Estimates per cluster: 2-6 (mean: 3.33, median: 2)

Model Results:

estimate	se¹	tval¹	df¹	pval¹	ci.lb¹	ci.ub¹

0.8579 0.3086	2.7802	1.99	0.1091	-0.4739	2.1897

---

Signif. codes: 0 ‘***’ 0.001 ‘**’ 0.01 ‘*’ 0.05 ‘.’ 0.1 ‘ ’ 1

1) results based on cluster-robust inference (var-cov estimator: CR2,

approx t-test and confidence interval, df: Satterthwaite approx)

>

> res1_mood_cr2 <- run_cr2(

+	res1_mood, es1_mood,

+	"Model 1 Mood"

+ )

Model 1 Mood: CR2 not performed because fewer than 3 independent Study_ID clusters are available.

>

> # ---------------------------------------------------------------------

> # 10. Model 2: direct Time vs Time comparisons

> # ---------------------------------------------------------------------

> cat("\n=========================================================\n")

=========================================================

> cat("MODEL 2: DIRECT TIME VS TIME COMPARISONS\n")

MODEL 2: DIRECT TIME VS TIME COMPARISONS

> cat("=========================================================\n")

=========================================================

>

> es2_sleep <- calc_es(dat2_clean, "Sleep Quality")

> es2_mood <- calc_es(dat2_clean, "Mood")

>

> V2_sleep <- build_V(es2_sleep)

> V2_mood <- build_V(es2_mood)

>

> res2_sleep <- fit_3level(

+	es2_sleep, V2_sleep,

+	"Model 2 - Sleep Quality (Time vs Time)"

+ )

=========================================================

Model 2 - Sleep Quality (Time vs Time)

=========================================================

Multivariate Meta-Analysis Model (k = 30; method: REML)

logLik Deviance	AIC	BIC	AICc

-25.0017	50.0035	56.0035	60.1054	56.9635

Variance Components:

estim	sqrt nlvls fixed	factor

sigma^2.1 0.0000 0.0000	9	no	Study_ID

sigma^2.2 0.2382 0.4881	30	no Study_ID/Effect_Size_ID

Test for Heterogeneity:

Q(df = 29) = 148.0021, p-val < .0001

Model Results:

estimate	se	zval	pval	ci.lb	ci.ub

0.1505 0.1199 1.2554 0.2093 -0.0845 0.3855

---

Signif. codes: 0 ‘***’ 0.001 ‘**’ 0.01 ‘*’ 0.05 ‘.’ 0.1 ‘ ’ 1

>

> res2_mood <- fit_3level(

+	es2_mood, V2_mood,

+	"Model 2 - Mood (Time vs Time)"

+ )

=========================================================

Model 2 - Mood (Time vs Time)

=========================================================

Multivariate Meta-Analysis Model (k = 31; method: REML)

logLik Deviance	AIC	BIC	AICc

-9.9516	19.9031	25.9031	30.1067	26.8262

Variance Components:

estim	sqrt nlvls fixed	factor

sigma^2.1 0.0379 0.1946	5	no	Study_ID

sigma^2.2 0.0000 0.0000	31	no Study_ID/Effect_Size_ID

Test for Heterogeneity:

Q(df = 30) = 34.7592, p-val = 0.2515

Model Results:

estimate	se	zval	pval	ci.lb	ci.ub

0.2983 0.1352 2.2067 0.0273 0.0334 0.5633 *

---

Signif. codes: 0 ‘***’ 0.001 ‘**’ 0.01 ‘*’ 0.05 ‘.’ 0.1 ‘ ’ 1

>

> res2_sleep_cr2 <- run_cr2(

+	res2_sleep, es2_sleep,

+	"Model 2 Sleep"

+ )

Model 2 Sleep - CR2 cluster-robust inference:

Multivariate Meta-Analysis Model (k = 30; method: REML)

Variance Components:

estim	sqrt nlvls fixed	factor

sigma^2.1 0.0000 0.0000	9	no	Study_ID

sigma^2.2 0.2382 0.4881	30	no Study_ID/Effect_Size_ID

Test for Heterogeneity:

Q(df = 29) = 148.0021, p-val < .0001

Number of estimates: 30

Number of clusters: 9

Estimates per cluster: 1-6 (mean: 3.33, median: 3)

Model Results:

estimate	se¹	tval¹	df¹	pval¹	ci.lb¹	ci.ub¹

0.1505 0.0846	1.7791	5.8	0.1272	-0.0582	0.3593

---

Signif. codes: 0 ‘***’ 0.001 ‘**’ 0.01 ‘*’ 0.05 ‘.’ 0.1 ‘ ’ 1

1) results based on cluster-robust inference (var-cov estimator: CR2,

approx t-test and confidence interval, df: Satterthwaite approx)

>

> res2_mood_cr2 <- run_cr2(

+	res2_mood, es2_mood,

+	"Model 2 Mood"

+ )

Model 2 Mood - CR2 cluster-robust inference:

Multivariate Meta-Analysis Model (k = 31; method: REML)

Variance Components:

estim	sqrt nlvls fixed	factor

sigma^2.1 0.0379 0.1946	5	no	Study_ID

sigma^2.2 0.0000 0.0000	31	no Study_ID/Effect_Size_ID

Test for Heterogeneity:

Q(df = 30) = 34.7592, p-val = 0.2515

Number of estimates: 31

Number of clusters: 5

Estimates per cluster: 3-12 (mean: 6.20, median: 6)

Model Results:

estimate	se¹	tval¹	df¹	pval¹	ci.lb¹	ci.ub¹

0.2983 0.1346	2.2163	3.69	0.0967	-0.0882	0.6848	.

---

Signif. codes: 0 ‘***’ 0.001 ‘**’ 0.01 ‘*’ 0.05 ‘.’ 0.1 ‘ ’ 1

1) results based on cluster-robust inference (var-cov estimator: CR2,

approx t-test and confidence interval, df: Satterthwaite approx)

>

> # ---------------------------------------------------------------------

> # 11. Direct-comparison-pair subgroup analysis

> # ---------------------------------------------------------------------

> pair_levels <- c(

+	"Morning_vs_Evening",

+	"Afternoon_vs_Evening",

+	"Morning_vs_Afternoon"

+ )

>

> pretty_pair <- function(x) {

+	gsub("_vs_", " vs ", x, fixed = TRUE)

+ }

>

> run_pair_subgroup <- function(

+	es,

+	label,

+	sampling_rho = SAMPLING_RHO_PRIMARY) {

+

+	if (is.null(es) || nrow(es) < 2) return(NULL)

+

+	results <- list()

+

+	for (pair in pair_levels) {

+	sub <- es %>% filter(Direct_Comparison_Pair == pair)

+

+	if (nrow(sub) == 0) next

+

+	Vsub <- build_V(sub, sampling_rho = sampling_rho)

+	n_studies <- dplyr::n_distinct(sub$Study_ID)

+

+	# With >=2 independent studies, retain the three-level structure.

+	# With only one study, use a fixed-effects GLS synthesis with the

+	# non-diagonal V matrix and explicitly flag the estimate as single-study.

+	if (n_studies >= 2 && nrow(sub) >= 3) {

+	mod <- tryCatch(

+	metafor::rma.mv(

+	yi = yi,

+	V = Vsub,

+	random = ~ 1 | Study_ID / Effect_Size_ID,

+	data = sub,

+	method = "REML"

+	),

+	error = function(e) NULL

+	)

+	model_type <- "Three-level REML"

+	} else {

+	mod <- tryCatch(

+	metafor::rma.mv(

+	yi = yi,

+	V = Vsub,

+	data = sub

+	),

+	error = function(e) NULL

+	)

+	model_type <- "Fixed-effect GLS (single study)"

+	}

+

+	if (is.null(mod)) next

+

+	results[[length(results) + 1L]] <- data.frame(

+	Pair = pair,

+	Contrast = pretty_pair(pair),

+	Estimate = as.numeric(mod$b[1]),

+	CI_lower = as.numeric(mod$ci.lb),

+	CI_upper = as.numeric(mod$ci.ub),

+	P_value = as.numeric(mod$pval),

+	k = nrow(sub),

+	Studies = n_studies,

+	Model_Type = model_type,

+	stringsAsFactors = FALSE

+	)

+	}

+

+	if (length(results) == 0) return(NULL)

+

+	out <- bind_rows(results) %>%

+	mutate(

+	Pair = factor(Pair, levels = pair_levels)

+	) %>%

+	arrange(Pair)

+

+	cat("\n=========================================================\n")

+	cat(label, "\n")

+	cat("=========================================================\n")

+	print(out)

+

+	out

+ }

>

> sleep_pairs <- run_pair_subgroup(

+	es2_sleep,

+	"Model 2 Sleep - direct timing contrast subgroups"

+ )

=========================================================

Model 2 Sleep - direct timing contrast subgroups

=========================================================

Pair	Contrast	Estimate	CI_lower CI_upper	P_value k Studies

1	Morning_vs_Evening	Morning vs Evening 0.08493424 -0.1760636 0.3459320 0.5235947 20	8

2 Afternoon_vs_Evening Afternoon vs Evening 0.04528806 -0.6783431 0.7689193 0.9023738 8	2

3 Morning_vs_Afternoon Morning vs Afternoon 0.26382353 -0.1527894 0.6804365 0.2145455 2	1

Model_Type

1	Three-level REML

2	Three-level REML

3 Fixed-effect GLS (single study)

>

> mood_pairs <- run_pair_subgroup(

+	es2_mood,

+	"Model 2 Mood - direct timing contrast subgroups"

+ )

=========================================================

Model 2 Mood - direct timing contrast subgroups

=========================================================

Pair	Contrast Estimate	CI_lower CI_upper	P_value k Studies

1	Morning_vs_Evening	Morning vs Evening 0.1742300 -0.1716649 0.5201250 0.32352043 24	4

2 Afternoon_vs_Evening Afternoon vs Evening 0.4958561 0.1997086 0.7920036 0.00103192 7	2

Model_Type

1 Three-level REML

2 Three-level REML

>

> write.csv(

+	sleep_pairs,

+	"Direct_Comparison_Pair_Subgroups_Sleep.csv",

+	row.names = FALSE

+ )

>

> write.csv(

+	mood_pairs,

+	"Direct_Comparison_Pair_Subgroups_Mood.csv",

+	row.names = FALSE

+ )

>

> # ---------------------------------------------------------------------

> # 12. Forest plots: metafor-native style

> #	No re-pooling of subgroup estimates.

> # ---------------------------------------------------------------------

> plot_native_forest <- function(

+	subgroup_df,

+	overall_res,

+	title_text,

+	filename) {

+

+	if (is.null(subgroup_df) ||

+	nrow(subgroup_df) == 0 ||

+	is.null(overall_res)) {

+	return(NULL)

+	}

+

+	plot_df <- subgroup_df %>%

+	filter(

+	is.finite(Estimate),

+	is.finite(CI_lower),

+	is.finite(CI_upper)

+	)

+

+	if (nrow(plot_df) == 0) return(NULL)

+

+	# 由95% CI反推标准误和方差，仅用于绘图

+	sei_sub <- (plot_df$CI_upper - plot_df$CI_lower) / (2 * 1.96)

+	vi_sub <- sei_sub^2

+

+	# 标签缩短：不再显示 studies = X，避免与森林图区重叠

+	slab_labels <- sprintf(

+	"%s (k = %d)",

+	plot_df$Contrast,

+	plot_df$k

+	)

+

+	n_sub <- nrow(plot_df)

+

+	# 拉开各行距离

+	rows_sub <- seq(

+	from = n_sub + 3,

+	to = 4,

+	by = -1

+	)

+

+	png(

+	filename,

+	width = 3200,

+	height = 2400,

+	res = 300

+	)

+

+	par(

+	mar = c(5, 4, 4, 2) + 0.1,

+	family = "sans"

+	)

+

+	metafor::forest(

+	x = plot_df$Estimate,

+	vi = vi_sub,

+	slab = slab_labels,

+	rows = rows_sub,

+

+	# 增加左右留白，避免左侧文字与0线重合

+	ylim = c(0, n_sub + 7),

+	xlim = c(-5.0, 5.0),

+

+	# 真正的效应量横轴范围仍保持与旧图一致

+	alim = c(-2, 3),

+	at = c(-2, -1, 0, 1, 2, 3),

+

+	xlab = "Hedges' g (95% CI)",

+

+	# 稍微缩小字体

+	cex = 1.05,

+

+	header = c(

+	"Direct timing contrast",

+	"Hedges' g [95% CI]"

+	),

+

+	# 全部改成黑色，模仿原来的图

+	pch = 19,

+	col = "black",

+

+	# CI端点样式

+	efac = 0.7,

+

+	# 0效应参考线

+	refline = 0,

+

+	digits = 2

+	)

+

+	# 总体效应与亚组之间加横线

+	abline(h = 2.3, lty = 1, lwd = 1)

+

+	# 直接添加总体效应，不重新做第二次meta分析

+	# 保留你旧图中比较好看的蓝色总体diamond

+	metafor::addpoly(

+	x = as.numeric(overall_res$b[1]),

+	ci.lb = as.numeric(overall_res$ci.lb),

+	ci.ub = as.numeric(overall_res$ci.ub),

+	row = 1,

+

+	mlab = sprintf(

+	"Overall (k = %d)",

+	nrow(overall_res$data)

+	),

+

+	col = "#4FA3C7",

+	border = "black",

+	cex = 1.05

+	)

+

+	title(

+	main = title_text,

+	line = 2.2,

+	font.main = 2,

+	cex.main = 1.35

+	)

+

+	dev.off()

+

+	cat(sprintf("\nForest plot saved: %s\n", filename))

+ }

>

> plot_native_forest(

+	sleep_pairs,

+	res2_sleep,

+	"Exercise Timing and Sleep Quality (Direct Comparisons)",

+	"Forest_Plot_Sleep.png"

+ )

Forest plot saved: Forest_Plot_Sleep.png

>

> plot_native_forest(

+	mood_pairs,

+	res2_mood,

+	"Exercise Timing and Mood (Direct Comparisons)",

+	"Forest_Plot_Mood.png"

+ )

Forest plot saved: Forest_Plot_Mood.png

>

> # ---------------------------------------------------------------------

> # 13. Model 3: Comparison Type moderator - Sleep Quality

> # ---------------------------------------------------------------------

> cat("\n=========================================================\n")

=========================================================

> cat("MODEL 3: COMPARISON TYPE MODERATOR - SLEEP QUALITY\n")

MODEL 3: COMPARISON TYPE MODERATOR - SLEEP QUALITY

> cat("=========================================================\n")

=========================================================

>

> es3_sleep <- calc_es(dat3_clean, "Sleep Quality")

> V3_sleep <- build_V(es3_sleep)

>

> es3_sleep$Comparison_Type <- factor(

+	es3_sleep$Comparison_Type,

+	levels = c("Exercise vs. Control", "Time vs. Time")

+ )

>

> res3 <- metafor::rma.mv(

+	yi = yi,

+	V = V3_sleep,

+	mods = ~ Comparison_Type,

+	random = ~ 1 | Study_ID / Effect_Size_ID,

+	data = es3_sleep,

+	method = "REML"

+ )

>

> print(summary(res3))

Multivariate Meta-Analysis Model (k = 40; method: REML)

logLik Deviance	AIC	BIC	AICc

-30.2492	60.4984	68.4984	75.0487	69.7105

Variance Components:

estim	sqrt nlvls fixed	factor

sigma^2.1 0.0000 0.0000	10	no	Study_ID

sigma^2.2 0.1926 0.4388	40	no Study_ID/Effect_Size_ID

Test for Residual Heterogeneity:

QE(df = 38) = 500.4504, p-val < .0001

Test of Moderators (coefficient 2):

QM(df = 1) = 7.2834, p-val = 0.0070

Model Results:

estimate	se	zval	pval	ci.lb	ci.ub

intrcpt	0.8011 0.2139	3.7455 0.0002	0.3819	1.2202 ***

Comparison_TypeTime vs. Time	-0.6524 0.2417 -2.6988 0.0070 -1.1262 -0.1786	**

---

Signif. codes: 0 ‘***’ 0.001 ‘**’ 0.01 ‘*’ 0.05 ‘.’ 0.1 ‘ ’ 1

>

> res3_cr2 <- run_cr2(

+	res3, es3_sleep,

+	"Model 3 Sleep moderator"

+ )

Model 3 Sleep moderator - CR2 cluster-robust inference:

Multivariate Meta-Analysis Model (k = 40; method: REML)

Variance Components:

estim	sqrt nlvls fixed	factor

sigma^2.1 0.0000 0.0000	10	no	Study_ID

sigma^2.2 0.1926 0.4388	40	no Study_ID/Effect_Size_ID

Test for Residual Heterogeneity:

QE(df = 38) = 500.4504, p-val < .0001

Number of estimates: 40

Number of clusters: 10

Estimates per cluster: 2-6 (mean: 4.00, median: 3.5)

Test of Moderators (coefficient 2):¹

F(df1 = 1, df2 = 2.84) = 3.8779, p-val = 0.1486

Model Results:

estimate	se¹	tval¹	df¹	pval¹	ci.lb¹	ci.ub¹

intrcpt	0.8011 0.3100	2.5839	1.83	0.1341	-0.6592	2.2613

Comparison_TypeTime vs. Time	-0.6524 0.3313	-1.9692	2.84	0.1486	-1.7402	0.4354

---

Signif. codes: 0 ‘***’ 0.001 ‘**’ 0.01 ‘*’ 0.05 ‘.’ 0.1 ‘ ’ 1

1) results based on cluster-robust inference (var-cov estimator: CR2,

approx t/F-tests and confidence intervals, df: Satterthwaite approx)

>

> pred_exercise_control <- predict(res3, newmods = 0)

> pred_time_time <- predict(res3, newmods = 1)

>

> cat("\nModel 3 predicted estimates by comparison type:\n")

Model 3 predicted estimates by comparison type:

> cat(sprintf(

+	"Exercise vs Control: g = %.4f [%.4f, %.4f]\n",

+	pred_exercise_control$pred,

+	pred_exercise_control$ci.lb,

+	pred_exercise_control$ci.ub

+ ))

Exercise vs Control: g = 0.8011 [0.3819, 1.2202]

> cat(sprintf(

+	"Time vs Time: g = %.4f [%.4f, %.4f]\n",

+	pred_time_time$pred,

+	pred_time_time$ci.lb,

+	pred_time_time$ci.ub

+ ))

Time vs Time: g = 0.1487 [-0.0722, 0.3695]

>

> # ---------------------------------------------------------------------

> # 14. Corrected I-squared decomposition

> #

> # For random = ~1 | Study_ID / Effect_Size_ID:

> #	sigma2[1] = Study_ID variance	= Level 3 / between-study

> #	sigma2[2] = nested Effect_Size variance = Level 2 / within-study

> # ---------------------------------------------------------------------

> calc_i2_3level <- function(model, V) {

+	if (is.null(model) || length(model$sigma2) < 2) return(NULL)

+

+	sigma2_between <- model$sigma2[1]

+	sigma2_within <- model$sigma2[2]

+	typical_sampling_var <- median(diag(V), na.rm = TRUE)

+

+	total_var <- sigma2_between + sigma2_within + typical_sampling_var

+

+	data.frame(

+	Level = c(

+	"Level 2 (Within study)",

+	"Level 3 (Between study)",

+	"Total"

+	),

+	I2_Percent = c(

+	sigma2_within / total_var * 100,

+	sigma2_between / total_var * 100,

+	(sigma2_within + sigma2_between) / total_var * 100

+	)

+	)

+ }

>

> i2_sleep <- calc_i2_3level(res2_sleep, V2_sleep)

> i2_mood <- calc_i2_3level(res2_mood, V2_mood)

>

> cat("\nModel 2 Sleep - corrected I-squared decomposition:\n")

Model 2 Sleep - corrected I-squared decomposition:

> print(i2_sleep)

Level	I2_Percent

1 Level 2 (Within study) 7.402934e+01

2 Level 3 (Between study) 6.147471e-08

3	Total 7.402935e+01

>

> cat("\nModel 2 Mood - corrected I-squared decomposition:\n")

Model 2 Mood - corrected I-squared decomposition:

> print(i2_mood)

Level	I2_Percent

1 Level 2 (Within study) 1.169990e-07

2 Level 3 (Between study) 2.018498e+01

3	Total 2.018498e+01

>

> write.csv(i2_sleep, "I2_Decomposition_Sleep.csv", row.names = FALSE)

> write.csv(i2_mood, "I2_Decomposition_Mood.csv", row.names = FALSE)

>

> # ---------------------------------------------------------------------

> # 15. Leave-one-study-out sensitivity analysis: Model 2 Sleep

> # ---------------------------------------------------------------------

> leave_one_study_out <- function(

+	es,

+	label,

+	sampling_rho = SAMPLING_RHO_PRIMARY) {

+

+	studies <- sort(unique(es$Study_ID))

+

+	out <- lapply(studies, function(study_to_remove) {

+	d <- es %>% filter(Study_ID != study_to_remove)

+	Vd <- build_V(d, sampling_rho = sampling_rho)

+

+	mod <- tryCatch(

+	metafor::rma.mv(

+	yi = yi,

+	V = Vd,

+	random = ~ 1 | Study_ID / Effect_Size_ID,

+	data = d,

+	method = "REML"

+	),

+	error = function(e) NULL

+	)

+

+	if (is.null(mod)) {

+	return(data.frame(

+	Excluded_Study = study_to_remove,

+	k = nrow(d),

+	Studies = dplyr::n_distinct(d$Study_ID),

+	Estimate = NA_real_,

+	CI_lower = NA_real_,

+	CI_upper = NA_real_,

+	P_value = NA_real_

+	))

+	}

+

+	data.frame(

+	Excluded_Study = study_to_remove,

+	k = nrow(d),

+	Studies = dplyr::n_distinct(d$Study_ID),

+	Estimate = as.numeric(mod$b[1]),

+	CI_lower = as.numeric(mod$ci.lb),

+	CI_upper = as.numeric(mod$ci.ub),

+	P_value = as.numeric(mod$pval)

+	)

+	}) %>% bind_rows()

+

+	cat("\n=========================================================\n")

+	cat(label, "\n")

+	cat("=========================================================\n")

+	print(out)

+

+	out

+ }

>

> loo_sleep <- leave_one_study_out(

+	es2_sleep,

+	"Model 2 Sleep - Leave-one-study-out analysis"

+ )

=========================================================

Model 2 Sleep - Leave-one-study-out analysis

=========================================================

Excluded_Study k Studies	Estimate	CI_lower CI_upper	P_value

1 Fairbrother_2014 24	8 0.14984405 -0.07492206 0.3746102 0.1913342

2	Iyer_2026 29	8 0.17009016 -0.07198692 0.4121672 0.1684734

3	Passos_2011 27	8 0.15276115 -0.09003750 0.3955598 0.2175210

4	Saidi_2021 26	8 0.14172828 -0.11704668 0.4005032 0.2830687

5	Saidi_2023 24	8 0.07736903 -0.19604919 0.3507872 0.5791614

6	Seol_2020 26	8 0.18257001 -0.08381410 0.4489541 0.1791787

7	Shen_2025 29	8 0.15877247 -0.08434350 0.4018884 0.2005453

8	Teo_2021 27	8 0.18535548 -0.06587168 0.4365826 0.1481598

9	Vitale_2016 28	8 0.12321777 -0.11000685 0.3564424 0.3004389

>

> write.csv(

+	loo_sleep,

+	"Leave_One_Out_Sleep.csv",

+	row.names = FALSE

+ )

>

> # ---------------------------------------------------------------------

> # 16. High-risk-of-bias sensitivity: Model 2 Sleep

> # ---------------------------------------------------------------------

> high_risk_studies <- c(

+	"Saidi_2021",

+	"Arciero_2022",

+	"Passos_2011",

+	"Vitale_2016"

+ )

> es2_sleep_lowrob <- es2_sleep %>%

+	filter(!Study_ID %in% high_risk_studies)

>

> V2_sleep_lowrob <- build_V(es2_sleep_lowrob)

>

> cat("\n=========================================================\n")

=========================================================

> cat("MODEL 2 SLEEP - EXCLUDING HIGH-RISK-OF-BIAS STUDIES\n")

MODEL 2 SLEEP - EXCLUDING HIGH-RISK-OF-BIAS STUDIES

> cat("=========================================================\n")

=========================================================

> cat(sprintf(

+	"Remaining: %d effect sizes from %d studies\n",

+	nrow(es2_sleep_lowrob),

+	dplyr::n_distinct(es2_sleep_lowrob$Study_ID)

+ ))

Remaining: 21 effect sizes from 6 studies

>

> res_sleep_lowrob <- metafor::rma.mv(

+	yi = yi,

+	V = V2_sleep_lowrob,

+	random = ~ 1 | Study_ID / Effect_Size_ID,

+	data = es2_sleep_lowrob,

+	method = "REML"

+ )

>

> print(summary(res_sleep_lowrob))

Multivariate Meta-Analysis Model (k = 21; method: REML)

logLik Deviance	AIC	BIC	AICc

-16.4092	32.8184	38.8184	41.8056	40.3184

Variance Components:

estim	sqrt nlvls fixed	factor

sigma^2.1 0.0000 0.0000	6	no	Study_ID

sigma^2.2 0.2307 0.4803	21	no Study_ID/Effect_Size_ID

Test for Heterogeneity:

Q(df = 20) = 119.4749, p-val < .0001

Model Results:

estimate	se	zval	pval	ci.lb	ci.ub

0.1104 0.1361 0.8112 0.4173 -0.1563 0.3771

---

Signif. codes: 0 ‘***’ 0.001 ‘**’ 0.01 ‘*’ 0.05 ‘.’ 0.1 ‘ ’ 1

>

> # ---------------------------------------------------------------------

> # 17. Working-correlation sensitivity analysis

> #

> # Scenario A: vary paired/crossover r, keep sampling rho = .50.

> # Scenario B: vary sampling rho, keep paired r = .50.

> #

> # These scenarios are run for Models 1 and 2 so the reviewer can directly

> # inspect sensitivity to the working correlation assumptions.

> # ---------------------------------------------------------------------

> fit_scenario <- function(

+	source_data,

+	model_name,

+	domain,

+	paired_r,

+	sampling_rho,

+	scenario_label) {

+

+	es <- calc_es(

+	source_data,

+	domain = domain,

+	paired_r = paired_r

+	)

+

+	V <- build_V(

+	es,

+	sampling_rho = sampling_rho

+	)

+

+	mod <- metafor::rma.mv(

+	yi = yi,

+	V = V,

+	random = ~ 1 | Study_ID / Effect_Size_ID,

+	data = es,

+	method = "REML"

+	)

+

+	data.frame(

+	Scenario = scenario_label,

+	Model = model_name,

+	Outcome = domain,

+	Paired_r = paired_r,

+	Sampling_rho = sampling_rho,

+	k = nrow(es),

+	Studies = dplyr::n_distinct(es$Study_ID),

+	Estimate = as.numeric(mod$b[1]),

+	CI_lower = as.numeric(mod$ci.lb),

+	CI_upper = as.numeric(mod$ci.ub),

+	P_value = as.numeric(mod$pval),

+	stringsAsFactors = FALSE

+	)

+ }

>

> sensitivity_scenarios <- bind_rows(

+	data.frame(

+	Scenario = c("Primary", "Paired r = .30", "Paired r = .70"),

+	Paired_r = c(.50, .30, .70),

+	Sampling_rho = c(.50, .50, .50)

+	),

+	data.frame(

+	Scenario = c("Sampling rho = .30", "Sampling rho = .70"),

+	Paired_r = c(.50, .50),

+	Sampling_rho = c(.30, .70)

+	)

+ )

>

> analysis_sources <- list(

+	"Model 1" = dat1_clean,

+	"Model 2" = dat2_clean

+ )

>

> corr_sensitivity <- bind_rows(

+	lapply(names(analysis_sources), function(model_name) {

+	source_data <- analysis_sources[[model_name]]

+

+	bind_rows(lapply(c("Sleep Quality", "Mood"), function(domain) {

+	bind_rows(lapply(seq_len(nrow(sensitivity_scenarios)), function(i) {

+	fit_scenario(

+	source_data = source_data,

+	model_name = model_name,

+	domain = domain,

+	paired_r = sensitivity_scenarios$Paired_r[i],

+	sampling_rho = sensitivity_scenarios$Sampling_rho[i],

+	scenario_label = sensitivity_scenarios$Scenario[i]

+	)

+	}))

+	}))

+	})

+ )

>

> cat("\n=========================================================\n")

=========================================================

> cat("WORKING-CORRELATION SENSITIVITY - MODELS 1 AND 2\n")

WORKING-CORRELATION SENSITIVITY - MODELS 1 AND 2

> cat("=========================================================\n")

=========================================================

> print(corr_sensitivity)

Scenario	Model	Outcome Paired_r Sampling_rho k Studies Estimate	CI_lower

1	Primary Model 1 Sleep Quality	0.5	0.5 10	3 0.8579250 0.255643813

2	Paired r = .30 Model 1 Sleep Quality	0.3	0.5 10	3 0.8757675 0.283252380

3	Paired r = .70 Model 1 Sleep Quality	0.7	0.5 10	3 0.8391629 0.226203752

4 Sampling rho = .30 Model 1 Sleep Quality	0.5	0.3 10	3 0.8535832 0.261631849

5 Sampling rho = .70 Model 1 Sleep Quality	0.5	0.7 10	3 0.8627071 0.251789768

6	Primary Model 1	Mood	0.5	0.5 10	2 0.9511518 -0.024980791

7	Paired r = .30 Model 1	Mood	0.3	0.5 10	2 0.9644489 -0.001260637

8	Paired r = .70 Model 1	Mood	0.7	0.5 10	2 0.9354011 -0.055214948

9 Sampling rho = .30 Model 1	Mood	0.5	0.3 10	2 0.9535899 -0.013597186

10 Sampling rho = .70 Model 1	Mood	0.5	0.7 10	2 0.9493657 -0.035053217

11	Primary Model 2 Sleep Quality	0.5	0.5 30	9 0.1505124 -0.084462603

12	Paired r = .30 Model 2 Sleep Quality	0.3	0.5 30	9 0.1458808 -0.092612979

13	Paired r = .70 Model 2 Sleep Quality	0.7	0.5 30	9 0.1532088 -0.076687987

14 Sampling rho = .30 Model 2 Sleep Quality	0.5	0.3 30	9 0.1501733 -0.071164587

15 Sampling rho = .70 Model 2 Sleep Quality	0.5	0.7 30	9 0.1507674 -0.097130506

16	Primary Model 2	Mood	0.5	0.5 31	5 0.2983374 0.033355896

17	Paired r = .30 Model 2	Mood	0.3	0.5 31	5 0.2920477 0.005567242

18	Paired r = .70 Model 2	Mood	0.7	0.5 31	5 0.3054591 0.062500719

19 Sampling rho = .30 Model 2	Mood	0.5	0.3 31	5 0.2677582 -0.026308099

20 Sampling rho = .70 Model 2	Mood	0.5	0.7 31	5 0.3281501 0.076722756

CI_upper	P_value

1 1.4602062 0.005240136

2 1.4682825 0.003768380

3 1.4521221 0.007290818

4 1.4455345 0.004709901

5 1.4736244 0.005644235

6 1.9272843 0.056158277

7 1.9301585 0.050299818

8 1.9260172 0.064209913

9 1.9207770 0.053308938

10 1.9337846 0.058734755

11 0.3854874 0.209316191

12 0.3843746 0.230581608

13 0.3831056 0.191495572

14 0.3715112 0.183585468

15 0.3986654 0.233254301

16 0.5633189 0.027336114

17 0.5785281 0.045710976

18 0.5484174 0.013733396

19 0.5618245 0.074323068

20 0.5795774 0.010526255

>

> write.csv(

+	corr_sensitivity,

+	"Working_Correlation_Sensitivity_Models1_2.csv",

+	row.names = FALSE

+ )

>

> # ---------------------------------------------------------------------

> # 17b. Median/IQR-converted data audit

> #

> # Maraki_2005 contributes median/IQR-converted data to Model 1 Mood.

> # The two Maraki direct Time-vs-Time rows are not in Model 2.

> # Excluding Maraki from Model 1 Mood leaves only Iyer_2026 (one study),

> # so a pooled Model 1 Mood sensitivity estimate is not identifiable.

> # ---------------------------------------------------------------------

> maraki_model1_rows <- dat1_clean %>%

+	filter(Study_ID == "Maraki_2005")

>

> cat("\n=========================================================\n")

=========================================================

> cat("MEDIAN/IQR-CONVERTED DATA AUDIT\n")

MEDIAN/IQR-CONVERTED DATA AUDIT

> cat("=========================================================\n")

=========================================================

> cat(sprintf(

+	"Maraki_2005 contributes %d Model 1 Mood effect sizes converted from median/IQR.\n",

+	nrow(maraki_model1_rows)

+ ))

Maraki_2005 contributes 4 Model 1 Mood effect sizes converted from median/IQR.

> cat("Maraki_2005 direct Time-vs-Time rows are excluded from Model 2.\n")

Maraki_2005 direct Time-vs-Time rows are excluded from Model 2.

>

> model1_mood_no_maraki <- dat1_clean %>%

+	filter(

+	Outcome_Domain == "Mood",

+	Study_ID != "Maraki_2005"

+	)

>

> cat(sprintf(

+	"After excluding Maraki_2005, Model 1 Mood contains %d effect sizes from %d study/studies; pooled sensitivity is therefore not estimated.\n",

+	nrow(model1_mood_no_maraki),

+	dplyr::n_distinct(model1_mood_no_maraki$Study_ID)

+ ))

After excluding Maraki_2005, Model 1 Mood contains 6 effect sizes from 1 study/studies; pooled sensitivity is therefore not estimated.

>

> # ---------------------------------------------------------------------

> # 18. Exploratory Egger-type regression and funnel plots

> #	The same non-diagonal V matrix is used in the regression.

> #	Interpret cautiously because the number of independent studies is small.

> # ---------------------------------------------------------------------

> run_egger <- function(es, V, overall_model, label, filename, main_title) {

+	cat("\n=========================================================\n")

+	cat(label, "\n")

+	cat("=========================================================\n")

+

+	egger <- tryCatch(

+	metafor::rma.mv(

+	yi = yi,

+	V = V,

+	mods = ~ sqrt(vi),

+	random = ~ 1 | Study_ID / Effect_Size_ID,

+	data = es,

+	method = "REML"

+	),

+	error = function(e) {

+	message("Egger-type regression failed: ", e$message)

+	NULL

+	}

+	)

+

+	if (!is.null(egger)) {

+	print(summary(egger))

+	}

+

+	# The funnel display is descriptive. The fitted Model 2 object is used

+	# only for plotting; formal interpretation comes from the multilevel

+	# regression above.

+	png(

+	filename,

+	width = 2400,

+	height = 2000,

+	res = 300

+	)

+	metafor::funnel(

+	x = es$yi,

+	vi = es$vi,

+	refline = as.numeric(overall_model$b[1]),

+	main = main_title,

+	xlab = "Hedges' g",

+	las = 1

+	)

+	dev.off()

+

+	cat(sprintf("Funnel plot saved: %s\n", filename))

+

+	egger

+ }

>

> egger_sleep <- run_egger(

+	es2_sleep,

+	V2_sleep,

+	res2_sleep,

+	"SLEEP - EXPLORATORY EGGER-TYPE REGRESSION",

+	"Funnel_Plot_Sleep.png",

+	"Funnel Plot: Sleep Quality (Time vs Time)"

+ )

=========================================================

SLEEP - EXPLORATORY EGGER-TYPE REGRESSION

=========================================================

Multivariate Meta-Analysis Model (k = 30; method: REML)

logLik Deviance	AIC	BIC	AICc

-22.9683	45.9366	53.9366	59.2655	55.6758

Variance Components:

estim	sqrt nlvls fixed	factor

sigma^2.1 0.0000 0.0000	9	no	Study_ID

sigma^2.2 0.2041 0.4518	30	no Study_ID/Effect_Size_ID

Test for Residual Heterogeneity:

QE(df = 28) = 121.7116, p-val < .0001

Test of Moderators (coefficient 2):

QM(df = 1) = 3.1456, p-val = 0.0761

Model Results:

estimate	se	zval	pval	ci.lb	ci.ub

intrcpt	-0.7915 0.5420 -1.4602 0.1442 -1.8539 0.2709

sqrt(vi)	3.1156 1.7566	1.7736 0.0761 -0.3274 6.5585 .

---

Signif. codes: 0 ‘***’ 0.001 ‘**’ 0.01 ‘*’ 0.05 ‘.’ 0.1 ‘ ’ 1

Funnel plot saved: Funnel_Plot_Sleep.png

> egger_mood <- run_egger(

+	es2_mood,

+	V2_mood,

+	res2_mood,

+	"MOOD - EXPLORATORY EGGER-TYPE REGRESSION",

+	"Funnel_Plot_Mood.png",

+	"Funnel Plot: Mood (Time vs Time)"

+ )

=========================================================

MOOD - EXPLORATORY EGGER-TYPE REGRESSION

=========================================================

Multivariate Meta-Analysis Model (k = 31; method: REML)

logLik Deviance	AIC	BIC	AICc

-8.5436	17.0871	25.0871	30.5563	26.7538

Variance Components:

estim	sqrt nlvls fixed	factor

sigma^2.1 0.0000 0.0000	5	no	Study_ID

sigma^2.2 0.0056 0.0750	31	no Study_ID/Effect_Size_ID

Test for Residual Heterogeneity:

QE(df = 29) = 32.4167, p-val = 0.3019

Test of Moderators (coefficient 2):

QM(df = 1) = 2.5085, p-val = 0.1132

Model Results:

estimate	se	zval	pval	ci.lb	ci.ub

intrcpt	1.0794 0.4828	2.2357 0.0254	0.1331 2.0256 *

sqrt(vi)	-2.2771 1.4377 -1.5838 0.1132 -5.0951 0.5408

---

Signif. codes: 0 ‘***’ 0.001 ‘**’ 0.01 ‘*’ 0.05 ‘.’ 0.1 ‘ ’ 1

Funnel plot saved: Funnel_Plot_Mood.png

>

> # ---------------------------------------------------------------------

> # 19. Compact key-results table

> # ---------------------------------------------------------------------

> extract_main_result <- function(model, model_name, outcome_name) {

+	if (is.null(model)) return(NULL)

+

+	data.frame(

+	Model = model_name,

+	Outcome = outcome_name,

+	k = model$k,

+	Studies = dplyr::n_distinct(model$data$Study_ID),

+	Hedges_g = as.numeric(model$b[1]),

+	CI_lower = as.numeric(model$ci.lb),

+	CI_upper = as.numeric(model$ci.ub),

+	p_value = as.numeric(model$pval),

+	Q = if (!is.null(model$QE)) as.numeric(model$QE) else NA_real_,

+	Q_df = if (!is.null(model$k)) model$k - length(model$b) else NA_real_,

+	Q_p = if (!is.null(model$QEp)) as.numeric(model$QEp) else NA_real_,

+	stringsAsFactors = FALSE

+	)

+ }

>

> main_results <- bind_rows(

+	extract_main_result(res1_sleep, "Model 1", "Sleep Quality"),

+	extract_main_result(res1_mood, "Model 1", "Mood"),

+	extract_main_result(res2_sleep, "Model 2", "Sleep Quality"),

+	extract_main_result(res2_mood, "Model 2", "Mood")

+ )

>

> cat("\n=========================================================\n")

=========================================================

> cat("KEY RESULTS\n")

KEY RESULTS

> cat("=========================================================\n")

=========================================================

> print(main_results)

Model	Outcome k Studies Hedges_g	CI_lower CI_upper	p_value	Q Q_df	Q_p

1 Model 1 Sleep Quality 10	3 0.8579250 0.25564381 1.4602062 0.005240136 16.23296	9 6.217323e-02

2 Model 1	Mood 10	2 0.9511518 -0.02498079 1.9272843 0.056158277 38.44054	9 1.452811e-05

3 Model 2 Sleep Quality 30	9 0.1505124 -0.08446260 0.3854874 0.209316191 148.00205	29 6.590847e-18

4 Model 2	Mood 31	5 0.2983374 0.03335590 0.5633189 0.027336114 34.75919	30 2.515079e-01

>

> write.csv(

+	main_results,

+	"Main_Model_Results.csv",

+	row.names = FALSE

+ )

>

> model3_results <- data.frame(

+	Term = names(stats::coef(res3)),

+	Estimate = as.numeric(stats::coef(res3)),

+	SE = as.numeric(res3$se),

+	CI_lower = as.numeric(res3$ci.lb),

+	CI_upper = as.numeric(res3$ci.ub),

+	P_value = as.numeric(res3$pval),

+	stringsAsFactors = FALSE

+ )

>

> write.csv(

+	model3_results,

+	"Model3_Moderator_Results.csv",

+	row.names = FALSE

+ )

>

> # Save the exact V matrices for audit/reproduction.

> write.csv(V1_sleep, "V_Model1_Sleep.csv", row.names = TRUE)

> write.csv(V1_mood, "V_Model1_Mood.csv", row.names = TRUE)

> write.csv(V2_sleep, "V_Model2_Sleep.csv", row.names = TRUE)

> write.csv(V2_mood, "V_Model2_Mood.csv", row.names = TRUE)

> write.csv(V3_sleep, "V_Model3_Sleep.csv", row.names = TRUE)

>

> capture.output(

+	sessionInfo(),

+	file = "Session_Info.txt"

+ )

>

> cat("\n=========================================================\n")

=========================================================

> cat("ANALYSIS COMPLETE\n")

ANALYSIS COMPLETE

> cat("=========================================================\n")

=========================================================

> cat("Expected embedded-data counts:\n")

Expected embedded-data counts:

> cat(" Model 1 = 20 (Sleep 10; Mood 10)\n")

Model 1 = 20 (Sleep 10; Mood 10)

> cat(" Model 2 = 61 (Sleep 30; Mood 31)\n")

Model 2 = 61 (Sleep 30; Mood 31)

> cat(" Model 3 = 81 (Sleep 40; Mood 41)\n")

Model 3 = 81 (Sleep 40; Mood 41)

> cat("\nPrimary dependency assumptions:\n")

Primary dependency assumptions:

> cat(sprintf(" Paired/crossover r = %.2f\n", PAIRED_R_PRIMARY))

Paired/crossover r = 0.50

> cat(sprintf(" Sampling-error rho = %.2f\n", SAMPLING_RHO_PRIMARY))

Sampling-error rho = 0.50

> cat("\nKey output files include:\n")

Key output files include:

> cat(" Main_Model_Results.csv\n")

Main_Model_Results.csv

> cat(" Model3_Moderator_Results.csv\n")

Model3_Moderator_Results.csv

> cat(" Direct_Comparison_Pair_Subgroups_Sleep.csv\n")

Direct_Comparison_Pair_Subgroups_Sleep.csv

> cat(" Direct_Comparison_Pair_Subgroups_Mood.csv\n")

Direct_Comparison_Pair_Subgroups_Mood.csv

> cat(" Working_Correlation_Sensitivity_Models1_2.csv\n")

Working_Correlation_Sensitivity_Models1_2.csv

> cat(" Leave_One_Out_Sleep.csv\n")

Leave_One_Out_Sleep.csv

> cat(" Forest_Plot_Sleep.png\n")

Forest_Plot_Sleep.png

> cat(" Forest_Plot_Mood.png\n")

Forest_Plot_Mood.png

> cat(" Funnel_Plot_Sleep.png\n")

Funnel_Plot_Sleep.png

> cat(" Funnel_Plot_Mood.png\n")

Funnel_Plot_Mood.png

> cat(" V_Model1_Sleep.csv / V_Model1_Mood.csv\n")

V_Model1_Sleep.csv / V_Model1_Mood.csv

> cat(" V_Model2_Sleep.csv / V_Model2_Mood.csv\n")

V_Model2_Sleep.csv / V_Model2_Mood.csv

> cat(" V_Model3_Sleep.csv\n")

V_Model3_Sleep.csv

> cat(" Session_Info.txt\n")

Session_Info.txt

> cat("=========================================================\n")

=========================================================

>
